# Trimethylamine N-oxide and major adverse cardiac and cerebrovascular events: results of umbrella review and meta-analysis

**DOI:** 10.1186/s12986-026-01214-0

**Published:** 2026-09-23

**Authors:** Rima Obeid, Lorenz Mohr, Bryan A. White, Gunnar H. Heine, Insa Emrich, Juergen Geisel, R. Colin Carter

**Affiliations:** 1 Department of Clinical Chemistry and Laboratory Medicine, Saarland University Hospital, Homburg/Saar, Germany; 2 University of the Saarland, Medical Faculty, 66420 Homburg, Germany; 3 Department of Animal Sciences and Fellow American Academy of Microbiology, University of Illinois, 1207 W. Gregory Drive, Urbana, Il 6180 USA; 4 Agaplesion Markus Hospital, Medical Clinic II, Wilhelm-Epstein Straße 4, D-60431 Frankfurt am Main, Germany; 5 Department of Internal Medicine IV–Nephrology and Hypertension, Faculty of Medicine, Saarland University Hospital and Saarland University, D-66421 Homburg, Germany; 6 Department of Internal Medicine III, Cardiology, Angiology, and Intensive Care Medicine, Saarland University Medical Center, Homburg, Germany; 7 Departments of Pediatrics and Emergency Medicine and the Institute of Human Nutrition, Columbia University Irving Medical Center, New York, NY USA; 8 Columbia University Vagelos College of Physicians and Surgeons, New York, NY USA

**Keywords:** Trimethylamine N-oxide, Cardiovascular disease, Stroke, Risk factors, Renal function, Gut-bacteria, Metabolism

## Abstract

**Background:**

Circulating trimethylamine N-oxide (TMAO) may be a causal factor or marker for major adverse cardiac and cerebrovascular events (MACE), but evidence in humans is limited to observational studies with inherent uncertainties.

**Methods:**

We conducted an umbrella systematic review and meta-analysis of human studies. The search in MEDLINE, Web of Science, Cochrane Library and PROSPERO (last updated in February 2025) identified 120 publications that met our inclusion criteria.

**Results:**

The pooled hazard ratio (HR) and 95% confidence intervals (95%CIs) from 34 cohort studies on TMAO and MACE = 1.20 (1.12–1.27), the prediction interval (PI) was 0.93–1.54, and I^2^ = 73.3%. After correction for publication bias (*p* < 0.001 Egger’s test), the pooled HR (95%CIs) was 1.10 (1.03–1.17). The pooled odds ratio (95%CIs) from 18 case-control studies after accounting for publication bias was 1.05 (0.94–1.18). As a diagnostic test, the estimated pooled area under the curve (95%CIs) for TMAO was 72.8 (68.8–77.1)%, PI = 54.7%–97.1%, I^2^ = 93.8%, *n* = 24 studies. The mean (95%CIs) sensitivity and specificity of TMAO were 65.3 (56.1–74.5)% and 79.0 (65.4–92.6)%, respectively. The data did not suggest a threshold for elevated TMAO concentrations.

**Conclusions:**

Previously reported association estimates between TMAO and MACE in human studies are systematically overestimated. The results do not support TMAO being a causal factor or marker of MACE and related conditions. Harmonisation of TMAO measurements between labs and cohort studies in healthy people that address confounding factors remain critically needed.

**Supplementary Information:**

The online version contains supplementary material available at https://doi.org/10.1186/s12986-026-01214-0.

## Introduction

Trimethylamine N-oxide (TMAO) is a small molecule (molecular weight = 74 kD) that is produced in the liver from trimethylamine, a gut-bacteria-mediated metabolite of the nutrients choline, betaine and carnitine. TMAO has emerged as a potentially relevant causal factor that may have ethiological role in the disease process [1–3]. TMAO could alternatively function as a risk marker of major adverse cardiac and cerebrovascular events (MACE). In the latter case, the statistical association between TMAO and the outcome could be used to predict future adverse events or to diagnose a disease without TMAO being involved in the etiology of the disease [4]. The association between elevated plasma concentrations of TMAO and MACE has been studied in observational studies (reviewed in [5]). Almost all observational studies included people with known MACE risk factors (e.g., renal dysfunction and diabetes). These risk factors have been shown to be associated with elevated plasma TMAO [6, 7] and in the same time with increased risk of MACE, suggesting that common risk factors of MACE may confound the association between TMAO and MACE. Also physiological factors such as age, sex, gut bacteria and dietary intake of TMAO precursors may explain plasma concentrations of TMAO. A common issue in published observational studies on cardiovascular diseases [8] that include TMAO is lack of clarity regarding causal inference vs. predictive models (e.g [9–11]). There is currently insufficient evidence to determine whether elevations in TMAO are causally related to MACE and related outcomes.

Studies in rodents suggested that TMAO may promote atherosclerosis by affecting cholesterol metabolism [12] or platelet aggregation [13] or by inducing tubulointerstitial fibrosis and collagen deposition (thus acting as urinary toxins) [3]. However, whether such mechanisms are operating in humans remains speculative.

In the present systematic review and meta-analysis, our main objective was to investigate the associations between circulating TMAO and MACE and related outcomes (as defined in Table 1). We used established tools to study the robustness of the pooled estimates and identify potential sources of bias and heterogeneity in primary studies. Our second objective was to canvas published studies to examine the potential usefulness of TMAO as a biomarker to diagnose or predict MACE. In this manuscript, we consider “risk marker,” “prognostic marker” and “diagnostic marker” to be markers of association without clear link to causality. By contrast, “risk factor” is used when causation is inferred.

## Methods

### Search methods

The present study was registered in the International Prospective Register of Systematic Reviews (PROSPERO, CRD42024534940) before initiating the search. The population, intervention, comparator, and the outcomes of the present review are shown in Supplemental Table 1. The search was conducted in MEDLINE, Web of Science, Cochrane Library and PROSPERO (main search on 16-May-2024) and restricted to English language. An updated literature search was conducted on 23-February-2025 to identify primary publications that were not captured by the previous systematic reviews and meta-analyses (Supplemental Table 2). Reference lists of previous systematic reviews were also screened. The inclusion and exclusion criteria were recently described [5]. Briefly, we included studies of all designs and effect sizes on the associations between blood TMAO and MACE. Studies with serious methodological flaws were excluded. The search, screening, data extraction and evaluation were conducted by two authors (RO and LM).

### Study exposure and outcomes

The exposure of interest is TMAO concentrations measured in serum or plasma samples. We had no restriction with regard to TMAO assay, pre-analytical conditions or method performance.

MACE is a composite outcome with heterogeneous definitions in the literature, especially across observational studies [14]. We defined diseases that will be considered under MACE a priori in the protocol stage (Table 1). Exact information on the outcomes were extracted as they were defined in the original publications. After data extraction, the outcome definition was reviewed by authors RO and IE (an expert cardiologist). We judged it purposeful to focus on the composite outcome MACE rather than running separate meta-analyses for each single sub-outcome within MACE, e.g., myocardial infarction, cardiovascular mortality or stroke. The term MACE was used in a heterogeneous way in the literature on TMAO. MACE sometimes included outcomes such as all-cause mortality, and heart failure (HF) in addition to classical components (myocardial infarction, stroke and cardiovascular death).

**Table 1:** Definition of the outcomes in the present study

**Major adverse cardiac and cerebrovascular events (MACE) included the following diseases:**
• Myocardial infarction • Angina pectoris stable or unstable • Stroke, ischemic stroke • Cardiovascular death • Sudden cardiac arrest • Arrhythmia Revascularisation coronary/peripheral arterial • Coronary artery calcium • Cerebrovascular (bypass) • Amputation/limb • Mortality due to any of the previous conditions
**Heart failure (HF):** • Hospitalization for acute decompensated HF • Cardiac transplantation • Congestive HF
**Arterial fibrillation**
**All-cause mortality**
**Renal outcome:** as defined on the study level (see Table S2)

### Data extraction

The following information was extracted from the studies: First author, publication year, PMID, study objective, outcomes, methods used to ascertain the outcome, country of participants, name of the study, the recruitment time window, people characteristics, n participants, study design, follow up duration (in cohort studies), number of people with the outcome, definition of elevated TMAO concentrations on the study level, adjusted and non-adjusted effect size and 95% Confidence Intervals (95%CIs), adjustment variables, and study funding. In a protocol amendment, we also collected data from receiver operating characteristic (ROC)-curve analyses on area under the curve (AUC) and sensitivity and specificity of TMAO at a given cutoff value.

### Data analyses

Data analyses were carried out using Comprehensive Meta-Analysis Version 4. We collected summary data on adjusted and unadjusted hazards ratio (HR), odds ratio (OR), and relative risk (RR), and their 95%CIs using direct methods (as reported in the primary studies). Because adjusted effect sizes were our main focus, we did not compute effect sizes from number of events or survival curves. Data analyses were conducted using the reported effect size per unit increase of TMAO or by combining effect size from all reported TMAO concentration ranges versus the lowest category (i.e., the reference group).

Publications with the same design (i.e., cohort, case-control, or cross sectional) and effect size (i.e., HR, OR, or RR) were combined in a meta-analysis if they address the same outcome or composite outcome. A meta-analysis was performed when at least three studies were available on the same outcome and effect size.

All meta-analyses were conducted using the random-effects model, and Forest plots were used to visualize the results. The primary data analyses relied on the adjusted effect size. The unadjusted effect size was used only in sensitivity analyses that aimed to estimate the effect of confounding on the pooled estimates. An estimate of the log-hazard ratio and variance pooled across trials was calculated. The inverse variance method was used to weigh the effect size in the individual studies. We used the Hartung-Knapp method for making inferences about the average effect when fitting the random‐effects model. The Hartung‐Knapp adjustment increases the precision in the between‐study variance and the within-study variance (due to correlations when studies are reporting multiple subgroups). The Hartung‐Knapp method uses quantiles of the *t* distribution rather than the standard normal distribution when computing the 95%CIs for the average effect [15]. To study heterogeneity, we used the Cochran’s Q test that investigates the null hypothesis that all studies in the analysis share a common effect size or all effects are equal. The I-square (I^2^) is reported as a measure of heterogeneity or the percentage of the variance in the observed effects that reflects variance in true effects rather than sampling error. The pooled effect size and heterogeneity are calculated completely separately, and high heterogeneity of studies is generally interpreted as calling into question the validity of the pooled estimate.

The prediction interval (PI) was computed assuming that the true effects are normally distributed on a log scale. The PI estimates the range where the expected true effects for 95% of similar (exchangeable) future studies may be [16]. In contrast to heterogeneity analyses, a PI evaluates the variability of the data over different settings. If the studies show high heterogeneity, a PI covers a wider range than the 95%CIs, meaning that conclusions based on 95%CIs will not hold for all settings, especially when the heterogeneity between the studies is high [16].

Data analyses were conducted on the study level, meaning that the study was the unit of the analyses and contributed only once to the meta-analysis. The only two exceptions for this rule were: first, when a publication reports the results among two independent groups, the effect size from both groups contributed to the meta-analyses as if they originate from two studies. Second, multiple publications with major overlap in the participants were merged and contributed to the meta-analyses as if they were a single study to limit the impact of selective over-reporting.

### Risk of bias assessment

The risk of bias (ROB) assessment was conducted by using an individualized version of the Office of Health AssessMt and Translation (OHAT) tool of the Division of the National Toxicology Program, National Institute of EnvironMtal Health Sciences (Supplemental Table 3). The OHAT tool addresses the following bias domains: 1- selection of study participants and appropriate comparison groups, 2- accounting for important confounding and modifying variables by design or data analyses, 3- completeness of outcome data and attrition or exclusion from analysis, 4- confidence in the exposure characterization, 5- confidence in the outcome assessment, 6- selective reporting, and 7- other potential threats to internal validity (e.g., statistical methods were appropriate and researchers adhered to the study protocol). The ROB assessment for each of the seven domains can be evaluated as; definitely low, probably low, definitely high, and probably high. We considered definitely low and probably low as “low” ROB and definitely high and probably high as “high” ROB. For subgroup analyses we studied the pool estimates according to the ROB (low versus high ROB) in each domain and according to the overall ROB (either low or high). A study received a “low” overall ROB when up to three domains of OHAT (out of 7 domains) were scored as having high ROB. A “high” overall ROB was defined when ≥ 4 domains were scored as having high ROB.

### Publication bias

Publication bias was studied by Egger’s regression test and Funnel plots. Duval and Tweedie trim-and-fill method to correct for missing studies, and we reported the publication bias-adjusted pooled estimates. The presence of publication bias may show whether higher plasma TMAO could be associated with MACE because some studies with negative results were unpublished.

### Sensitivity and subgroup analyses

We conducted leave-one-out meta-analyses by excluding each publication, one at a time, to examine whether a single study was driving the overall association estimate. In sensitivity analyses, we investigated factors affecting the direction and the strength of the association. Sensitivity analyses included also replacing overlapping publications and using the effect size at a different follow up visit. Moreover, we meta-analyzed the unadjusted effect size to estimate the extent to which adjusting for confounders on the study level affected the pooled estimates. We expected large heterogeneity across the studies and planned the following subgroup analyses to identify sources of heterogeneity between the studies: geographical origin of the participants, duration of follow up in cohort studies, ROB (overall and in each domain of the OHAT tool), adjustment for renal function (yes vs. no), and adjustment for hypertension (yes vs. no).

## Results

### Search results

The search on 16 May 2024 identified 27 relevant systematic reviews and meta-analyses that were screened to identify primary publications on the topic. This screening yielded 451 primary publications. After screening the 451 publications and removing the duplicates, 175 potentially relevant primary publications retained for full text screening and then 104 publications were eligible for data extraction. We excluded 17 (of 175) publications due to Chinese language and 54 publications due to meeting at least one of our exclusion criteria. A detailed list of excluded studies and reasons for exclusions are shown in Supplemental Table 4.

An updated search was conducted on 23 February 2025 and led 1597 publications that were eligible for title- and abstract-screening. Subsequently, 126 candidate full texts were screened. After removing 62 publications that were already identified through the previous search, 64 full text publications were screened and 36 novel publications were identified through the updated search. Thus, a total of 141 publications with unique identifier number in MEDLINE (PMID) on all outcomes were identified (Study Flow Diagram, Supplemental Fig. 1). After excluding 21 articles mainly due to inappropriate effect size (that can not be combined with other studies), 120 publications with unique PMIDs were candidates for data analyses (Supplemental Excel Table). For quantitative data analyses, the publications were grouped according to study designs and outcomes.

We found several publications with overlapping study populations and outcomes between papers. Overlap designation was based on names of the authors, hospital/city where people were recruited, time periods of recruitment and characteristics of the participants. Publications judged to be overlapping were combined as one study before computing the pooled estimate from independent publications. Established population studies with 70 unique PMIDs contributed to the literature on TMAO in relation to our planned outcomes (Supplemental Excel Table). The GenBank study/Cleveland Clinic, USA contributed 11 distinct publications [3, 13, 17–24]; and the Third China National Stroke Registry (CNSR-III) contributed 4 publications [25–28]. The Cardio-vascular Health Study (CHS) and the Multi-Ethnic Study of Atherosclerosis (MESA) study [29–31], the Prevention of REnal and Vascular ENd-stage Disease (PREVEND) study [32, 33], the Prevention With Mediterranean Diet trial (PREDIMED) [34, 35] and other overlapping studies [10, 36–39] had multiple publications. For the data analyses, we combined overlapping publications with the same effect size on the same outcome to reduce the effect of selective over-reporting from studies that were likely to find positive associations (reporting bias).

Most publications reported more than one outcome and did not specify a primary outcome. In the current manuscript, we focus on the association between TMAO concentrations and MACE. In addition, we analyzed data on the diagnostic utility of TMAO concentrations because of its potential translational meaning and practical relevance. All other results on other planned outcomes are reported in Supplemental Data File (1).

### TMAO concentrations and MACE in cohort studies

The association between TMAO concentrations and MACE was investigated in 37 publications with unique PMIDs and a longitudinal study design. Three of the 37 studies were excluded from the analyses because no HR is reported (Tan et al., and Wu et al., reported OR and Meyer et al., reported RR) [40–42]. Table 2 briefly describes the 34 publications that were included in a meta-analysis [9, 25–28, 31, 32, 43–68]. Overlapping publications were combined in one estimate before running the meta-analysis; 4 publications in the CNSR-III cohort [25–28] and 2 publications by Li et al. [46, 49]. Bjornestad et al. [48], Flores-Guerrero et al. [32], and Shafi et al. [65], reported data from non-overlapping groups that contributed to the meta-analysis as separate studies.

**Table 2:** Brief description of 34 cohort studies on the association between TMAO and major adverse cardiac and cerebrovascular events (MACE). The definition of MACE in the present study is shown in Table 1

Citation	PMID	The outcome as reported	Characteristics^1^	*N*, follow up (FU)
Lever, 2014 [67]	25493436	MI Unstable angina	Age 47–93y; 73% M, hospital admission due to an acute coronary event. Prevalent CVD risk factors	*n* = 475, Med. (IQR) = 4.9 (4.0–5.5)y
Bjørnestad, 2022 [48]	35916742	CV-mortality	mean age **WECAC** 62y, 72% M; **HUSK**: ≈ 60y, 45% M. Prevalent CVD risk factors in both cohorts.	WECAC: *n* = 4132, mean (SD) FU 9.8 (2.6)y. HUSK: *n* = 6393, FU 10.5 (1.9)y
Lee, 2021 [55]	34398665	1st occurrence and recurrent CVD: fatal and nonfatal MI, fatal coronary heart disease, stroke (fatal and nonfatal), sudden cardiac mortality, and other atherosclerotic mortality	Age ≥ 65y, 36% M, 16% Black race, prevalent CVD risk factors	*n* = 4131 first CVD; *n* = 1449 recurrent CVD, Med. FU 15y; max. 26y
Müller, 2015 [68]	26554714	Major adverse CV-events (cardiac mortality, MI, or stroke)	Age 63 (55–71)y; 68% M, undergoing coronary angiography for suspected CAD, prevalent CVD risk factors	*n* = 339, FU 8y
Stubbs, 2019 [62]	30665924	Vascular composite (CV-mortality, MI, hospitalization for unstable angina, Prosthetic valve endocarditis, stroke)	Mean age 54y, 60% M, on maintenance HD with prevalent CVD risk factors	*n* = 1243, FU 64mo.
Winther, 2019 [61]	31123156	Incidence of combined CVD (comprising CVD–related mortality, ischemic heart disease (including nonfatal MI), nonfatal stroke, coronary interventions, or peripheral arterial interventions (including amputations)	Mean age 46y, 58% M, all have type 1 diabetes. Prevalent CVD risk factors	*n* = 1159, Med. (IQR) FU 8.7 (5.1–16.3)y
Lemaitre, 2023 [31]	37581385	Incident ischemic stroke	Mean age 39–40y, 35–50% M, White and Black race	*n* = 3444, FU 19y
Winther, 2021 [56]	33657115	CVD event CV–related mortality	Age 57 (8)y, 75% M, with T2DM and albuminuria	*n* = 311, Med. (IQR) FU 6.5 (5.5–8.1)y for CVDs; 6.8 (6.1–15.5)y for mortality
Obeid, 2024 [43]	38621431	CV-event including CV–mortality (excluding non–CV-mortality)	Mean age 65y, 60% M, CKD stage 2–4, prevalent risk factors for CVD	*n* = 478, mean (SD) FU 5.1 (2.1)y
Li, 2022 [46]	36354779	Recurrent MI; Revascularization; Stroke	Age 61y, 80% M, acute MI and prevalent CVD risk factors	*n* = 1203, Med. FU 739 days
Li, 2022 [49]	35686339	Recurrent MI	Age 63 (54, 70)y, 78% M with acute MI and HF. Prevalent CVD risk factors	*n* = 985, Med. FU 716 days
Shafi, 2017 [65]	27436853	*Cardiac mortality*: due to coronary events, arrhythmias, sudden cardiac mortality, congestive HF, or cerebrovascular events. *Sudden cardiac mortality*: witnessed mortality with preceding duration of symptoms less than 24 h or unwitnessed mortality, or unwitnessed unexpected mortality with symptom duration less than the interval since the last dialysis	Age 58y, 43% M, on HD and prevalent risk factors such as diabetes, CVD, hypertension	*n* = 1232, Med. (IQR) FU 2.3 (1.1–3.9)y
Kim, 2016 [9]	27,083,288	Ischemic event (MI, unstable angina, ischemic stroke, coronary revascularization, new onset of CHD (proven by cardiac catheterization), amputation due to peripheral vascular disease, peripheral artery bypass, and gangrene)	Age mean (SD) 68 (13)y, 63% M, all with CKD, prevalent diabetes, ischemic heart disease, HF, obesity, hyperlipidemia	*n* = 2529, FU 3y
Luciani, 2023 [45]	36593094	1)- Ischemic stroke. 2)- CV-mortality included cardiac mortality (e.g., cardiogenic shock, arrhythmia/sudden mortality, cardiac rupture) and other vascular mortalities (e.g., stroke, pulmonary embolism, ruptured aortic aneurysm or dissection) and hemorrhagic mortalities	Age 70-75y, > 70% M, with AF, prevalent risk factors	*n* = 1722, Med. (IQR) FU 4 (3–5)y
Flores-Guerrero, 2021 [32]	33993608	CV-mortality	Mean (SD) age 52–57 (11)y; 41–66% M. Prevalent CVD risk factors	*n* = 5292, Med. (IQR) FU 8.2 (5.7–11.8)y
Xu, 2022 [28]	34794334	MACE: CV-mortality, nonfatal MI, and nonfatal stroke	Age 63 (54–70)y, 68% M, recruited within 7d of onset of symptoms of stroke, prevalent CVD risk factors	*n* = 10,756 (n with TMAO measured?), FU 1y
Xu, 2021 [27]	34816729	MACE: nonfatal stroke recurrence, nonfatal MI, or CV-mortality	Age 62-64y, > 50% M, with acute stroke, prevalent CVD risk factors	*n* = 10,027 (n with TMAO?), FU 1y
Xu, 2022 [26]	35390802	Recurrent stroke (new ischemic stroke or hemorrhagic stroke)	Age 63 (11)y, 70% M, hospitalized due to acute ischemic stroke, recruited within 7d of symptom onset, prevalent CVD risk factors	Data analyses restricted to *n* = 4186, FU 367 (363–381) days
Xue, 2022 [25]	36193936	Stroke recurrence (new ischemic and hemorrhagic stroke)	Age Med. (IQR) 62 (54–70)y, 71% M, with stroke or TIA recruited within 7d of the event, prevalent CVD risk factors	*n* = 9793, FU 1y
Shea, 2024 [44]	37865185	Incident CVD events	Mean age 39–40y, ≈ 40% M, White and Black race, prevalent CVD risk factors	*n* = 3444, FU 19y
Fu, 2021 [57]	33596582	CV-mortality due to MI, cardiac arrhythmia, cardiac arrest, congestive HF, cerebro-vascular events, and peripheral vascular disease	Mean age 48 (14)y, 57% M, > 3 mo peritoneal dialysis, revalent CVD risk factors	*n* = 1032, Med. (IQR) FU 64 (44–87) mo
Dai, 2022 [47]	36166845	First MACE incidence: a hospitalization or comorbidity due to cerebrovascular disease, MI, peripheral vascular disease, angina pectoris, arrhythmias, coronary artery disease, or mortality due to myocardial ischemia and infarction, cardiac arrest, or cerebrovascular accident	Age 76y, 63% M, eGFR ≤ 20 mL/min/1.73 m^2^ during the last 6 mo, prevalent CVD risk factors	*n* = 737, Med. FU 39 mo
Croyal, 2020 [59]	32301490	MACE: a composite of CV-mortality, nonfatal MI, or nonfatal stroke	Age 61–67y, 60% M, prevalent CVD risk factors	*n* = 1463, Med. (IQR) FU 85 (75) mo.
Skagen, 2016 [66]	26868510	Mortality from vascular event (stroke and MI)	Age 68 (8)y, 69% M, carotid stenosis, prevalent risk factors (especially in those who died)	*n* = 264, mean (SD) FU 5.3 (2.9)y (range 1–11y)
Sapa, 2022 [50]	35351578	CV-mortality due to MI, stroke, sudden mortality, HF, other cardiac events, non-cardiac CVD, or pulmonary embolism	Mean age 70y, 53% Black, 47% M, with diabetes, eGFR < 60 ml/min/1.73m^2^. Prevalent CVD risk factors	*n* = 555, mean (SD) FU 6.2 (3.5)y
Amrein, 2022 [52]	35220448	MI CV-mortality	Med. (IQR) age 69 (61–77)y, 66% M, suspected exercise induced myocardial ischemia. Prevalent CVD risk factors	*n* = 1726, FU 5y
Flores-Guerrero, 2021 [69]	34073908	CV-mortality	Mean (SD) age 69 (11.2)y, 42% M. Prevalent CVD risk factors	*n* = 594, Med. (IQR) FU 10.4 (5.7–11.8)y
Schuett, 2017 [64]	29268932	CV-mortality	Mean age 65y, 70% M, underwent coronary angiography. Prevalent CVD risk factors	*n* = 2490, mean 9.7y
Chang, 2022 [54]	34724845	CVD mortality due to MI, congestive HF, cerebral bleeding, cerebral infarction, arrhythmia, peripheral arterial disease and sudden mortality	Mean age 54y, 58% M, on peritoneal dialysis. Prevalent CVD risk factors	*n* = 513, Med. (IQR) 35 (16–65) mo.
Roncal, 2019 [60]	31666590	CV-mortality	Mean age 70y, 87% M, with PAD, prevalent CVD risk factors	*n* = 262, mean (range) FU 4y (1–102) mo
Zhang, 2020 [58]	32985309	CV-mortality	Mean age 57y, 56% M, on HD for ≥ 6 mo. Prevalent risk factors	*n* = 252, Med. (IQR) 73.4 (42.9–108) mo.
Haghikia, 2018 [63]	29976769	Combined vascular end–point composed of MI, recurrent stroke and CV-mortality	Age 67y, 61% M, with 1st acute stroke onset in the last 7d, prevalent CVD risk factor	*n* = 608 (n for data analysis = 593), FU 1y
Chen, 2022 [51]	35345908	Major vascular event (TIA, recurrent ischemic stroke, acute MI) within the first three months of stroke	Age 62 (7)y, 55% M, large artery atherosclerotic ischemic stroke within 72 h of symptom start. Prevalent CVD risk factors	*n* = 291, FU 3 mo.
Eyileten, 2021 [53]	34778397	CV-mortality including mortality from an acute MI, sudden cardiac mortality, and death due to HF, stroke, CV procedures, CV hemorrhage or other CV causes	Age 59 (12)y, 80% M, acute coronary events at admission to the hospital. Prevalent CVD risk factors	*n* = 292, FU 7y

^1^ Prevalent CVD risk factors; e.g., diabetes, hypertension, smoking, previous CVD

Abbreviations used in this table: AF, arterial fibrillation; CAD, coronary artery disease; CKD, chronic kidney disease; CV, cardio-vascular; CVD, cardio-vascular disease; eGFR, estimated glomerular filtration rate; HD, hemodialysis; HF, heart failure; FU, follow up; IQR, interquartile range; M, male sex; Med., median; MI, myocardial infarction; mo, months; PAD, all had peripheral arterial disease; SD, standard deviation; TIA, transient ischemic attack

**Fig. 1:**
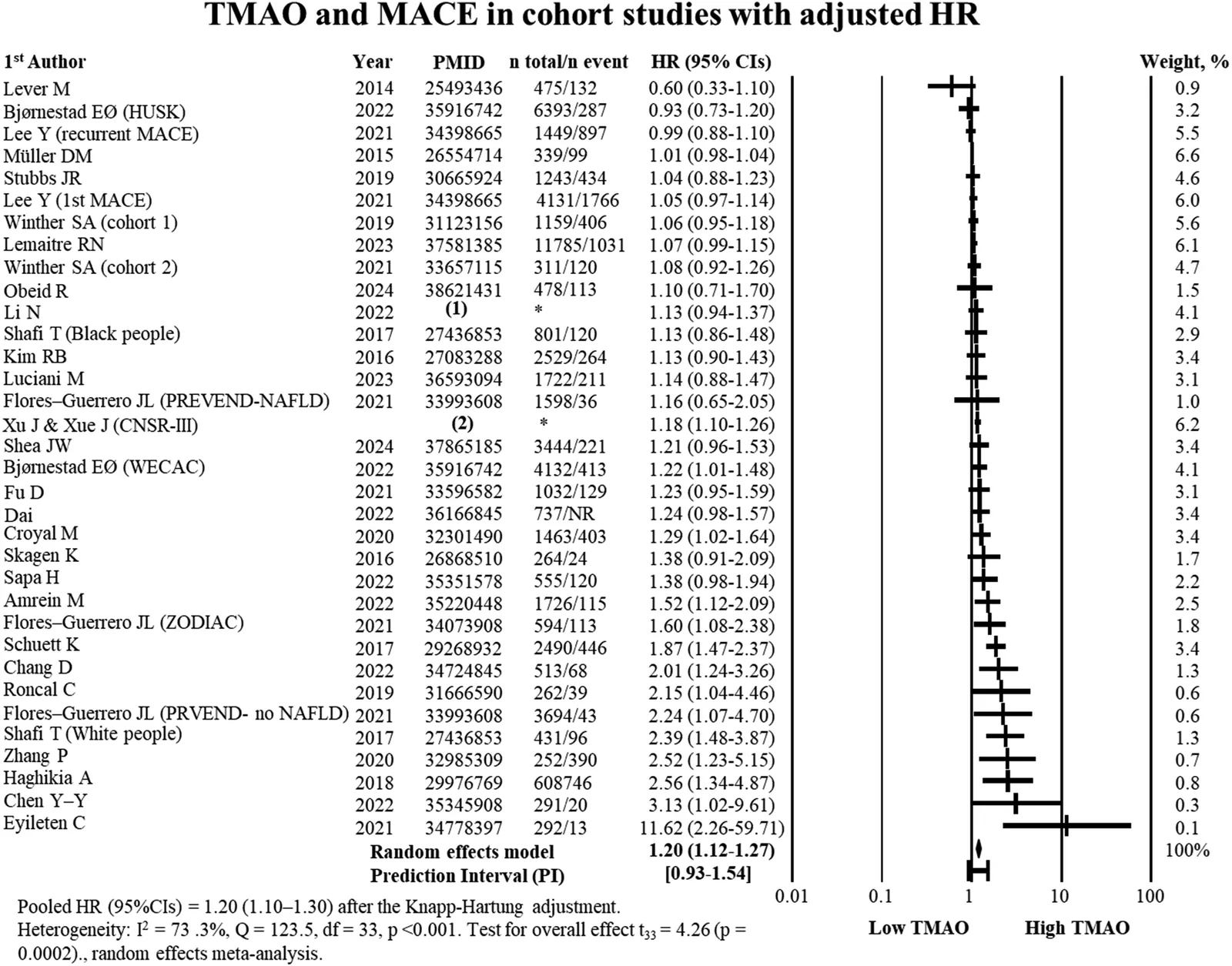
Forest plot for the association between circulating TMAO and MACE in cohort studies. Direct methods were used to extract adjusted HR (95%CIs) from the original publications. The studies are ordered according to HR (lowest to highest). (1) Overlapping publications by Li et al., were merged [PMIDs: 35686339 and 36354779, * n total/n event: 985/138 and 1203/307, respectively]. (2) Publications based on CNSR–III register were merged [PMIDs: 35390802, 34816729, 36193936, and 34794334,* n total/n event: 4186/215, 1027/not reported (NR), 9793/2357, and 10756/1114, respectively]

Meta-analysis of cohort studies (*n* = 34 described in Table 2) that reported adjusted HR (95%CIs) of the association between TMAO concentrations and MACE showed a pooled HR (95%CIs) of 1.20 (1.12–1.27) (Fig. 1). The Q-statistic showed a Q-value of 123.5, degrees of freedom (df) = 33, *p* < 0.001 and an I-squared (I^2^) = 73.3%. Thus, 73.3% of the variance in observed effects reflects variance in true effects rather than sampling error, suggesting high heterogeneity between the studies. The pooled HR (95%CIs) after Knapp-Hartung adjustment was 1.20 (1.10–1.30). The PI was 0.93–1.54.

**Fig. 2:**
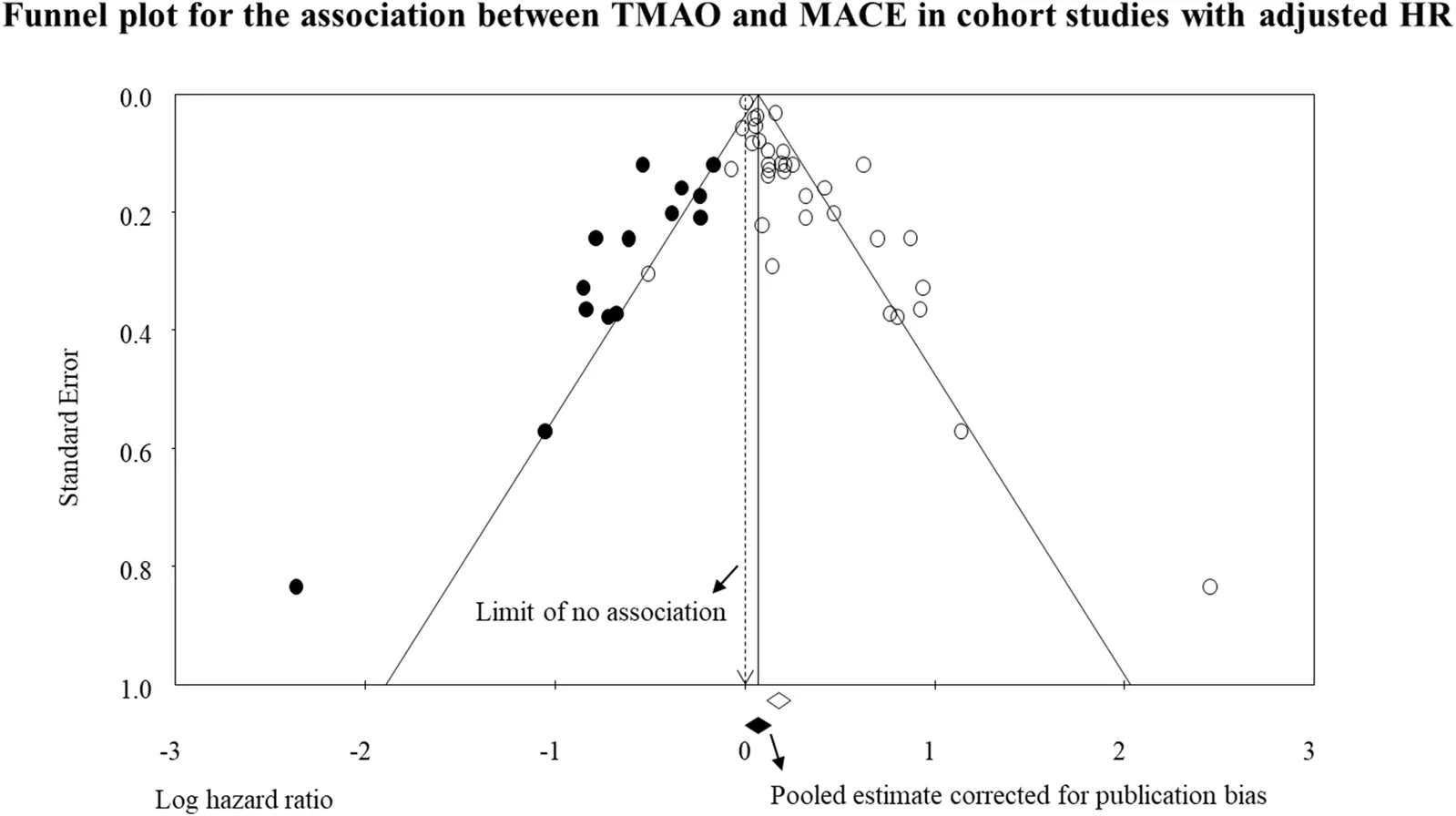
Funnel plot of standard error by log-HR to study publication bias of the association between circulating TMAO concentrations and MACE in cohort studies. The Egger’s test supports the presence of funnel plot asymmetry (*p* < 0.001). After accounting for 14 missing studies (the filled circles), the pooled HR (95%CIs) moved towards the null 1.07 (1.03–1.15)

The Egger’s test indicated a significant publications bias (*p* < 0.001). The Duval and Tweedie trim-and-fill method suggested that 14 studies all on the left side of the funnel plot may be missing. After correcting for publication bias, the pooled HR (95%CIs) moved towards the null = 1.07 (1.03–1.15). The Knapp-Hartung adjustment abolished the association HR (95%CIs) = 1.07 (0.96–1.20) and the PI 0.77–1.49 also suggested that TMAO and MACE may not be associated in all similar future studies (Fig. 2).

#### Sensitivity analyses of cohort studies on TMAO and MACE

Excluding any of the 34 cohort publications in leave-one-out analyses did not have a meaningful effect on the pooled estimate (Supplemental Fig. 2). Furthermore, the pooled HR (95%CIs) was almost unchanged whichever of the 4 CNSR-III-based studies was entered into the meta-analyses [25–28] (data not shown).

In further sensitivity analyses we aimed to meta-analyze the unadjusted HR (95%CIs) to estimate the proportion of the association that can be explained by confounding. The unadjusted HR (95%CIs) were not reported in 3 publications [44, 64, 68]. The remaining 31 publications showed a pooled unadjusted HR (95%CIs) of 1.60 (95%CI: 1.45–1.76), PI = 0.99–2.57, df = 30, I^2^ = 88.1%. The pooled HR (95%CIs) after Knapp-Hartung adjustment were 1.60 (1.37–1.85). The Egger’s test (*p* < 0.001) suggested the presence of publication bias and 8 studies could be missing (Supplemental Fig. 3). After correction for publication bias, the pooled unadjusted HR (95%CIs) was 1.31 (1.18–1.46) (Funnel plot in Supplemental Fig. 4).

#### Subgroup analyses of cohort studies on TMAO and MACE

The majority of the studies showed high ROB in one or more of the 7 domains of the OHAT tool. In subgroup analyses, we found that studies with a higher overall ROB estimated larger magnitude associations between TMAO and MACE [HR (95%CIs) = 1.25 (1.13–1.38), *n* = 19] compared to studies with a lower overall ROB [HR (95%CIs) = 1.15 (1.08–1.23), *n* = 17]. Studies with high ROB (e.g., in selection, confounding, attrition, and detection bias of the exposure and the outcome) also showed higher pooled HRs compared to those from studies with low ROB (Supplemental Table 5). Studies with low ROB not only showed weak association, but also less heterogeneity across the studies.

Studies with earlier publication years (2016–2020 vs. 2021–2024) and a shorter follow up duration (< 5 years vs. ≥ 5 years) showed higher pooled HR than those published later or had a longer follow up time, respectively (Supplemental Table 6). The primary studies adjusted for different sets of potential confounders. Adjustment for a marker of renal function or hypertension had low impact on the pooled HR (95%CIs) (Supplemental Table 6). The association between TMAO and MACE was stronger in studies from China than those from other parts of the world.

Subgroup analyses of 11 studies including people with pre-existing kidney diseases at baseline visit (e.g., kidney replacement therapy, chronic kidney disease, or peritoneal dialysis) showed similar pooled HR (95%CIs) of 1.26 (1.11–1.43) and 1.26 (1.06–1.49) after Knapp-Hartung adjustment, I^2^ = 55.0%. This value is close to that from populations with normal renal function at baseline visit with HR (95%CIs) of 1.18 (1.10–1.26) and 1.18 (1.06–1.31) after Knapp-Hartung adjustment, I^2^ = 76.6%, *n* = 23 studies (Supplemental Fig. 5).

#### Dose-response pattern analysis between TMAO cutoff values and HR for MACE

Various potential “abnormal” or “risk-identifying” cutoff values were used in individual cohort studies to define elevated circulating TMAO concentrations. We studied whether higher TMAO cutoff values were related to higher HR for MACE which may suggest a dose-response pattern between TMAO levels and MACE risk. TMAO separated by median, tertiles, quartiles or any otherwise defined cutoff values were plotted against the corresponding HR for MACE. Supplemental Fig. 6 does not suggest a dose-response relationship between TMAO cutoff values and Log(confounder adjusted HR) of MACE across all sub-studies.

### TMAO concentrations and MACE in case-control studies

Out of 29 publications with a case-control study design, 4 publications did not report OR (95%CIs) [11, 66, 70, 71]; and 25 publications reported adjusted OR (95%CIs) for MACE (*n* = 18) [51, 72–88], heart failure (*n* = 4), arterial fibrillation (*n* = 1), renal insufficiency (*n* = 1), and all-cause death (*n* = 1). Meta-analysis of the 18 case-control studies that reported adjusted OR (95%CIs) of the association between TMAO and MACE yielded a pooled OR (95%CIs) of 1.38 (1.24–1.54) and 1.38 (1.10–1.74) after Knapp-Hartung adjustment, PI = 0.95–2.02, Q-value = 155.5, *p* < 0.001, and high heterogeneity (I^2^ = 89.1%) (Supplemental Fig. 7).

The Egger’s test was significant (*p* = 0.0003) and the funnel plot showed publication bias that overestimated the association. After correcting for 9 theoretically missing studies, the pooled OR (95%CIs) was 1.05 (0.94–1.18) and 1.05 (0.79–1.40) after Knapp-Hartung adjustment (Supplemental Fig. 8). Excluding one study each time did not reveal a change in the overall estimate, suggesting that the pooled estimate was not biased due to a single study.

Case-control studies with higher overall ROB showed a significant association between TMAO and MACE [pooled OR (95%CIs) = 1.93 (1.40–2.66), I^2^ = 87.0%, *n* = 11]. The pooled estimate from studies with lower overall ROB was lower, with 95% confidence intervals that included 1.00 [pooled OR (95%CIs) = 1.08 (1.00–1.17), I^2^ = 77.7%, *n* = 7] (Supplemental Table 7).

Case-control studies that did not adjust for hypertension (*n* = 6) provided a pooled OR (95%CIs) = 2.32 (1.21–4.43) that was considerably higher than that from 12 studies that adjusted for hypertension = 1.24 (1.09–1.41) (Supplemental Table 7). Meta-analysis of the studies that adjusted for renal function (*n* = 11) yielded lower pooled OR (95%CIs) = 1.29 (1.13–1.49) compared to that from 7 studies that did not adjust for renal function 1.55 (1.29–1.86) (Supplemental Table 7). The results confirm that hypertension and renal function are important confounders of the association between TMAO and MACE.

Case-control studies that were conducted in Asia/China showed higher pooled estimate and heterogeneity [OR (95%CIs) = 2.02 (1.41–2.89), I^2^ = 92.0%, *n* = 9 studies from China and 1 study from Singapore] [51, 73, 74, 76, 79, 80, 84–87] compared to studies that were conducted in all other countries except Asia/China [OR (95%CIs) = 1.14 (1.002–1.29), I^2^ = 71.7%, *n* = 8)]. Regression of logOR on the cutoff reported to define elevated TMAO did not show significant association (Supplemental Fig. 9), suggesting no dose-response association between TMAO threshold and logOR.

### TMAO as a diagnostic marker; receiver operating characteristic (ROC)-curve analyses

ROC-curve analyses were used in 30 publications to derive AUC for TMAO concentration as a proposed diagnostic or prognostic test (i.e., to discriminate people with MACE or MACE-related outcomes from those without this outcome). The confidence intervals of the AUC were not reported in 5 publications [63, 89–92]. The remaining 25 publications reported using TMAO as a diagnostic/prognostic test in 39 occasions [40, 41, 45, 51, 53, 60, 72, 79, 85, 87, 93–107] (Table 3). Higher AUC indicates better performance in discriminating between people with and without a disease, which also translate into higher test sensitivity (identifying the patients who are positive for the outcome as such) and specificity (identifying the non-patients as such).

We meta-analyzed the AUCs on the study level; thus, the AUC of different outcomes from the same publication were combined in one estimate before combining AUCs from different publications. The pooled AUC (95%CIs) from 24 studies on MACE was 72.8% (68.8–77.1)%, PI = 54.7–97.1%, I^2^ = 93.8%). The mean (95%CIs) of sensitivity and specificity across the studies were 65.3% (56.1–74.5)% and 79.0% (65.4–92.6)%, respectively. Therefore, elevated TMAO test results have 65% probability of correctly identifying the true positive among people who are known to have the disease and 79% of negative test results out of all truly negative samples are free of the outcome. When the prevalence of elevated TMAO is 25% (i.e., > 4th quartile of a given TMAO distribution) in a given population, the probability that people with a positive TMAO test result indeed do have the condition of interest (i.e., positive predictive value, PPV (95%CIs)) = 50.8 (40.7–60.8)%. The probability that the disease is not present when the TMAO test is negative (i.e., the negative predictive values, NPV (95%CIs)) = 87.1 (83.6–90.0)%.

The sensitivity and specificity are computed for a specific cutoff value in a given study. There was no consistency in the cutoff values that were used to define a threshold of TMAO that may be associated with high risk of any outcome. For example, the following cutoff values were reported to predict stroke; 8.1 µmol/L [105], 5.0 µmol/L [103], 3.5 µmol/L [85], and 1.4 µmol/L (107 pg/ml) [51] (Table 3).

**Table 3:** Publications reporting receiver operating characteristic (ROC)-curve analyses and areas under the curve (AUCs) for TMAO concentration as a proposed diagnostic or prognostic test for at least one outcome

Citation	PMID	Design	TMAO for discrimination or predicting	TMAO cutoff^b^	Sn%^a^	Sp%^a^	AUC (95%CIs)
**MACE and MACE severity**							
Rexidamu, 2019 [105]	31142624	CS?	ischemic stroke vs. no stroke	8.1 µM	36.9	96.1	0.67 (0.62–0.71)
Liang, 2019 [85]	30370581	CC	ischemic stroke vs. no ischemic stroke in people with arterial fibrillation	3.5 µM	75	92.8	0.92 (0.88–0.96)
Luciani, 2023 [45]	36593094	CO	any stroke (5 y)	NR			0.54 (0.48–0.64)
Luciani, 2023 [45]	36593094	CO	ischemic stroke (5 y)	NR			0.52 (0.44–0.59)
Wu, 2018 [41]	29540587	CO	ischemic brain lesions after carotid artery stenting	4.3 µM	61.5	74.8	0.71 (0.64–0.77)
Zhai, 2019 [104]	31332666	CO	poor clinical outcome after ischemic stroke	3.9 µM	60.3	65.1	0.63 (0.56–0.70)
Zhai, 2019 [104]	31332666	CO	mortality after stroke (at 3 mo.)	4.0 µM	64.1	61.4	0.69 (0.60–0.77)
Wu, 2020 [103]	31907287	CS	acute ischemic stroke vs. controls	5.9 µM	49.1	94	0.73 (0.67–0.78)
Wu, 2020 [103]	31907287	CS	severe and moderate stroke vs. minor stroke	5.0 µM	70.2	79.9	0.79 (0.75–0.84)
Tan, 2020 [40]	32082246	CO	major ischemic events after 90 days	NR			0.72 (0.61–0.83)
Tan, 2020 [40]	32082246	CO	predict major ischemic events after 12 mo.	NR			0.76 (0.66–0.85)
Zhang, 2021 [101]	33647820	CO	poor functional outcome after stroke (mRS score 3–6)	5.8 µM	57.5	74.9	0.78 (0.72–0.83)
Li, 2023 [100]	35020253	CS	stroke versus control	NR			0.73 (0.65–0.80)
Li, 2023 [100]	35020253	CS	severe to moderate stroke vs. minor stroke	3.2 µM	65.5	70	0.69 (0.59–0.79)
Chen, 2022 [51]	35345908	CO	stroke and controls	1.4 * 10^− 3^ µM (107 pg/ml^c^)	63.2	80	0.78 (0.75–0.82)
Roncal, 2019 [60]	31666590	CO	disease severity (intermittent claudation vs. critical limb ischemia)	2.3 µM	62	76	0.73 (0.67–0.79)
Haghikia, 2018 [63]	29976769	CO	cardio-vascular risk at 1-y	4.9 µM	63	66	0.66 (95%CI NR)
Dong, 2018 [87]	30159101	CC	CHD without T2DM vs. controls	NR			0.93 (0.90–0.95)
Dong, 2018 [87]	30,159,101	CC	CHD with T2DM vs. controls	NR			0.79 (0.75–0.84)
Sheng, 2019 [107]	30594289	CS	high atherosclerotic burden (Synthax score > = 23)	NR			0.66 (0.59–0.72)
Huang, 2023 [96]	37755998	CS	differentiate between carotid–femoral pulse wave velocity > 10 m/s vs. < 10 m/s	1.6 µM (119 µg/L^c^)	73.8	64.4	0.75 (0.66–0.83)
Zhao, 2023 [97]	36811471	CO	predict MACE	2.9 µM	69.6	54.8	0.62 (0.56–0.68)
Yuan, 2023 [90]	36549408	CO	MACE vs. no MACE	NR			0.68, *p* < 0.01 (95%CI NR)
He, 2022 [91]	36384389	CS	identify vascular calcification high AAC score 5.5	105.2 µM (7.9 µg/ml^c^)	58.0	84.1	0.70 (95%CI NR)
Eyileten, 2021 [53]	34778397	CO	CV-death	NR			0.78 (0.61–0.95)
Luciani, 2023 [45]	36593094	CO	cardio-vascular death (5 y)	NR			0.70 (0.65–0.64)
Hsu, 2022 [98]	36006188	CS	predict peripheral arterial disease	0.62 µM (46.8 µg/L^c^)	39.7	84.3	0.63 (0.55–0.71)
Yang, 2025 [93]	39901484	CS	between peripheral arterial disease (PAD) and non-PAD	0.22 µM (18.6 µg/L^c^)	68.2	89.5	0.87 (0.78–0.93)
Aldujeli, 2024 [95]	39316928	CO	TMAO at primary post-percutaneous coronary intervention to detect coronary microvascular dysfunction at 3 mo.	NR			0.55 (0.46–0.64)
Aldujeli, 2024 [95]	39316928	CO	TMAO at 3 mo. post-percutaneous coronary intervention to detect coronary microvascular dysfunction at 3 mo.	3.9 µM	75.0	82.0	0.80 (0.73–0.87)
Spasova, 2024 [72]	38669041	CS?	carotid artery plaques (CAPs) grade III	2.8 µM (212 µg/L^c^)	70.2	73.9	0.75 (0.66–0.85)
Guo, 2020 [79]	32855821	CC	discriminate between coronary artery disease and controls	NR			0.60 (0.52–0.69)
Guo, 2020 [79]	32855821	CC	severe artery stenosis from mild	NR			0.62 (0.52–0.72)
Chou, 2019 [106]	30862856	CS	plaque rupture	2.0 µM	88.3	76.8	0.89 (0.84–0.94)
***All cause mortality***							
Zhang, 2021 [101]	33647820	CO	all cause death	7.8 µM	80.9	0.72	0.80 (0.74–0.87)
Eyileten, 2021 [53]	34778397	CO	all cause death	3.5 µM	61	75	0.68 (0.57–0.79)
Luciani, 2023 [45]	36593094	CO	all cause death (5 y)	NR			0.68 (0.64–0.72)
Konieczny, 2022 [99]	35970148	CO	identify people with CVD at risk for death at 5 y	5.9 µM	48.0	80.0	0.67 (0.62–0.71)
***Heart failure /arterial fibrillation***							
Guo, 2020 [108]	32859154	CC	heart failure with preserved ejection fraction and controls	7.1 µM			0.63 (95%CI NR)
Dong, 2020 [102]	32921087	CC	HF groups (NYAHAII, III, IV) and controls	NR			0.857 (0.67–1.00)
Dong, 2020 [102]	32921087	CC	HF groups (III) and controls	NR			0.85 (0.78–0.91)
Dong, 2020 [102]	32921087	CC	HF groups (IV) and controls	NR			0.91 (0.87–0.96)
Meng, 2024 [94]	39732662	CO	predicting recurrent atrial fibrillation	4.3 µM	65.5	93.5	0.84 (0.75–0.92)
***Renal outcome***							
Yu, 2024 [89]	38267025	CO	predict ESKD or doubling of serum creatinine at 1 y	NR			0.95 (95%CI NR)
Yu, 2024 [89]	38267025	CO	predict ESKD or doubling of serum creatinine at 3y	NR			0.82 (95%CI NR)
Yu, 2024 [89]	38267025	CO	predict ESKD or doubling of serum creatinine at 5 y	NR			0.77 (95%CI NR)
Guo, 2020 [108]	32859154	CC	eGFR < 60	8.5 µM			0.67 (95%CI NR)

^a^ Sensitivity (Sn%) and specificity (Sp%) of TMAO blood test to detect the corresponding outcomes as reported on the study level

^b^ The cutoff values that correspond to the sensitivity and specificity in the primary study. The cutoff values were used to define a threshold of TMAO that may be associated with the indicated outcomes. Cutoff values in brackets are the original ones reported in the primary studies. Alle units were converted into µM

^c^ Conversion factor: 1 µmol/L TMAO = 75.1 µg/L (or ng/ml)

CC, case-control; CHD, coronary heart disease; CO, cohort; CS, cross sectional, CVD, cardio-vascular disease; HF, heart failure; eGFR, estimated glomerular filtration rate; ESKD, end stage kidney disease; MACE, major advanced cardio-vascular and cerebral events; NR, not reported; PAD, peripheral arterial disease;T2DM, type 2 diabetes mellitus

The publications are sorted by the outcome. Some publications reported multiple outcomes and are therefore listed more than once

### TMAO concentrations and other outcomes

We combined non-overlapping cohort studies on the association between TMAO and all-cause mortality (*n* = 42), renal dysfunction (*n* = 7), heart failure (*n* = 8), and composite heterogeneous outcomes as defined on the study level (*n* = 20) (studies are described in Supplemental Tables 8 and 9). The results of the corresponding meta-analyses are described in details in Supplemental Data File 1. Both the magnitude and direction of the associations between TMAO concentrations and each of the investigated outcomes were similar to those between TMAO and MACE. The pooled HR (95%CIs) were small after correction for publication bias and the PIs included 1.00 in all meta-analyses [TMAO and all-cause mortality 1.08 (1.01–1.15), PI = 0.86–1.59, *n* = 42; kidney dysfunction 1.24 (1.03–1.51), PI = 0.70–2.22, *n* = 7; and heart failure 1.10 (1.00–1.21), PI = 0.88–1.37, *n* = 8].

## Discussion

There is significant uncertainty regarding the strength and nature of the association between TMAO and disease conditions in observational studies. This systematic review and meta-analysis of primary studies revealed a consistent evidence of systematic overestimation of the association between TMAO and several disease outcomes. Publication bias, confounding, high risk of bias and large heterogeneity between studies explained at least part of the observed associations (Supplemental Table 10).

Meta-analysis of cohort studies showed a weak association between higher plasma TMAO and the risk of MACE [HR (95%CIs) = 1.20 (1.12–1.27)], while the PI (0.93–1.54) indicated that this association may not hold true for all future studies of exchangeable populations. The association was further attenuated after correction for publication bias. To the best of our knowledge, the present meta-analysis included the largest number of independent publications and showed the lowest pooled estimate compared to previous meta-analyses [109–111] (Supplemental Table 11). Higher estimate from previous meta-analyses may be due to using unadjusted effect sizes, heterogeneous definitions of MACE, or high risk of bias in the primary studies. Moreover, we observed marked over-reporting of studies that were likely to find positive associations, while publication bias analyses suggested that a number of studies with negative results maybe unpublished. Overall, our study showed that the magnitude of the association between blood TMAO concentrations and MACE is small.

Plasma concentrations of TMAO are influenced by age, sex, and diseases that confound the association between TMAO and MACE [6, 7]. Therefore, elevated plasma concentrations of TMAO in people at risk for MACE may be a proxy marker for hypertension and renal dysfunction rather than TMAO itself causing the disease. The association between TMAO and MACE was stronger in studies from Asia/China compared to elsewhere. It could be argued that elevated TMAO is a risk factor for MACE in Asians due to different gut bacteria, diet, genetic factors or a mix of these factors. However, a stronger associations in Asian studies is likely to be due to methodological issues such as selection of the population into the studies (high prevalence of co-morbidities) or measuring blood concentrations of TMAO shortly after an acute event. TMAO concentrations are higher in the acute phase after stroke or myocardial infarction compared to concentrations measured later [37, 82].

Translation of a new biomarker into clinical practice requires valid and reproducible laboratory methods. We found that in-house liquid chromatography tandem mass spectrometry (LC-MS/MS) methods were the most used assays to measure TMAO in the literature. Few studies used ELISA [95, 112] or NMR methods [113]. Data on the assay conditions (e.g., column, mobile phase, or pH), internal validity (precision, linearity, recovery), and external validity (e.g., comparison between labs) are lacking. TMAO concentrations may be influenced by pre-analytical factors such as fasting [114], sample storage conditions, repeated thawing and freezing, and the time point of sample collection with regard to an acute event [82]. The heterogeneous analytical and prenalytical conditions across the studies may explain the lack of agreement on cutoff values to define elevated TMAO [89, 115, 116]. Harmonisation of TMAO assays between different laboratories need to be improved in future studies.

Complex interactions between diet, gut bacteria, liver metabolism and renal excretion affect TMAO and may confound its associations with diseases. Unlike TMAO, elevated high-sensetive C-reactive protein (hsCRP) shows an underlying vascular inflammatory process that is causally related to MACE, while adapting healthy lifestyle (e.g., weight reduction, physical activity, cessation of smoking) or drug interventions (e.g., statins) may lower hsCRP and reduce the risk of MACE [117]. Whether lowering plasma TMAO may show protective health effects remains speculative. Potential ways to lower TMAO are; lowering trimethylamine by altering gut bacteria, lowering dietary precursors of TMAO or inhibiting liver flavin-containing monooxygenase 3 (FMO3) that converts trimethylamine to TMAO. Interventions that may alter gut bacteria such as probiotics, prebiotics or synbiotics have failed to lower plasma TMAO or to ameliorate MACE risk factors [118–120]. Limiting dietary intake of choline or betaine is not recommended since these nutrients are needed for normal liver function, one-carbon metabolism, brain development and cognition. Currently there are no interventions to inhibit FMO3 in humans.

A fundamental part of using observational data for causal inference is adjustment for confounders that are selected apriori based on existing knowledge, but not on the results [8]. Roughly half of the literature on TMAO showed conflation of causality and prediction methods. While in prediction studies, the predictors are included in a multivariate model to enable disease prognosis or diagnosis irrespective of the role of these variables in the ethiology of the dsease [8]. In studies that aimed at investigating a causal link between TMAO and diseases [example; [9, 23], the rational for selecting the confounders was not explained or the adjustments were performed in crude categories of the confounders (e.g., categories of age, BMI, or medications), suggesting residual confounding (e.g., from the diet, gut microbiome, antibiotic use, liver function, or medications). Therefore, studies on TMAO should use appropriate and transparent data analyses to address either causality or prediction.

The present study may have limitations. For example we excluded studies published in languages other than English which may have caused publication bias. However, we found evidence that all estimated “missing” studies are on the left side of the distribution curve, i.e., negative studies. In addition, the pooled estimates from Chinese studies were generally higher than other studies, suggesting that if we would include studies published in Chinese, the publication bias may even be more serious. Moreover, the data available did not enable in-depth analyses of the effect of the clinical characteristics of the participants on heterogeneity. Instead, analytical methods and time point of blood collection may be of importance but were not consistently reported enough to be investigated. Finally, we defined MACE component diseases a priori, thus studies that did not fulfill this definition (e.g., due to including all-cause mortality in MACE) were analysed separately. However, sensitivity analyses combining widely used heterogeneous definitions of MACE gave similar results.

## Conclusion

We found that the previously reported association between TMAO and MACE may be overestimated due to systematic bias of multiple sources. Residual associations after correcting for publication bias and confounding are small in magnitude. Plasma TMAO was often measured shortly after an acute vascular event and was measured in people who were anyway at risk of the outcome due to comorbid conditions (e.g., renal dysfunction or hypertension). Moreover, no conclusion can be made about a disease-relevant threshold to define elevated TMAO concentrations, limiting its potential use as a single prognostic biomarker for MACE risk. Future studies may test whether TMAO may have incremental prognostic value when combined with established cardiovascular risk models. Studies among healthy people and studies to investigate the health effect of lowering TMAO are still lacking.

## Supplementary Information

Below is the link to the electronic supplementary material.

Supplementary Material 1

Supplementary Material 2

Supplementary Material 3

Supplementary Material 4

## Data Availability

All data generated or analysed during this study are included in this published article [and its supplementary information files].

## Abbreviations

AUC: Area under the curve
CHS: Cardiovascular Health Study
CNSR-III: The third China National Stroke Registry\
CV: Cardiovascular
HF: Heart failure
FMNO3: Flavin-containing monooxygenase 3
MACE: Major adverse cardiac and cerebrovascular events
MESA: The Multi-Ethnic Study of Atherosclerosis study
NPV: Negative predictive value
PI: Prediction interval
PMID: Unique identifier number in MEDLINE
PPV: Positive predictive value
PREDIMED: The Prevention With Mediterranean Diet trial
PREVEND: Prevention of REnal and Vascular ENd-stage Disease study
PROSPERO: International Prospective Register of Systematic Reviews
OHAT: Office of Health AssessMt and Translation
ROB: Risk of bias
ROC: Receiver operating characteristic
TMAO: Trimethylamine N-oxide
