# Supplementary material for: Trimethylamine N-oxide and major adverse cardiac and cerebrovascular events: results of umbrella review and meta-analysis
Source: Nutr Metab (Lond). 2026 Sep 23;23:108. doi: 10.1186/s12986-026-01214-0 (PMC13599251; doi:10.1186/s12986-026-01214-0)
Supplement: Supplementary file 1 [file 12986_2026_1214_MOESM2_ESM.docx]

**Supplementary Material (2)**

**Quantitative data analyses on additional outcomes**

Trimethylamine N-oxide and major adverse cardiac and cerebrovascular events: Results of umbrella review and meta-analysis

**Table of content:**

1- TMAO and all-cause mortality

2- TMAO and renal outcomes

3- TMAO, heart failure and arterial fibrillation

4- TMAO and MACE as reported on the study level

5- TMAO and severity of MACE in cross-sectional studies

*Figures*: Figures S1 to S10

*Tables:* Tables S1 and S2

**1- Association between TMAO and all-cause mortality**

1.1- **TMAO and all-cause mortality in cohort studies**

There were 47 publications with unique PMIDs that investigated the association between TMAO concentrations and all-cause mortality as a unique outcome and had presented adjusted HR (95%CIs) values. Publications from the Cleveland Clinic were assumed to overlap (n = 6, PMIDs; 28077467, 25599331, 27792653, 27287696, 27864387, and 25444145) and they were merged as one study before running the meta-analysis. Li et al., published two studies in 2022 with very similar populations that were recruited from the same hospital within the same time window (PMIDs; 35686339 and 36354779) [1;2]. Two publications from the SURDIAGENE cohorts reported all-cause mortality and were combined as one study [3;4] [PMIDs; 32301490 and 35681209]. These two studies were combined as one study.

The final meta-analysis on the association between TMAO and all-cause mortality included 42 non-overlapping source populations. The pooled HR (95%CIs) for all-cause mortality was 1.17 (1.11-1.25); and 1.17 (1.09-1.27) after Knapp-Hartung adjustment, PI = 0.86-1.59 (**Figure S1**). The Q-value was 191.5, df = 41, p <0.001 and I^2^ = 78.6%.

The Egger’s test supports the presence of considerable publication bias [Intercept (95%CIs) = 1.77 (0.81-2.73), t = 3.73, p = 0.0006]. The Funnel plot and Duval and Tweedie’s trim and fill test suggested that 12 studies all on the left side of the distribution were missing (**Figure S2**). Accounting for unpublished studies attenuated the association; pooled HR (95%CIs) = 1.08 (1.01-1.15) and 1.08 (0.98-1.18) after Knapp-Hartung adjustment.

1.2- **Sensitivity analyses of the association between TMAO and all-cause mortality in cohort studies**

The leave-one-out meta-analyses showed that there were no outliers that would substantially affect the strength or the direction of the pool estimate (data not shown).

Meta-analysis of the adjusted HR and 95%CI from 6 publications conducted at the Cleveland Clinic (PMIDs; 28077467, 25599331, 27792653, 27287696, 27864387, and 25444145) showed a pooled HR (95%CIs) estimate of 1.40 (1.25-1.55). While, the remaining 41 studies (i.e., excluding 6 publications from the Cleveland Clinic) showed lower pooled HR (95%CIs) = 1.16 (1.10-1.23); and 1.16 (1.08-1.26) after Knapp-Hartung adjustment, Q-value = 169.7, df = 40, p <0.001, and I^2^ = 76.4%. Thus, the contribution of the pooled estimates from the Cleveland Clinic (here considered as a single study) may have a small effect on overestimating the associations because the relative weight of the Cleveland Clinic estimate to the overall pooled HR was only 3.6%.

Additionally, we conducted a meta-analysis using the unadjusted HR and 95%CI to estimate how adjustment for covariates on the study level may influence the pooled estimate. There were 38 studies reporting unadjusted HR (4 publications did not report unadjusted HR [5-8]). The pooled unadjusted HR (95%CIs) = 1.53 (1.39-1.69); and 1.53 (1.34-1.74) after Knapp-Hartung adjustment, PI = 0.89-2.64, Q-value = 504.4, I^2^ = 92.7% (**Figure S3**). This estimate is considerably lower than the pooled estimate of the confounder-adjusted effect size was 1.17 (1.11-1.25).

Egger’s test and funnel plot suggested significant publication bias in studies showing unadjusted HR (p = 0.0196) and 11 studies maybe missing (**Figure S4**). After correction for publication bias, the pooled HR (95%CIs) of the unadjusted effect size were 1.31 (1.19-1.45) and 1.31 (1.13-1.47) after Knapp-Hartung adjustment. Compared to the pooled estimate after correction for both confounding and publication bias [1.08 (1.01-1.15)] (Supplemental Figure 11), the estimate of the unadjusted HR after correction for publication bias [1.31 (1.19-1.45)] is considerably higher (no overlap in the 95%CIs). Therefore, using unadjusted effect size in previous meta-analyses may have overestimated the association between TMAO and all-cause mortality.

Taken together, due to the large number of studies included in the present meta-analysis, the pooled HR estimate was rather robust (i.e., not liable to changes due to a single study) and precise (i.e., low confidence intervals). However, publication bias, confounding and overlapping studies may cause a considerable shift in the association estimate (overestimation) of a similar magnitude as we have seen for the association between TMAO and MACE. Given all these sources of bias, heterogeneity and confounding the residual association between TMAO and all-cause mortality is weak and may be explained by residual confounding or clinical characteristics of the generally multi-morbid populations (e.g., background diseases, age, sex, medications).

1.3- **Subgroup analyses of cohort studies on the association between TMAO and all-cause mortality**

Subgroup analyses on all-cause mortality are shown in **Table S1**. Studies with higher overall ROB, selection bias, attrition, and higher ROB in detecting the outcome estimated larger magnitude associations between TMAO and all-cause mortality. Moreover, the association was stronger in publications from China [1.34 (1.01-1.77), n = 7 publications] compared to publications from other parts of the world combined [1.18 (1.11-1.26), n = 35 publications]. Subgroup analyses by elements related to study design or quality did not explain high between-study heterogeneity which could be due to other factors such as clinical characteristics of the participants.

| **Table S1.** Subgroup analyses on the association between circulating TMAO concentrations and **all-cause mortality in cohort studies**. | | | |
| --- | --- | --- | --- |
| **Subgroups** | **Pooled HR (95%CIs)** | **N** | **I^2^ (heterogeneity)** |
| **Overall risk of bias** |  |  |  |
| Low (up to three domains have high ROB) | 1.18 (1.09-1.29) | 20 | 71.9% |
| High (≥ 4 domains have high ROB) | 1.22 (1.11-1.34) | 24 | 82.9% |
| **Selection bias** |  |  |  |
| Low | 1.11 (1.03-1.20) | 13 | 64.5% |
| High | 1.25 (1.15-1.37) | 29 | 83.8% |
| **Confounding** |  |  |  |
| Low | 1.19 (1.09-1.30) | 27 | 74.9% |
| High | 1.22 (1.11-1.34) | 17 | 84.1% |
| **Attrition** |  |  |  |
| Low | 1.13 (1.06-1.21) | 17 | 68.7% |
| High | 1.25 (1.13-1.40) | 25 | 84.0% |
| **Detection bias exposure** |  |  |  |
| Low | 1.21 (1.12-1.31) | 25 | 78.2% |
| High | 1.18 (1.06-1.32) | 18 | 82.3% |
| **Detection bias outcome** |  |  |  |
| Low | 1.14 (1.06-1.22) | 25 | 73.9% |
| High | 1.32 (1.16-1.50) | 19 | 84.1% |
| **Selective reporting** |  |  |  |
| Low | 1.21 (1.11-1.32) | 31 | 74.4% |
| High | 1.19 (1.08-1.31) | 12 | 87.1% |
| **Other sources of bias** |  |  |  |
| Low | 1.11 (1.02-1.20) | 12 | 57.5% |
| High | 1.24 (1.14-1.35) | 31 | 83.4% |
| **Adjust for renal function** |  |  |  |
| No | 1.02 (0.91-1.16) | 6 | 55.3% |
| Yes | 1.23 (1.14-1.32) | 36 | 80.1% |
| **Adjust for hypertension** |  |  |  |
| No | 1.14 (0.97-1.35) | 10 | 82.6% |
| Yes | 1.21 (1.13-1.30) | 32 | 78.0% |
| **Publication years** |  |  |  |
| 2017-2020 | 1.25 (1.13-1.39) | 21 | 79.8% |
| 2021-2024 | 1.12 (1.05-1.21) | 21 | 71.7% |
| **Follow up duration** |  |  |  |
| Less than 5 years | 1.21 (1.08-1.35) | 21 | 75.3% |
| ≥ 5 years | 1.19 (1.10-1.29) | 22 | 81.9% |
| **By country of participants** |  |  |  |
| USA and Canada | 1.14 (1.02-1.28) | 10 | 85.2% |
| Nordic countries | 1.03 (0.96-1.10) | 7 | 42.5% |
| China | 1.34 (1.01-1.77) | 7 | 84.5% |
| Europe excluding Nordic countries | 1.24 (1.11-1.38) | 15 | 70.8% |
| Asia | 3.35 (1.57-7.16) | 2 | - |
| New Zealand | 2.00 (1.12-3.57) | 1 | - |
| Europe including Nordic countries | 1.18 (1.09-1.28) | 22 | 77.3% |
| All countries combined, except China | 1.18 (1.11-1.26) | 35 | 79.7% |
| All cohort studies reported the adjusted HR (95%CIs). Meta-analyses were conducted using random effects model. | | | |

1.4- **Dose-response pattern analysis between TMAO cutoff values and logHR for all-cause mortality**

We tested the hypothesis that the magnitude of a given TMAO cutoff is positively associated with the logHR magnitude. A positive correlation would suggest a dose-response pattern between TMAO levels and the risk for all-cause mortality. No association was found between the cutoff values that were used to define elevated TMAO concentrations and the logHR for all-cause mortality among 78 sub-studies that reported explicit cutoff values for TMAO (**Figure S5**).

Only 2 publications with a case-control study design investigated the association between TMAO concentrations and all-cause mortality (n = 2). No meta-analysis was conducted.

**2- TMAO and renal outcomes**

Nine publications with a longitudinal study design reported on the association between TMAO and renal dysfunction. The definition of adverse renal outcomes is not standardized. Definitions of the end points as reported in the literature are shown in **Table S2**.

**Table S2**. Definition of renal outcomes on the study level in cohort studies.

| **Citation** | **PMID** | **Objective** | | **Renal outcome definition** |
| --- | --- | --- | --- | --- |
| **Included in the meta-analysis** | | | | |
| Winther SA, 2019 [9] | 31123156 | Study the additive prognostic value of TMAO and incident CVD, renal complications, and mortality, in patients with type 1 diabetes | - ESRD; defined as CKD stage 5 (ICD–10 code N18.5), initiation of permanent dialysis, renal transplantation, eGFR<15mL/min/ 1.73m^2^, or mortality from chronic renal failure. - Macroalbuminuria–ESRD. | |
| Winther SA, 2021 [10] | 33657115 | Associations between TMAO and its precursors and risk of mortality, CVD and renal function decline in individuals with T2D and microalbuminuria | eGFR decline by ≥ 30% from baseline | |
| Sapa H, 2022 [11] | 35351578 | Association of plasma TMAO with CV mortality, all–cause mortality and incident KFRT in people with diabetes and baseline eGFR < 60 ml/min/1.73 m^2^ | KFRT = transfer to dialysis | |
| Cheng E, 2025 [12] | 39709651 | Associations of plasma TMAO and related metabolites with eGFR decline and the progression of kidney disease among patients with stage 3–5 CKD | - Rapid decline in kidney function, defined as an annualized decline in the eGFR of >3 ml/min/ 1.73 m^2^ from baseline to the first year of follow–up; - Renal composite outcome: a composite of progression of kidney disease, defined as a sustained 40 % decline in eGFR from baseline, initiation of long–term dialysis therapy, preemptive kidney transplantation, or renal mortality | |
| Wang M, 2024 [13] | 38593157 | Association between TMAO and incident and progression of CKD | - Incident CKD defined as both a eGFR decline by ≥ 30% from baseline and resulting GFR < 60 ml/min per 1.73 m^2^; - Development of CKD stage 3 or above defined by an algorithm: based on hospitalization ICD codes. | |
| Yu PS, 2024 [14] | 38267025 | Association between TMAO and adverse kidney outcomes and all–cause mortality in patients with T2D | ESKD or doubling of serum creatinine; doubling of serum creatinine; and 30% reduction of eGFR within 2 years | |
| Dai L, 2022 [15] | 36166845 | Association of TMAO and all-cause mortality and kidney replacement therapy in elderly people ≥ 65 y | Kidney replacement therapy initiation during 48 months of follow–up | |
| **Not included in the meta-analysis** | | | | |
| Robinson-Cohen C, 2016 [8] | 27513517 | Study FMO3 genetic variation and TMAO in relation to disease progression and all–cause mortality in CKD patients | The primary renal outcome was the relative annualized change in kidney function, defined by the exponentiated slope of log–transformed eGFR values | |
| Meyer KA, 2016 [16] | 27792658 | Quantified prospective associations between TMAO and 10–year (1) CAC incidence and (2) CAC progression. | - 10 years change in eGFR. - 10 years change in urine albumin/creatinine ratio. | |

Two studies were not included in the quantitative data analyses because they reported statistical measures other than HR (95%CIs) [8;16]. The meta-analysis included 7 publications with unique PMIDs on the prospective association between plasma concentrations of TMAO and the HR of renal outcomes [9-15]. The pooled HR (95%CIs) = 1.24 (1.03-1.51) and 1.24 (0.95-1.63) after Knapp-Hartung adjustment, PI = 0.70-2.22. The Q-statistics showed high heterogeneity across the studies (Q = 23.9, df = 6, p <0.001, I^2^ = 74.9%) (**Figure S6**).

Egger’s test did not suggest publication bias (p = 0.906), though less than 10 studies included (**Figure S7**). To avoid overinterpretation, subgroup analyses were not conducted due to low number of studies in the subgroups. However, it is noteworthy that the pooled estimates for MACE, all-cause death, and renal outcomes were similar in the direction and magnitude, suggesting that the statistical associations are unspecific towards the outcome.

**3- TMAO, heart failure and arterial fibrillation**

We identified 9 publications with unique PMIDs and a cohort study design on the association between plasma concentrations of TMAO and heart failure including mortality due to heart failure [1;2;4;9;17-21]. Two studies included overlapping populations [2;22]. The pooled HR (95%CIs) from 8 non-overlapping studies was 1.10 (1.00-1.21) and 1.10 (0.96-1.27) after Knapp-Hartung adjustment, PI = 0.88-1.37, Q-value = 11.1, df = 7, p = 0.133, and I^2^ = 37.2% (**Figure S8-A**). Both Egger’s test (p = 0.256) and the funnel plot did not suggest significant publication bias (**Figure S8-B**).

Meta-analysis of 4 publications with a case-control design on the association between TMAO and heart failure [23-26] showed a non-significant pooled OR (95%CIs) = 2.43 (0.83-7.06), the Knapp-Hartung adjusted estimate = 2.43 (0.66-8.95), I^2^ = 86.9%. Publication bias was not evaluated due to the low number of publications (< 10).

Two independent publications were identified on the outcome arterial fibrillation as a unique outcome [27;28]. No meta-analysis was conducted.

**4- TMAO and composite outcomes as reported on the study level**

We identified 23 publications with unique PMIDs reporting composite outcomes including heterogeneous definitions and etiologies such as all-cause mortality plus MACE, all-cause mortality plus heart failure and all-cause mortality plus arterial fibrillation. Most of these publications reported additional outcomes that were included in the data analyses elsewhere in this paper.

We first checked for potential overlap between populations from different publications. The overlapping studies were pooled before combining them into a meta-analysis. For example, a publication by Tang et al., (2017) included people with type 2 diabetes from the GeneBank study [29]. A second publication by Tang et al., (2015) included people with chronic heart failure who were recruited within the same time window [30]. In a third publication, Tang et al., (2013) included people from the GenBank study undergoing cardiac catheterization not in the setting of acute coronary syndrome [31]. We also identified 2 publications based on the Swiss SPUM cohort reporting composite outcomes [32;33]. Moreover, Li et al., published two studies in 2020, both included Chinese population recruited in the same time window (2017 to 2020) and are very similar in their characteristics [1;2].

The meta-analysis of the association between TMAO and composite outcomes included 20 non-overlapping publications with prospective study design reporting adjusted HR (95%CIs). The pooled HR (95%CIs) was 1.23 (1.15-1.32), and 1.23 (1.14-1.33) after Knapp-Hartung adjustment, PI = 0.96-1.59, Q-value = 49.2, df = 19, p < 0.0001 and I^2^ = 61.4% (**Figure S9**). Egger’s test was significant suggesting publication bias (p = 0.005). After correcting for 6 missing publications, the HR (95%CIs) were attenuated 1.17 (1.09-1.26), the Knapp-Hartung adjusted estimate = 1.17 (1.07-1.28). In a sensitivity analysis, excluding one study each time did not influence the direction or the strength of the association (data not shown).

**5- TMAO and severity of MACE in cross-sectional studies**

Twenty-four publications with a cross sectional design were identified; 8 were excluded due to incompatible effect size; 1 due to implausible effect size; and 1 because of irrelevant outcome (**Supplemental Table 4, Excel**). Three of the 14 cross-sectional candidate studies were on MACE [34-36] [pooled OR (95%CIs) = 1.49 (95%CI: 1.06-2.08)]. Whereas, 11 were on clinical severity of MACE defined using heterogeneous criteria (**Table S2**). Meta-analysis of the 11 publications with unique PMIDs and a cross sectional study design on MACE-severity showed a pooled OR (95%CIs) estimate of 1.26 (1.17-1.35) and 1.26 (0.93-1.70) after Knapp-Hartung adjustment, PI = 1.03-1.53, Q-value = 238.2, df (Q) = 10, p < 0.0001, I^2^ = 95.8% (**Figure S10**). The Egger’s test was significant (p = 0.005), suggesting publication bias. After correcting for 6 missing studies, the pooled effect size was not statistically significant [pooled OR (95%CIs) = 1.05 (0.97-1.13) and 1.05 (0.73-1.50) after Knapp-Hartung adjustment]. Only 2 cross sectional studies had a low overall ROB. Subgroup analyses were not performed due to low number of studies in the subgroups.

Reference List

1.	Li N, Wang Y, Zhou J, Chen R, Li J, Zhao X, Zhou P, Liu C, Chen Y, Song L et al. (2022) Association between the Changes in Trimethylamine N-Oxide-Related Metabolites and Prognosis of Patients with Acute Myocardial Infarction: A Prospective Study. J Cardiovasc Dev Dis 9.

2.	Li N, Zhou J, Wang Y, Chen R, Li J, Zhao X, Zhou P, Liu C, Song L, Liao Z et al. (2022) Association between trimethylamine N-oxide and prognosis of patients with acute myocardial infarction and heart failure. ESC Heart Fail 9:3846-3857.

3.	Croyal M, Saulnier PJ, Aguesse A, Gand E, Ragot S, Roussel R, Halimi JM, Ducrocq G, Cariou B, Montaigne D et al. (2020) Plasma Trimethylamine N-Oxide and Risk of Cardiovascular Events in Patients With Type 2 Diabetes. J Clin Endocrinol Metab 105.

4.	Wargny M, Croyal M, Ragot S, Gand E, Jacobi D, Trochu JN, Prieur X, Le MC, Goronflot T, Cariou B et al. (2022) Nutritional biomarkers and heart failure requiring hospitalization in patients with type 2 diabetes: the SURDIAGENE cohort. Cardiovasc Diabetol 21:101.

5.	Israr MZ, Bernieh D, Salzano A, Cassambai S, Yazaki Y, Heaney LM, Jones DJL, Ng LL, Suzuki T (2021) Association of gut-related metabolites with outcome in acute heart failure. Am Heart J 234:71-80.

6.	Schuett K, Kleber ME, Scharnagl H, Lorkowski S, Marz W, Niessner A, Marx N, Meinitzer A (2017) Trimethylamine-N-oxide and Heart Failure With Reduced Versus Preserved Ejection Fraction. J Am Coll Cardiol 70:3202-3204.

7.	Hernandez L, Ward LJ, Arefin S, Ebert T, Laucyte-Cibulskiene A, Heimburger O, Barany P, Wennberg L, Stenvinkel P, Kublickiene K (2022) Blood-brain barrier and gut barrier dysfunction in chronic kidney disease with a focus on circulating biomarkers and tight junction proteins. Sci Rep 12:4414.

8.	Robinson-Cohen C, Newitt R, Shen DD, Rettie AE, Kestenbaum BR, Himmelfarb J, Yeung CK (2016) Association of FMO3 Variants and Trimethylamine N-Oxide Concentration, Disease Progression, and Mortality in CKD Patients. PLoS ONE 11:e0161074.

9.	Winther SA, Ollgaard JC, Tofte N, Tarnow L, Wang Z, Ahluwalia TS, Jorsal A, Theilade S, Parving HH, Hansen TW et al. (2019) Utility of Plasma Concentration of Trimethylamine N-Oxide in Predicting Cardiovascular and Renal Complications in Individuals With Type 1 Diabetes. Diabetes Care 42:1512-1520.

10.	Winther SA, Ollgaard JC, Hansen TW, von Scholten BJ, Reinhard H, Ahluwalia TS, Wang Z, Gaede P, Parving HH, Hazen S et al. (2021) Plasma trimethylamine N-oxide and its metabolic precursors and risk of mortality, cardiovascular and renal disease in individuals with type 2-diabetes and albuminuria. PLoS ONE 16:e0244402.

11.	Sapa H, Gutierrez OM, Shlipak MG, Katz R, Ix JH, Sarnak MJ, Cushman M, Rhee EP, Kimmel PL, Vasan RS et al. (2022) Association of Uremic Solutes With Cardiovascular Death in Diabetic Kidney Disease. Am J Kidney Dis 80:502-512.

12.	Cheng E, Hung SC, Lin TY (2025) Association of trimethylamine N-oxide and metabolites with kidney function decline in patients with chronic kidney disease. Clin Nutr 44:239-247.

13.	Wang M, Tang WHW, Li XS, de Oliveira Otto MC, Lee Y, Lemaitre RN, Fretts A, Nemet I, Sotoodehnia N, Sitlani CM et al. (2024) The Gut Microbial Metabolite Trimethylamine N -oxide, Incident CKD, and Kidney Function Decline. J Am Soc Nephrol 35:749-760.

14.	Yu PS, Wu PH, Hung WW, Lin MY, Zhen YY, Hung WC, Chang JM, Tsai JR, Chiu YW, Hwang SJ et al. (2024) Association Between Trimethylamine N-oxide and Adverse Kidney Outcomes and Overall Mortality in Type 2 Diabetes Mellitus. J Clin Endocrinol Metab 109:2097-2105.

15.	Dai L, Massy ZA, Stenvinkel P, Chesnaye NC, Larabi IA, Alvarez JC, Caskey FJ, Torino C, Porto G, Szymczak M et al. (2022) The association between TMAO, CMPF, and clinical outcomes in advanced chronic kidney disease: results from the European QUALity (EQUAL) Study. Am J Clin Nutr 116:1842-1851.

16.	Meyer KA, Benton TZ, Bennett BJ, Jacobs DR, Jr., Lloyd-Jones DM, Gross MD, Carr JJ, Gordon-Larsen P, Zeisel SH (2016) Microbiota-Dependent Metabolite Trimethylamine N-Oxide and Coronary Artery Calcium in the Coronary Artery Risk Development in Young Adults Study (CARDIA). J Am Heart Assoc 5.

17.	Tang WHW, Lemaitre RN, Jensen PN, Wang M, Wang Z, Li XS, Nemet I, Lee Y, Lai HTM, de Oliveira Otto MC et al. (2024) Trimethylamine N-Oxide and Related Gut Microbe-Derived Metabolites and Incident Heart Failure Development in Community-Based Populations. Circ Heart Fail 17:e011569.

18.	Zhao S, Tian Y, Wang S, Yang F, Xu J, Qin Z, Liu X, Cao M, Zhao P, Zhang G et al. (2023) Prognostic value of gut microbiota-derived metabolites in patients with ST-segment elevation myocardial infarction. Am J Clin Nutr 117:499-508.

19.	Kinugasa Y, Nakamura K, Kamitani H, Hirai M, Yanagihara K, Kato M, Yamamoto K (2021) Trimethylamine N-oxide and outcomes in patients hospitalized with acute heart failure and preserved ejection fraction. ESC Heart Fail 8:2103-2110.

20.	Suzuki T, Heaney LM, Bhandari SS, Jones DJ, Ng LL (2016) Trimethylamine N-oxide and prognosis in acute heart failure. Heart 102:841-848.

21.	Lever M, George PM, Slow S, Bellamy D, Young JM, Ho M, McEntyre CJ, Elmslie JL, Atkinson W, Molyneux SL et al. (2014) Betaine and Trimethylamine-N-Oxide as Predictors of Cardiovascular Outcomes Show Different Patterns in Diabetes Mellitus: An Observational Study. PLoS ONE 9:e114969.

22.	Li N, Wang Y, Zhou J, Chen R, Li J, Zhao X, Zhou P, Liu C, Chen Y, Song L et al. (2022) Association between the Changes in Trimethylamine N-Oxide-Related Metabolites and Prognosis of Patients with Acute Myocardial Infarction: A Prospective Study. J Cardiovasc Dev Dis 9.

23.	Papandreou C, Bullo M, Hernandez-Alonso P, Ruiz-Canela M, Li J, Guasch-Ferre M, Toledo E, Clish C, Corella D, Estruch R et al. (2021) Choline Metabolism and Risk of Atrial Fibrillation and Heart Failure in the PREDIMED Study. Clin Chem 67:288-297.

24.	Dong Z, Zheng S, Shen Z, Luo Y, Hai X (2021) Trimethylamine N-Oxide is Associated with Heart Failure Risk in Patients with Preserved Ejection Fraction. Lab Med 52:346-351.

25.	Dong Z, Liang Z, Wang X, Liu W, Zhao L, Wang S, Hai X, Yu K (2020) The correlation between plasma trimethylamine N-oxide level and heart failure classification in northern Chinese patients. Ann Palliat Med 9:2862-2871.

26.	Guo F, Qiu X, Zhu Y, Tan Z, Li Z, Ouyang D (2020) Association between plasma betaine levels and dysglycemia in patients with coronary artery disease. Biosci Rep 40.

27.	Meng S, Ni T, Du Q, Liu M, Ge P, Geng J, Wang B (2024) Pre-procedural TMAO as a predictor for recurrence of atrial fibrillation after catheter ablation. BMC Cardiovasc Disord 24:750.

28.	Svingen GFT, Zuo H, Ueland PM, Seifert R, Loland KH, Pedersen ER, Schuster PM, Karlsson T, Tell GS, Schartum-Hansen H et al. (2018) Increased plasma trimethylamine-N-oxide is associated with incident atrial fibrillation. Int J Cardiol 267:100-106.

29.	Tang WH, Wang Z, Li XS, Fan Y, Li DS, Wu Y, Hazen SL (2017) Increased Trimethylamine N-Oxide Portends High Mortality Risk Independent of Glycemic Control in Patients with Type 2 Diabetes Mellitus. Clin Chem 63:297-306.

30.	Tang WH, Wang Z, Shrestha K, Borowski AG, Wu Y, Troughton RW, Klein AL, Hazen SL (2015) Intestinal microbiota-dependent phosphatidylcholine metabolites, diastolic dysfunction, and adverse clinical outcomes in chronic systolic heart failure. J Card Fail 21:91-96.

31.	Tang WH, Wang Z, Levison BS, Koeth RA, Britt EB, Fu X, Wu Y, Hazen SL (2013) Intestinal microbial metabolism of phosphatidylcholine and cardiovascular risk. N Engl J Med 368:1575-1584.

32.	Davies A, Wenzl FA, Li XS, Winzap P, Obeid S, Klingenberg R, Mach F, Raber L, Muller O, Matter CM et al. (2023) Short and medium chain acylcarnitines as markers of outcome in diabetic and non-diabetic subjects with acute coronary syndromes. Int J Cardiol 389:131261.

33.	Li XS, Obeid S, Klingenberg R, Gencer B, Mach F, Raber L, Windecker S, Rodondi N, Nanchen D, Muller O et al. (2017) Gut microbiota-dependent trimethylamine N-oxide in acute coronary syndromes: a prognostic marker for incident cardiovascular events beyond traditional risk factors. Eur Heart J 38:814-824.

34.	Wang Z, Klipfell E, Bennett BJ, Koeth R, Levison BS, Dugar B, Feldstein AE, Britt EB, Fu X, Chung YM et al. (2011) Gut flora metabolism of phosphatidylcholine promotes cardiovascular disease. Nature 472:57-63.

35.	Yang HH, Chen YC, Liu CH, Hsu BG (2025) Serum Trimethylamine N-Oxide Levels as a Predictor of Peripheral Arterial Disease in Kidney Transplant Recipients. Med Sci Monit 31:e947197.

36.	Senthong V, Kiatchoosakun S, Wongvipaporn C, Phetcharaburanin J, Tatsanavivat P, Sritara P, Phrommintikul A (2021) Gut microbiota-generated metabolite, trimethylamine-N-oxide, and subclinical myocardial damage: a multicenter study from Thailand. Sci Rep 11:14963.
