# Supplementary material for: Trimethylamine N-oxide and major adverse cardiac and cerebrovascular events: results of umbrella review and meta-analysis
Source: Nutr Metab (Lond). 2026 Sep 23;23:108. doi: 10.1186/s12986-026-01214-0 (PMC13599251; doi:10.1186/s12986-026-01214-0)
Supplement: Supplementary file 2 [file 12986_2026_1214_MOESM3_ESM.docx]

**Supplementary Material (3)**

**Trimethylamine N-oxide and major adverse cardiac and cerebrovascular events: Results of umbrella review and meta-analysis**

**Supplemental Tables 1 to 11**

**Supplemental Table 1.** The study population, intervention, comparator, and outcomes (PICO).

**Supplemental Table 2.** Keywords used in the updated search.

**Supplemental Table 3.** Risk of Bias Rating tool (assessment of internal validity of the study).

**Supplemental Table 4**. List of excluded articles (n = 119) in the full text stage and reasons for exclusion.

**Supplemental Table 5.** Subgroup analyses of cohort studies by risk of bias (ROB) in individual studies on the association between TMAO concentrations and MACE.

**Supplemental Table 6.** Additional subgroup analyses according to factors related to design or conduct of individual cohort studies on the association between TMAO concentrations and MACE

**Supplemental Table 7.** Meta-analyses and subgroup analyses of the associations between TMAO and MACE from case-control studies reporting adjusted OR and 95%CI.

**Supplemental Table 8.** List of identified cohort and case-control studies on TMAO.

**Supplemental Table 9**. Cross-sectional studies that reported on the association between TMAO and severity of MACE.

**Supplemental Table 10.** Summary of common sources of systematic bias in the literature on TMAO and cardio-vascular diseases.

**Supplemental Table 11.** Comparison of results from the present meta-analysis with those of previous meta-analyses on the outcomes MACE and all-cause mortality.

**Supplemental Table 1.** The study Population, Intervention, Comparator, and Outcomes (PICO).

| **PICO Statement** | |
| --- | --- |
| **Population** | Humans: epidemiological studies including healthy people (men and women), people with risk factors for vascular diseases, people with impaired kidney function, people recruited after a vascular event, people at risk for any of the outcomes due to their previous morbidities such as people with diabetes, hyperlipidemia, hypertension, thrombosis, and people taking medications. |
| **Intervention (exposure)** | Elevated concentrations of TMAO in plasma (EDTA, or Heparin) or serum defined by any pre-defined or data driven cutoff (SD, tertiles, quartiles, quintiles). |
| **Comparator** | Concentrations of TMAO below the study given cutoff (or reference category) |
| **Outcomes** | major adverse cardiac and cerebrovascular events (MACE) as defined on the study level, cardiovascular diseases, myocardial infarction, stroke (ischemic and small vessel disease), stroke- or CVD-specific death, other cerebral diseases,  atrial fibrillation, heart insufficiency, renal diseases and kidney dysfunction,  and all-cause mortality. |

**Supplemental Table 2.** Keywords used in the updated search on 23-February-2025 to identify original publications in Medline.

|  | **Population** |
| --- | --- |
|  | keywords: Healthy people (men and women), people with risk factors for vascular diseases, people with stroke, people with impaired kidney function, people recruited after a vascular event, people at risk for any of these conditions due to their morbidities such as diabetes, hyperlipidemia, hypertension, thrombosis, people taking anti-coagulants |
| Search #1 | “human”[tiab] OR “adult*”[tiab] OR “people”[tiab] OR “patient*”[tiab] |
|  | **Intervention (exposure)** |
|  | keywords: Elevated concentrations of plasma or serum TMAO (any concentrations above the lowest category of the TMAO concentrations should be considered as elevated) |
| Search #2 | - “TMAO*”[tiab] OR “trimethylamine”[tiab] OR “level”[tiab] OR “elevated*”[tiab] OR “concentr*”[tiab] OR “concentration”[tiab] OR “trimethylamine N oxide”[tiab] OR “trimethylamine-N-oxide”[tiab] OR “gut bacteria*”[tiab] OR “metabolite*”[tiab] OR “choline*”[tiab] OR “trimethylammonium oxide”[tiab] OR “microbial metabolite”[tiab] OR “trimethylamine oxide”[tiab] OR “methyl-amin*”[tiab] OR “trimethylamin*”[tiab] OR “trimethyloxamine*”[tiab] OR "trimethyloxamine"[Supplementary Concept] AND "trimethyloxamine"[All Fields] OR "trimethylamine n oxide"[All Fields] OR "trimethylamine n-oxide"[All Fields] OR “Methylamines"[Mesh] OR “Gastrointestinal Microbiome*” [Mesh] |
|  | **Comparison** |
|  | Low concentrations of TMAO |
|  | **Outcome** |
|  | keywords: Any cardiovascular event, heart insufficiency, myocardial infarction, stroke (ischemic and small vessel disease), other cerebral diseases, hypertension, stroke, stroke or CVD-specific death, and all-cause mortality. |
| Search #3 | risk[tiab] OR atherosclerotic[tiab] OR “heart disease”[tiab] OR “CVD”[tiab] OR incident[tiab] OR recurrent[tiab] OR “cardio*”[tiab] OR “cardiac*”[tiab] OR “heart*”[tiab] OR “stroke*”[tiab] OR “stroke risk”[tiab] OR “cereborovascular*”[tiab] OR “cerebral”[tiab] OR “coronary heart disease”[tiab] OR “sudden cardiac death”[tiab] OR “coronary*”[tiab] OR “vascular*”[tiab] OR “atrial fibrillation”[tiab] OR “death*”[tiab] OR “mortality”[tiab] OR “all-cause mortality”[tiab] OR “died”[tiab] OR “major adverse cardiac events”[tiab] OR “adverse event*”[tiab] OR “all-cause mortality*”[tiab] OR “small vessel disease”[tiab] OR “lethal*”[tiab] OR “thrombo*”[tiab] OR “arteri*”[tiab] OR "stent"[tiab] OR “myocardial infarction*”[tiab] OR “cardiac infarction”[tiab] OR “cardiac arrest”[tiab] OR stroke[tiab] OR “atherogenic”[tiab] OR “insufficiency*”[tiab] OR ”occlusion”[tiab] OR “ischemi*”[tiab] OR “cardiac event*”[tiab] “kidney function”[tiab] OR “renal function*”[tiab] OR “filtration rate*”[tiab] OR “kidney dysfunction”[tiab] OR “kidney disease”[tiab] OR "hypertension"[Mesh] OR "Cardiovascular Diseases"[Mesh] OR "Vascular Diseases"[Mesh] OR "Stroke"[Mesh] OR "Cerebrovascular Disorders"[Mesh] OR "Coronary Artery Disease"[Mesh] OR "Heart Diseases"[Mesh] OR “Risk Factors” [Mesh] |
| Exclude Search #4 | keywords: Animal studies, cell cultures, inherited disorders causing elevated TMA or TMAO |
|  | Mouse[tiab] OR mice[tiab] OR rat[tiab] OR rats [tiab] OR murine[tiab] OR infants[tiab] OR pregnant[tiab] OR “cell culture*”[tiab] OR animals[tiab] OR cancer[tiab] OR dementia[tiab] OR “lactat*”[tiab] OR “child*”[tiab] |
| Combined search | #1 AND #2 AND #3 NOT #4 |
| Filter(s) | Include only English articles. Time limit since 01.01.2016. |

**Supplemental Table 3.** The OHAT Risk of Bias Rating (internal validity of the study)-TMAO-umbrella systematic review and meta-analyses for Co, CaCo, CrSe (cohort, case-control, and cross sectional) study designs.

| **ROB Domain** | **Promoting question** | **Example** | **ROB** | |  | **Explanation** |
| --- | --- | --- | --- | --- | --- | --- |
| **Selection bias^1^** | 1-Did selection of study participants result in appropriate comparison groups? | Evaluate if exposed and non-exposed were likely to be equally selected and remain in the study |  | |  | **Definitely low** risk of bias:  There is direct evidence of low risk of bias practices. |
|  |  |  |  | |  | **Probably low** risk of bias:  There is indirect evidence of low risk of bias practices OR it is deemed that deviations from low risk of bias practices for these criteria during the study would not appreciably bias results, including consideration of direction and magnitude of bias. |
|  |  |  |  | |  | **Probably High** risk of bias:  There is indirect evidence of high risk of bias practices OR there is insufficient information (e.g., NR or “NR”) provided about relevant risk of bias practices. |
|  |  |  |  | |  | **Definitely High** risk of bias:  There is direct evidence of high risk of bias. |
| Short explanation**:** | | | | | | |
| **Confounding Bias^2^** | 2- Did the study design and analysis account for important confounding and modifying variables? | Confounding variables or confounders include any factor that is:  1) associated with the exposure,  2) an independent risk factor for a given outcome, and  3) unequally distributed between study groups |  | |  | **Definitely low** risk of bias:  There is direct evidence of low risk of bias practices. |
|  |  |  |  | |  | **Probably low** risk of bias:  There is indirect evidence of low risk of bias practices OR it is deemed that deviations from low risk of bias practices for these criteria during the study would not appreciably bias results, including consideration of direction and magnitude of bias. |
|  |  |  |  | |  | **Probably High** risk of bias:  There is indirect evidence of high risk of bias practices OR there is insufficient information (e.g., NR or “NR”) provided about relevant risk of bias practices. |
|  |  |  |  | |  | **Definitely High** risk of bias:  There is direct evidence of high risk of bias. |
| Short explanation**:** | | | | | | |
| **Attrition/Exclusion Bias^3^** | 3- Were outcome data complete without attrition or exclusion from analysis? | Are there nonresponders, dropout, loss to follow up, or exclusion from the analysis? Were exclusions addressed using sufficient statistical methods? Was attrition potentially related to the exposure and outcome? |  | |  | **Definitely low** risk of bias:  There is direct evidence of low risk of bias practices. |
|  |  |  |  | |  | **Probably low** risk of bias:  There is indirect evidence of low risk of bias practices OR it is deemed that deviations from low risk of bias practices for these criteria during the study would not appreciably bias results, including consideration of direction and magnitude of bias. |
|  |  |  |  | |  | **Probably High** risk of bias:  There is indirect evidence of high risk of bias practices OR there is insufficient information (e.g., NR or “NR”) provided about relevant risk of bias practices. |
|  |  |  |  | |  | **Definitely High** risk of bias:  There is direct evidence of high risk of bias. |
| Short explanation**:** | | | | | | |
| **Detection Bias^4^** | 4- Can we be confident in the exposure characterization? | Three important factors for assessing bias in the outcome assessment:  1) the objectivity of the outcome assessment,  2) consistency in measurement of outcomes, and  3)- Blinding of subjects for their own exposure, |  |  | | **Definitely low** risk of bias:  There is direct evidence of low risk of bias practices. |
|  |  |  |  |  | | **Probably low** risk of bias:  There is indirect evidence of low risk of bias practices OR it is deemed that deviations from low risk of bias practices for these criteria during the study would not appreciably bias results, including consideration of direction and magnitude of bias. |
|  |  |  |  |  | | **Probably High** risk of bias:  There is indirect evidence of high risk of bias practices OR there is insufficient information (e.g., NR or “NR”) provided about relevant risk of bias practices. |
|  |  |  |  |  | | **Definitely High** risk of bias:  There is direct evidence of high risk of bias. |
| Short explanation**:** |  |  |  |  | |  |
| **Detection Bias^4^** | 5- Can we be confident in the outcome assessment? | 1) blinding of the outcome assessors (for knowledge of the exposure)  2) the objectivity of the outcome assessment |  |  | | **Definitely low** risk of bias:  There is direct evidence of low risk of bias practices. |
|  |  |  |  |  | | **Probably low** risk of bias:  There is indirect evidence of low risk of bias practices OR it is deemed that deviations from low risk of bias practices for these criteria during the study would not appreciably bias results, including consideration of direction and magnitude of bias. |
|  |  |  |  |  | | **Probably High** risk of bias:  There is indirect evidence of high risk of bias practices OR there is insufficient information (e.g., NR or “NR”) provided about relevant risk of bias practices. |
|  |  |  |  |  | | **Definitely High** risk of bias:  There is direct evidence of high risk of bias. |
| Short explanation**:** | | | | | | |
| **Selective reporting bias^5^** | 6- Were all measured outcomes reported? |  |  | |  | **Definitely low** risk of bias:  There is direct evidence of low risk of bias practices. |
|  |  |  |  | |  | **Probably low** risk of bias:  There is indirect evidence of low risk of bias practices OR it is deemed that deviations from low risk of bias practices for these criteria during the study would not appreciably bias results, including consideration of direction and magnitude of bias. |
|  |  |  |  | |  | **Probably High** risk of bias:  There is indirect evidence of high risk of bias practices OR there is insufficient information (e.g., NR or “NR”) provided about relevant risk of bias practices. |
|  |  |  |  | |  | **Definitely High** risk of bias:  There is direct evidence of high risk of bias. |
| Short explanation**:** |  |  |  | |  |  |
| **Other bias^6^** | 7- Were there no other potential threats to internal validity (e.g., statistical methods were appropriate and researchers adhered to the study protocol)? | - Were statistical methods appropriate? - Did researchers adhere to the study protocol? - Did the study design or analysis account for important confounding and modifying variables (including unintended co-exposures) in experimental studies? |  | |  | **Definitely low** risk of bias:  There is direct evidence of low risk of bias practices. |
|  |  |  |  | |  | **Probably low** risk of bias:  There is indirect evidence of low risk of bias practices OR it is deemed that deviations from low risk of bias practices for these criteria during the study would not appreciably bias results, including consideration of direction and magnitude of bias. |
|  |  |  |  | |  | **Probably High** risk of bias:  There is indirect evidence of high risk of bias practices OR there is insufficient information (e.g., NR or “NR”) provided about relevant risk of bias practices. |
|  |  |  |  | |  | **Definitely High** risk of bias:  There is direct evidence of high risk of bias. |
| Short explanation**:** | | | | | | |

**Supplemental Table 4**. List of excluded articles (n = 119) in the full text stage and reasons for exclusion.

| **PMIDs** | **#** | **Reasons for exclusion** |
| --- | --- | --- |
| 31894847 | 1 | TMAO association with lipids |
| 31373635 | 2 | TMAO and risk of gestational diabetes. |
| 31002427 | 3 | studied association between TMAO and CKD stage (as mean, SD), but no CVD or renal outcome |
| 29607983 | 4 | did not investigate TMAO |
| 29982310 | 5 | TMAO and risk of T2DM |
| 30535087 | 6 | TMAO and risk of gestational diabetes |
| 30606084 | 7 | investigated urinary TMAO as an exposure, but not plasma TMAO. |
| 30846829 | 8 | TMAO was not measured. |
| 30928187 | 9 | study on indoxylsulfate. |
| 28724646 | 10 | association of TMAO with T2DM is not one of the outcomes in our study |
| 28925931 | 11 | association of TMAO with T2DM is not one of the outcomes in our study |
| 29433998 | 12 | TMAO is not studied. |
| 28439531 | 13 | not reporting TMAO in relation to risk of vascular disease |
| 28328981 | 14 | Study not measuring or reporting TMAO |
| 28077427 | 15 | Study on TMAO and colorectal cancer, not on CVD. |
| 27228955 | 16 | not relevant in relation to the planned outcomes (cIMT and TMAO and cIMT and lifestyle intervention). |
| 26650937 | 17 | only 13 people included |
| 26674761 | 18 | Studied effect of diet on plasma concentrations of TMAO/did not investigate any of the outcomes in our study |
| 26956554 | 19 | not about TMAO |
| 26972052 | 20 | Overlap with 23614584. Data in Figure 1 was extracted (same cohort/slightly different outcome) |
| 26864355 | 21 | only metabolites not linked to any of the planned outcomes of the present study |
| 16401621 | 22 | Outcome is accumulation of TMAO in 10 patients undergoing hemodialysis vs. 10 controls |
| 36237188 | 23 | metabolomics not showing quantitative concentrations of TMAO. |
| 25332177 | 24 | content irrelevant, data on TMAO not shown |
| 26413854 | 25 | not possible to compute the effect size from the data |
| 34212778 | 26 | TMAO was not measured |
| 34448864 | 27 | diabetes is not planed as one outcome in the present study. |
| 35506046 | 28 | showing mean (SD) from 19 pts and 19 controls on the difference in TMAO means, but not on OR, RR or HR. |
| 34673277 | 29 | outcome peritonitis is not one of the outcomes in the present study |
| 33765061 | 30 | Study aims and outcomes were unrelated to the present study. Fig. 2 shows prevalence of CAD in patients with TMAO above and below the median. Otherwise no data on adjusted RR in this cross sectional study. |
| 33815248 | 31 | major overlap (the population in this study is a subpopulation of the PMID 32282978). |
| 33709132 | 32 | TMAO measured with untargeted metabolomics, no concentrations are presented. |
| 33122777 | 33 | only show median TMAO in CAD patients and controls, but no risk estimation |
| 31940332 | 34 | outcome is diabetes, insulin resistance and HbA1C, but not cardiovascular disease. |
| 31978943 | 35 | very general about gut bacteria and association with foods. Not showing TMAO concentrations. |
| 32139154 | 36 | TMAO is not measured |
| 31353762 | 37 | This PMID 31353762 is the same study cohort as in PMID: 30370976 |
| 33477634 | 38 | Risk of kidney graft failure is not one of the planned outcomes |
| 32598563 | 39 | 1- Major overlap in the study population suspected with (PMIDs: 32598563, 33454370, 31412713 and 26869641) (authors contacted - no reply); 2- Caucasians were considered the reference group and the HR in South Asians and Japanese were calculated; 3- for the whole population of the study (3 cohorts) there is no adjusted HR shown |
| 32576072 | 40 | Study on n = 69 pts on maintenance hemodialysis, the outcome was hospitalization for any reason; only 9 people were hospitalized due to CVD. The results were shown for 20 hospitalization events combined (due to other multiple non-CVD related conditions). |
| 33052545 | 41 | Not showing risk of stroke in relation to TMAO, TMAO concentrations are shown in a Figure, but there is not quantitative data on OR, RR, or HR. |
| 31683880 | 42 | showed TMAO by CKD stages and measured GFR. There is no measure of effect size that can be combined with other studies among renal patients. |
| 31412713 | 43 | Research letter. Insufficient information on statistical analyses, adjustments or outcome measured. Possible overlap in the population with PMIDs: 33454370, 26869641, and 32598563. Corresponding author contacted several time without response. |
| 28469156 | 44 | did not report TMAO or risk associated with TMAO concentrations |
| 28772054 | 45 | did not measure TMAO |
| 29330424 | 46 | no quantitative investigation of TMAO in plasma/serum |
| 28621609 | 47 | TMAO and cancer |
| 29519360 | 48 | TMAO was not measured or reported |
| 28755411 | 49 | measured TMAO in a group of healthy people in a cross sectional study design, but did not link it to a health outcome (only correlations with eGFR are shown) |
| 30551613 | 50 | Outcome BMI and metabolic syndrome/not within the scope of the present review |
| 31420758 | 51 | outcome is cognitive impairment post stroke. Outcome is not within the scope of the present review. |
| 30762730 | 52 | did not report numerical values for TMAO, no clear associations were shown between TMAO and CHD (only AUC, but cut-off value is unclear). |
| 30487369 | 53 | Reported only mean (SD) of TMAO in 22 pts with HF vs. 10 hospital based controls. |
| 24497336 | 54 | Multiple publications/data not extractable. n = 3903 people were recruited at the Cleveland Clinic; show mostly interaction between TMAO and choline or betaine on the HR of MACE. |
| Not PubMed listed | 55 | Chinese language; Liu ZD, Zheng N. Correlation of TMAO and Lp-PLA2 levels in patients with atherosclerotic ischemic stroke. Chinese Journal of Clinical Pharmacology and Therapeutics 2017; 22(06):684–8 |
| Not PubMed listed | 56 | PhD thesis Chinese language: Xu YF. A preliminary study on the relation between stroke and TMAO and the effect of Tan-Huo-Fang on Trimethylamine N-oxide. M.Sc. Thesis, Capital Medical University. 2017 |
| Not PubMed listed | 57 | PhD thesis Chinese language: Wang KC. Correlation between Gut Microbiota Dysbiosis and Elevate Blood TMAO Levels in Patients with Acute Ischemic Stroke. M.Sc. Thesis, Whenzhou Medical university.2020. |
| Not PubMed listed | 58 | PhD thesis Chinese language: Liao SX. A study about the relation between the stroke with gut microbiota and its metabolite-trimethylaming N-oxide. M.Sc. Thesis, Southern Medical University. 2016 |
| Not PubMed listed | 59 | PhD thesis Chinese language: Zhai QJ. The Prognostic Value of Plasma Thrimethylamine N-oxide Levels in Cerebrovascular Diseases. doctoral dissertations, Southern Medical University. 2020. |
| Not PubMed listed | 60 | Poster: HIGHER PLASMA TRIMETHYLAMINE-N-OXIDE IS ASSOCIATED WITH GREATER ATHEROSCLEROTIC BURDEN QUANTIFIED BY THE SYNTAX SCORE. Vichai Senthong, Xinmin Li, et al. JACC. 2015 Mar, 65 (10_Supplement) A1676 |
| Not PubMed listed | 61 | Chinese journal: Xu YY, Xu DJ, Jin KK, Wang KC. Relationship between trimethylamine N-oxide inperipheral blood of acute ICS patients. Chin J Geriatr Heart Brain Vessel Di. 2022; 24(01):63–6 |
| Not PubMed listed | 62 | Chinese journal: JX, Zhang AQ, Gao RJ, Li ZR, Zhu RX, Yao Y, et al. Correlation Study between Serum Trimethylamine-N-oxide and the Risk of Acute Ischemic Stroke. Chinese General Practice. 2022; 25(09):1130–5. |
| Not PubMed listed | 63 | Poster: Senthong V, Wang Z, Wu Y, Tang WH, Hazen S. Elevated plasma trimethylamine N oxide is associated with poor prognosis in patients with peripheral artery disease. J Am Coll Cardiol. 2015; 65: 2076–9. |
| Not PubMed listed | 64 | Unable to identify this article on public domains: Liu YQH. The Relationship Between Plasma Trimethylamine Oxide Level and Poor Prognosis in Patients With Acute Heart Failure. Hunan: Nanhua University (2019). |
| 23563705 | 65 | Mainly on L-Carnitineand association of carnitine/TMAO with CVD. Data on TMAO alone as exposure can not be extracted from the paper (Fig 4, f); clinical characteristics not well described. |
| 25336191 | 66 | The outcome is not appropriate - Colorectal Cancer |
| 35856225 | 67 | Chinese Language |
| Not PubMed listed | 68 | Chinese Language: Xu K-Z, Lin L-M, Wu Y, Xu J-H, Wu M-F. Relationship between the plasma level of trimethylamine N-oxide and complication risk in patients with acute myocardial infarction. Chin J Arterioscler. 2018;26:497–502 |
| Not PubMed listed | 69 | Chinese Language: Zhao XH, Song ZJ, Yang J, Liu P, Gen B, Wang HZ. The Predictive Value of Periphera Blood TMAO and CD26P on the Prognosis of Patients with Acute Ischemic Stroke. Labeled Immunoassays and Clinical Medicine. 2021; 28(07):1217–21 |
| Not PubMed listed | 70 | Chinese Language: Kai-Zu XU, Lin LM, Ying WU, Jin-Hong XU, Mei-Fang WU, Cardiology DO. Relationship between plasma trimethylamine-N-oxide levels and complication risk in patients with acute myocardial infarction. Chinese Journal of Arteriosclerosis. 2017. |
| Not PubMed listed | 71 | Chinese Language: Yu-Lan Z, Pei-Sheng Z. The connection between trimethylamine oxide, an intestinal flora metabolite, and atrial fibrillation. World J Complex Med. 2018;4:30–3. |
| 38892223 | 72 | Quantitative data not extractable, very bad study quality |
| 38110027 | 73 | acute Kidney Injury not foreseen as one outcome of the present study |
| 37944962 | 74 | not meeting minimal scientific quality |
| 37737730 | 75 | No association measures are shown between TMAO and HF |
| 37730687 | 76 | Outcome is TMAO, results cannot be combined with other studies |
| 37673746 | 77 | Outcome is TMAO and Framingham score |
| 37445971 | 78 | TMAO and the outcome of acute ischemic stroke after Recanalization therapy measures |
| 35831155 | 79 | Only TMAO concentrations are reported among the groups, no statistical measures as shown |
| 34365799 | 80 | Unplanned outcomes: echocardiographic parameters and cardiac magnetic resonance imaging |
| 33334254 | 81 | Only TMAO concentrations are reported, but no association measures. |
| 32870170 | 82 | TMAO is an outcome not an exposure |
| 32629663 | 83 | a study protocol |
| 32599111 | 84 | Only TMAO levels ae reported, no measure of association |
| 27600460 | 85 | Low sample size, investigated optical coherence tomography (not planned as outcome) and showed only correlation coefficient |
| 37553923 | 86 | NIHSS scores to predict neurological deterioration at 7d; unclear what happened with patients who did not stay 7 days in the hospital; the study did not adjust for baseline NIHSS and analyzed the data as a case control study. |
| 36695932 | 87 | is not showing any measure of associations with spontaneous subarachonoid hemorrhage, except simple correlation coefficient. |
| 35787338 | 88 | exposure is statin, outcome is TMAO and subsequent outcome |
| 35278373 | 89 | major overlap in the populations and outcomes with #62 PMID 30370976. The focus of this paper is rather the combination of several markers, instead of TMAO alone. |
| 34233325 | 90 | Flaw in methods and data analyses |
| 32270523 | 91 | the study focus is trimethyllysine; little or no focus in the data analyses on TMAO (e.g., supplemental data on subgroups by riboflavin); The study cohort The Western Norway Coronary Angiography Cohort (WECAC) has been published before in relation to the topic of TMAO and CVD (# PMID 35916742 and 29957250) |
| 32060329 | 92 | the study focus is on gut bacteria, no relevant data about the association between TMAO and CVD is presented in the paper |
| 31894847 | 93 | the outcome lipoproteins is not one of the outcomes we planned to look for in this study |
| 31666184 | 94 | outcome is thrombocytes functional tests is not any of the planned outcomes in this study. |
| 29702430 | 95 | introduced new definition for patients [with a high plaque burden not explained by traditional risk factors] not compatible with other studies, presented only linear regression analyses. The outcome in the study is questionable. |
| 32595007 | 96 | Post-hoc analyses of the OCTAMI register (PMID: 33792267 and 38567028); outcome not relevant for our study |
| 33792267 | 97 | Questionable design and TMAO values |
| 33500103 | 98 | Outcome Healed Plaque; unreliable results of logistic regression analyses |
| 26972052 | 99 | major overlap- with PMID:23614584 (outcome here is Major adverse cardiovascular events defined as 1- mortality from any cause and 2- nonfatal MI or stroke) |
| 27567042 | 100 | [major concerns in study design] participants were using antibiotics at admission |
| 28870405 | 101 | [major concerns in study design]; > 50 % of the participants were using antibiotics at admission |
| 29061990 | 102 | (Exclude; Authors confirmed overlap with PMID: 33993608- that is already included) |
| 29729869 | 103 | (exclude outcome not relevant and unclear data analyses) |
| 33477634 | 104 | (exclude; outcome was unplanned) |
| 38892223 | 105 | [insufficient description of results] |
| 37944962 | 106 | insufficient scientific quality |
| 36420057 | 107 | low quality design and conduct, TMAO concentrations measured with ELISA Kit, unit of TMAO not reported. |
| 33500103 | 108 | major overlap with (PMID: 38567028 where the outcomes are likely more reliable than in the present study. |
| 31958034 | 109 | Same study as PMID: 29268932 |
| 26597155 | 110 | effect size median (IQR) |
| 26825179 | 111 | effect size not plausible |
| 26554714 | 112 | effect size |
| 30862856 | 113 | effect size, outcome not relevant |
| 38773380 | 114 | effect size |
| 37610752 | 115 | effect size |
| 36839170 | 116 | effect size |
| 36771464 | 117 | effect size |
| 27152465 | 118 | outcome not relevant |
| 36593094 | 119 | outcome not compatible with other studies |

| **Supplemental Table 5.** Subgroup analyses of cohort studies by risk of bias (ROB) in individual studies on the association between TMAO concentrations and MACE. | | | | | | | |
| --- | --- | --- | --- | --- | --- | --- | --- |
| **Subgroups** | **Pooled HR (95%CIs)^3^** | | **N Publications^4^** | | | **I^2^ (heterogeneity)** | |
| **Overall risk of bias^1^** |  | |  | | |  | |
| Low | 1.15 (1.08-1.23) | | 17 | | | 40.5% | |
| High | 1.25 (1.13-1.38) | | 19 | | | 80.5% | |
| **Selection bias^2^** |  | |  | | |  | |
| Low | 1.15 (1.07-1.24) | | 13 | | | 41.8% | |
| High | 1.23 (1.13-1.34) | | 22 | | | 78.4% | |
| **Confounding^2^** |  | |  | | |  | |
| Low | 1.16 (1.08-1.24) | | 20 | | | 69.5% | |
| High | 1.30 (1.13-1.50) | | 16 | | | 74.1% | |
| **Attrition^2^** |  | |  | | |  | |
| Low | 1.16 (1.08-1.25) | | 15 | | | 32.6% | |
| High | 1.22 (1.12-1.33) | | 20 | | | 79.4% | |
| **Detection bias exposure^2^ (TMAO)** | | |  | | |  | |
| Low | 1.14 (1.08-1.21) | | 25 | | | 45.4% | |
| High | 1.47 (1.24-1.75) | | 10 | | | 88.0% | |
| **Detection bias outcome^2^** |  | |  | | |  | |
| Low | 1.13 (1.07-1.20) | | 20 | | | 50.9% | |
| High | 1.38 (1.17-1.63) | | 15 | | | 81.6% | |
| **Selective reporting^2^** |  | |  | | |  | |
| Low | 1.13 (1.07-1.20) | | 27 | | | 63.6% | |
| High | 1.69 (1.30-2.19) | | 8 | | | 69.7% | |
| **Other sources of bias^2^** |  | |  | | |  | |
| Low | 1.12 (1.06-1.18) | | 13 | | | 0.4% | |
| High | 1.25 (1.15-1.36) | | 23 | | | 79.9% | |
| ^1^ Low overall ROB (up to three out of 7 domains of the OHAT tool have high ROB); High overall ROB (at least 4 domains have high ROB).  ^2^ low and probably low ROB were considered as low ROB. High ROB and probably high ROB were considered as high ROB.  ^3^ Meta-analysis of confounder-adjusted HR (95%CIs) was conducted using the random-effects model.  ^4^ Number of studies may not sum up to 34 because of overlapping publications of different quality score. | | | | | | | |
| **Supplemental Table 6.** Additional subgroup analyses according to factors related to design or conduct of individual cohort studies on the association between TMAO concentrations and MACE. | | | | | | |  |
| **Subgroups** | | **Pooled HR (95%CIs)^1^** | | **N** | **I^2^ (heterogeneity)** | |  |
| **Publication year** | |  | |  |  | |  |
| 2016- 2020 | | 1.28 (1.12-1.47) | | 13 | 81.7% | |  |
| 2021-2024 | | 1.16 (1.09-1.24) | | 21 | 54.8% | |  |
| **Follow up duration** | |  | |  |  | |  |
| Short (mean follow up time < 5 years) | | 1.29 (1.12-1.48) | | 12 | 62.6% | |  |
| Long (mean follow up ≥ 5 years) | | 1.16 (1.09-1.24) | | 22 | 71.1% | |  |
| **Adjustment for hypertension** | |  | |  |  | |  |
| Yes | | 1.20 (1.12-1.29) | | 26 | 65.4% | |  |
| No | | 1.21 (1.06-1.39) | | 10 | 75.7% | |  |
| **Adjustment for renal function** | |  | |  |  | |  |
| Yes | | 1.20 (1.13-1.28) | | 33 | 72.7% | |  |
| No | | 1.19 (1.002-1.41) | | 3 | 52.0% | |  |
| **Renal disease at baseline visit** | |  | |  |  | |  |
| Yes | | 1.26 (1.11-1.43) | | 11 | 55.0% | |  |
| No | | 1.18 (1.10-1.26) | | 23 | 76.6% | |  |
| **Country** | |  | |  |  | |  |
| USA + Canada | | 1.10 (1.02-1.19) | | 9 | 52.4% | |  |
| China | | 1.32 (1.11-1.57) | | 6 | 58.8% | |  |
| Central Europe and Nordic countries | | 1.27 (1.14-1.42) | | 18 | 76.8% | |  |
| New-Zealand | | 0.60 (0.33-1.10) | | 1 | - | |  |
| Nordic countries alone | | 1.09 (1.00-1.18) | | 5 | 9.2% | |  |
| ^1^ Meta-analysis of confounder-adjusted HR (95%CIs) was conducted using the random-effects model. | | | | | | |  |

| **Supplemental Table 7.** Meta-analyses and subgroup analyses of the associations between TMAO and MACE from case-control studies reporting adjusted OR (95%CIs). | | | |
| --- | --- | --- | --- |
| **Meta-analyses** | **Pooled OR (95%CI)^4^** | **Predictive interval** | **I^2^ heterogeneity** |
| Overall, n = 18 case-control studies^1^ [15;26;27;42;44;48;64;66;71-80] | 1.38 (1.24-1.54) | 0.95-2.05 | 89.1% |
| Overall estimate corrected for publication bias (9 studies were missing) | 1.05 (0.94-1.18) | - | - |
| ***Subgroup analyses on TMAO and MACE from case-control studies*** | | | |
| Case control studies with a low overall risk of bias^2^, n = 7 | 1.08 (1.00-1.17) | 0.89-1.33 | 77.7% |
| Case control studies with an high overall risk of bias^2^, n = 11 | 1.93 (1.40-2.66) | 0.63-5.97 | 87.0% |
| Case control studies with no adjustment for hypertension, n = 6 | 2.32 (1.21-4.43) | 0.23-23.6 | 95.1% |
| Case control studies adjusted for hypertension, n = 12 | 1.24 (1.09-1.41) | 0.85-1.82 | 72.3% |
| No adjustment for renal function marker, n = 7 | 1.55 (1.29-1.86) | 0.95-2.54 | 94.7% |
| Adjusted for renal function marker, n = 11 | 1.29 (1.13-1.49) | 0.87-1.92 | 59.4% |
| Simultaneously adjusted for hypertension and renal function, n = 9 | 1.23 (1.08-1.42) | 0.84-1.84 | 59.7% |
| Subgroup of studies conducted in Asia/China, n = 10 | 2.02 (1.41-2.89) | 0.56-7.18 | 92.0% |
| Subgroup of studies conducted in all other countries except Asia/China, n = 8 | 1.14 (1.002-1.29) | 0.79-1.63 | 71.7% |
| ^1^ Publication bias: the Egger’s test Intercept = 2.63 (1.40-3.85); t-value 4.55; p = 0.0003.  ^2^ High overall risk of bias (ROB) was defined based on the number of domains with high ROB in OHAT tool.  ^3^ A single study from Singapore was not included in subgroup analyses by country.  ^4^ Adjusted OR (95%CIs) were meta-analyzed using the random effects model. | | | |

**Supplemental Table 8.** List of established cohort and case-control studies that contributed to the evidence on TMAO.

**Cohort studies**

| **First Author** | **PMID** | **Country of participants** | **All reported outcomes as defined in the primary publications** |
| --- | --- | --- | --- |
| M. Trøseid [81] | 25382824 | Norway, Oslo | Composite end–point defined as all–cause and anticipated–mortality (i.e. heart transplantation) |
| Tang WH [2] | 23614584 | USA, Cleveland | Major adverse cardio-vascular events defined as 1– mortality from any cause and 2– nonfatal MI or stroke |
| Tang WH [4] | 25444145 | USA, Cleveland | All–cause mortality |
| Tang WH [5] | 25459686 | USA, Cleveland | Composite endpoint of adverse clinical events (all–cause mortality and cardiac transplantation) |
| Lever M [70] | 25493436 | Christchurch, New Zealand | All–cause mortality |
| Tang WH [6] | 25599331 | USA, Cleveland | All–cause mortality at 5 yrs in CKD patients |
| Kaysen GA [11] | 25802017 | USA 56 dialysis centers across the US | mortality and cause specific hospitalization (in a subgroup with Medicare beneficiaries) |
| Müller DM [54] | 26554714 | Austria, Feldkirch | Major adverse cardio-vascular events (defined as cardiac mortality, MI, or stroke) |
| Missailidis C [82] | 26751065 | Sweden, Stockholm | All-cause mortality |
| Skagen K [83] | 26868510 | Norway, Oslo | Mortality from vascular event (stroke and MI) |
| Suzuki T [84] | 26869641 | UK, Leicester | Composite of mortality or rehospitalisation due to HF |
| Kim RB [28] | 27083288 | Multicenter study across Canada | Occurrence of ischemic event (myocardial infarction, unstable angina, ischemic stroke, coronary revascularization, new onset of CHD (proven by cardiac catheterization), amputation due to peripheral vascular disease, peripheral artery bypass, and gangrene) and recurrent ischemic event |
| Shafi T [12] | 27436853 | USA, multicenter study | - Cardiac mortality: defined as defined as mortality due to coronary events, arrhythmias, sudden cardiac mortality, congestive heart failure, or cerebrovascular events. - Sudden cardiac mortality was defined as witnessed mortality with preceding duration of symptoms less than 24 hours or unwitnessed mortality, or unwitnessed unexpected mortality with symptom duration less than the interval since the last dialysis session - First cardio-vascular event or any cause mortality - All–cause mortality |
| Robinson–Cohen C [85] | 27513517 | USA, Seattle | - The primary renal outcome was the relative annualized change in kidney function, defined by the exponentiated slope of log–transformed eGFR values. - All–cause mortality |
| Senthong V [7] | 27792653 | USA, Cleveland | All–cause mortality |
| Meyer KA [17] | 27792658 | USA | - Incident CAC (Coronary artery calcium) - 10 yrs progression of CAC - 10 yrs change in eGFR (progression of renal dysfunction) - 10 yrs change in Urine albumin/creatinine ratio (progression of renal function) |
| Tang WH [8] | 27864387 | USA, Cleveland | - All–cause mortality at 5 yrs - Major adverse events (defined as mortality, non–fatal MI, and non–fatal stroke) |
| Suzuki T [86] | 28062632 | Leicester, UK | - Composite outcome of all–cause mortality and reinfarction (mortality/MI) at 6 mo. and 2 yrs - All–cause mortality at 6mo and 2 yrs |
| Li XS [87] | 28077467 | USA, Cleveland | - All–cause mortality at 7 yrs - Major adverse cardio-vascular events (MACE): defined as a composite of MI, stroke, revascularization, or all–cause mortality at 30 days - Major adverse cardio-vascular events (MACE): defined as a composite of MI, stroke, revascularization, or all–cause mortality at 6 mo |
|  |  | Switzerland (multi–center) | Major adverse cardio-vascular events (MACE): defined as a composite of MI, stroke, revascularization, or all–cause mortality at 1 year |
| Wu C [88] | 29540587 | China (multi–center) | New lesion detected at MRI (1–3 days post carotid artery stenting) |
| Svingen GFT [49] | 29957250 | Norway (multi–center) | - Arterial fibrillation – WECAC cohort - Arterial fibrillation – HUSK cohort |
| Haghikia A [58] | 29976769 | Germany (multi–center) | Combined vascular end–point composed of MI, recurrent stroke and cardio-vascular mortality during 1–year follow–up |
| Suzuki T [65] | 30370976 | Multicenter across Europe | - All–cause mortality (at 1, 2 and 3 yrs) - Composite event of mortality combined with rehospitalisation due to HF (mortality/HF) at 1, 2 and 3 yrs |
| Reiner MF [29] | 30553164 | Switzerland, multicenter | All–cause mortality |
| Stubbs JR [13] | 30665924 | USA | - Vascular composite (CV mortality, MI, hospitalization for unstable angina, PVE, stroke) - EVOLVE primary composite (all–cause mortality, MI, hospitalization for unstable angina, HF, PVE) - All–cause mortality - CVD mortality - MI - Peripheral vascular event - Stroke |
| Winther SA [50] | 31123156 | Denmark, Copenhagen | - Incidence of combined CVD (defined as: comprising CVD–related mortality, ischemic heart disease (including nonfatal myocardial infarction [ICD–0codesI20–I25]), non-fatal stroke, coronary interventions, or peripheral arterial interventions (including amputations) - Coronary events defined as (nonfatal and fatal myocardial infarction [ICD–10 codes I21–I24]) or coronary intervention (percutaneous arterial intervention or coronary bypass grafting [procedural codes KFNA–KFNG]) - Stroke (fetal or non–fatal: ICD–10 codes I61–I66) - Heart failure (hospitalization as a result of heart failure (ICD–10 code I50)) - ESRD (CKD stage 5 (ICD–10 code N18.5), initiation of permanent dialysis, renal transplantation, eGFR,15mL/min/1.73m2, or mortality as a result of chronic renal failure) - All–cause mortality - Cardio-vascular mortality |
| Rexidamu M [89] | 31142624 | China, Shanghai | Stroke functional scores at hospital discharge (favorable mRS score = 0–2 points, while unfavorable outcome when mRS score = 3–6 points) |
| Zhai Q [90] | 31332666 | China, Jiangsu | - Functional outcome at 3 mo. after stroke, evaluated by the modified Rankin scale mRS score 3–6 (bad outcome) - All–cause mortality at 3 mo. |
| Nam HS [91] | 31590480 | Korea, Seoul | MACE defined as recurrent stroke, MI, or mortality |
| Roncal C [92] | 31666590 | Spain, Pamplona | - CV mortality - All–cause mortality - MACE defined as; amputation, stroke, myocardial infarction and all–cause mortality |
| Hou L [93] | 31928326 | China, Mianyang | Early neurological deterioration defined as increase in HIHSS score by 2 or more points within 3 days after admission |
| Zhou X [94] | 31960610 | China multicenter | - Major adverse cardiac events (MACE) [including all–cause mortality, HF rehospitalization, or recurrent MI] - All–cause mortality |
| Tan C [95] | 32082246 | China, Guangzhou | - Major ischemic event within 90 days (and 12 mo.), defined as ischemic stroke, myocardial infarction, or mortality from an ischemic vascular event. - unfavorable functional outcome (mRS score ≥3) within 90 days and 12 mo. |
| Croyal M [63] | 32301490 | France | - MACE (defined as: a composite of CV mortality, nonfatal MI, or nonfatal stroke) - All–cause mortality |
| Hochstrasser SR [31] | 32866111 | Switzerland, Basel | - In hospital mortality - 90–days mortality |
| Zhang P [96] | 32985309 | China, Shanghai | - Cardio-vascular mortality - All–cause mortality |
| Israr MZ [97] | 33454370 | UK, Leicester | - All–cause mortality at 30 days and 1 year - mortality/HF at 30 days and 1 year |
| Fu D [98] | 33596582 | China, Guangzhou | - CV mortality (mortality due to myocardial infarction, cardiac arrhythmia, cardiac arrest, congestive heart failure, cerebrovascular events, and peripheral vascular disease) - All–cause mortality |
| Zhang J [99] | 33647820 | China, Fujian | - Functional impairment was assessed at 3–month using the modified Rankin scale (mRS), and a good functional outcome was defined as mRS of 0–2 points, while a poor outcome was (3–6 points) - All–cause mortality within 3–month. |
| Winther SA [51] | 33657115 | Denmark, Copenhagen | - All–cause mortality - CV–related mortality - CVD events - eGFR decline ≥ 30% |
| Kinugasa Y [100] | 33734604 | Japan, Yanago | - Composite endpoints of: mortality due to cardiac causes and first events of unplanned rehospitalization for HF during the follow–up period. - Mortality from a cardiac cause was defined as Mortality due to HF and sudden mortality. - HF Hospitalization |
| Flores–Guerrero JL [46] | 33993608 | The Netherland, Groningen | - All–cause mortality - Cardio-vascular mortality |
| Flores–Guerrero JL [45] | 34073908 | The Netherland, Zwolle | Cardio-vascular mortality |
| Wei H [101] | 34159537 | China, Wuhan | Primary: the composite outcome of cardio-vascular mortality, which infers a cardiac origin (such as sudden cardiac mortality, HF, acute myocardial infarction, cardio-vascular procedures, or other cardio-vascular causes) unless a noncardiac cause was identified, or heart transplantation [results refer to 7–year cardio-vascular mortality or transplantation]. |
| Lee Y [19] | 34398665 | USA, multicenters across the country | First occurrence and recurrence of atherosclerotic cardio-vascular disease defined as: fatal and nonfatal MI, fatal coronary heart disease, stroke (fatal and nonfatal), sudden cardiac mortality, and other atherosclerotic mortality. |
| Ringel C [64] | 34583200 | Cohorts from Germany and Europe | - All–cause mortality |
| Chang D [102] | 34724845 | China, Peking | - CVD mortality (mortality due to MI, congestive heart failure, cerebral bleeding, cerebral infarction, arrhythmia, peripheral arterial disease and sudden mortality) - All–cause mortality |
| Eyileten C [103] | 34778397 | Austria, Vienna | CV–related mortality (include mortality that result from an acute myocardial infarction, sudden cardiac mortality, mortality due to heart failure, mortality due to stroke, mortality due to CV procedures, mortality due to CV hemorrhage, and mortality due to other CV causes) |
| Xu J [35] | 34794334 | China, 201 study sites from 22 provinces and 4 municipalities. | - Recurrent stroke in people with previous ischemic stroke or TIA = new IS and hemorrhagic stroke) - MACE, defined as cardio-vascular mortality, nonfatal myocardial infarction, and nonfatal stroke |
| Xu J [36] | 34816729 | China, multicenter | - MACE (defined as nonfatal stroke recurrence, nonfatal myocardial infarction, or cardio-vascular mortality) - non–fatal stroke recurrence - non–fatal MI - Cardio-vascular mortality (defined as IS, hemorrhagic stroke, sudden cardiac mortality, acute myocardial infarction, and mortality directly caused by heart failure or other cardio-vascular mortality) - All–cause mortality |
| Xu J [37] | 35390802 | China, multicenter | Recurrent stroke (= new ischemic stroke or hemorrhagic stroke) at 3 mo. and 12 mo. |
| Xue J [38] | 36193936 | China (171 Centers) | Stroke recurrence (new ischemic stroke and hemorrhagic stroke) |
| Hernandez L [53] | 35292710 | Sweden, Stockholm | All–cause mortality |
| Chen YY [73] | 35345908 | China, city unclear | - Neurological functional disability as determined by the modified Rankin Scale (mRS) score three mo. after stroke onset. mRS score 0–2 was defined as good outcome and 3–6 as poor outcome - Major vascular event (TIA, recurrent ischemic stroke, acute myocardial infarction) in the 3 mo. after stroke |
| Sapa H [20] | 35351578 | USA, multicenter | - CV mortality(due to MI, stoke, sudden mortality, heart failure, other cardiac death, with other CVD (not cardiac), pulmonary embolism) - All-cause mortality - KFRT = transfer to dialysis (secondary) |
| Li N [104] | 35686339 | China | - Major adverse cardiac events (MACE) defines as a composite of all–cause mortality, recurrence of MI, and rehospitalization due to HF - All–cause mortality - recurrent MI - recurrent HF |
| Amrein M [32] | 35220448 | Switzerland, Basel | - All–cause mortality - cardio-vascular mortality - MI |
| Fretts AM [21] | 35594043 | USA, Forsyth County, North Carolina; Sacramento County, California; Washington County, Maryland; and Pittsburgh, Pennsylvania | Total mortality |
| Stubbs JR [22] | 26229137 | USA, Kansas City, Missouri | All–cause mortality |
| Senthong V [1] | 27287696 | USA, Cleveland | All–cause mortality |
| Schuett K [55] | 29268932 | Germany, Aachen | - All–cause mortality - Cardio-vascular mortality |
| Matsuzawa Y [105] | 31406181 | Japan, Yokohama city | Composite outcome defined as: the incidence of all–cause mortality, nonfatal myocardial infarction, nonfatal stroke, and unstable angina that required coronary, revascularization. |
| Li N [106] | 36354779 | China, Shenzhen (Fuwai Hospital) | - MACE (including all–cause mortality, recurrence of MI, rehospitalization caused by HF, stroke, and any revascularization) - All–cause mortality - Recurrent MI - Recurrent heart failure - Revascularization - Stroke |
| Sanchez–Gimenez R [107] | 36211587 | Spain, Tarragona | MACE was defined as the composite of myocardial infarction, hospitalization for heart failure, and all–cause mortality. |
| Luciani M [33] | 36593094 | Switzerland, multicenter | - All–cause mortality - Cardio-vascular mortality included cardiac mortality (eg, cardiogenic shock, arrhythmia/sudden mortality, cardiac rupture) and other vascular mortalities (eg, stroke, pulmonary embolism, ruptured aortic aneurysm or dissection) and haemorrhagic mortalities were classified as cardio-vascular mortalities - Stroke - Ischemic stroke |
| Meng S [108] | 39732662 | China, Huaian | Recurrent arterial fibrillation in people underwent catheter ablation due to AF |
| Cheng E [109] | 39709651 | Taiwan, Hualien | - Rapid decline in kidney function, defined as an annualized decline in the eGFR of >3 ml/min/1.73 m2 from baseline to the first year of follow–up - Renal composite outcome: a composite of progression of kidney disease, defined as a sustained 40 % decline in eGFR from baseline, initiation of long–term dialysis therapy, preemptive kidney transplantation, or renal mortality |
| Aldujeli A [110] | 39316928 | Republic of Lithuania, Kaunas | Incidence of coronary microvascular dysfunction (defined as index of microcirculatory resistance ≥25 or a coronary flow reserve >2.0 with fractional flow reserve ≥ 8) in people with STEMI after percutaneous coronary intervention in relation to TMAO at 3 mo. |
| Senthong V [69] | 39261513 | Thailand, multicenter | All–cause mortality |
| Tang WHW [25] | 39119698 | USA, multicenter | Incident heart failure |
| Yu X [43] | 39092284 | China, Beijing | Composite outcome of MACEs, which included nonfatal myocardial infarction, stroke, and all–cause mortality |
| Obeid R [57] | 38621431 | Germany, Homburg | - Cardio-vascular events and all–cause mortality combined - Worsening of renal function (defined as a persistent drop of eGFR >50% or progression to continuous need for renal replacement therapy) and All–cause mortality combined - All–cause mortality - Heart failure combined with all–cause mortality - Cardio-vascular event including CV–mortality (but excluding non–CV mortality) |
| Wang M [23] | 38593157 | USA, multicenter | - Incident CKD defined as both a eGFR decline by ≥ 30% and resulting GFR < 60 ml/min per 1.73 m2 - Development of CKD stage 3 or above defined by an algorithm: based on hospitalization ICD codes |
| Zhao X [41] | 38567028 | China, Beijing | MACE; defined as a composite of all–cause mortality, recurrence MI and ischemic cerebrovascular events and revascularization |
| Yu PS [111] | 38267025 | Taiwan, Koahsiung | - ESKD or doubling of serum creatinine - doubling of serum creatinine - All–cause mortality - 30% reduction of eGFR within 2 yrs |
| Shea JW [18] | 37865185 | USA, multicenter | - CVD - CHD - Stroke - Incident CVD events |
| Lemaitre RN [24] | 37581385 | USA multicenter | incident ischemic stroke |
| Davies A [34] | 37574027 | Switzerland, multicenters | Major adverse cardio-vascular events (MACE) were defined as a composite of myocardial infarction, stroke, clinically indicated revascularization, or all–cause mortality |
| Zhao S [112] | 36811471 | China, Harbin | - MACE - Heart failure |
| Yuan X [113] | 36549408 | China, Changsha | MACE is a composite outcome of myocardial infarction, atrial fibrillation, congestive heart failure, acute coronary syndrome, and stroke |
| Dai L [61] | 36166845 | Europe, multicenters | - All–cause mortality - KRT initiation during 48 mo of follow–up - First MACE incidence defined as a hospitalization or comorbidity due to cerebrovascular disease, myocardial infarction, peripheral vascular disease, angina pectoris, arrhythmias, coronary artery disease, or mortality due to myocardial ischemia and infarction, cardiac arrest, or cerebrovascular accident. |
| Konieczny R [114] | 35970148 | Poland, Wroclaw | - All–cause mortality in people with acute coronary syndrome (ACS) - All–cause mortality in people with stable coronary syndrome (SCS) - All–cause mortality in subgroup with Chronic HF - All–cause mortality in subgroup with AF |
| Bjørnestad EØ [52] | 35916742 | Norway–WECAC | - All–cause mortality - CV mortality - non–CV mortality |
| Bjørnestad EØ [52] | 35916742 | Norway–HUSK | - All–cause mortality - CV mortality - Non–CV mortality |
| Wargny M [62] | 35681209 | France | - HF requiring Hospitalization (HFrH) - HFrH and/or CV mortality - All–cause mortality |
| Zhai Q [115] | 32705490 | China, Jiangsu | - Functional outcome at 3 mo. post intracerebral hemorrhage. Patients were dichotomized as good (modified Rankin scale 0–3) and poor (modified Rankin scale 4–6) - Increased mRS score > 6 at 3 mo. post intracerebral hemorrhage |

**Case-control studies**

| **First Author** | **PMID** | **Country of participants** | **Outcome(s) as reported** |
| --- | --- | --- | --- |
| Mente A [116] | 26239008 | Toronto, Hamilton, Edmonton and other, Canada | Prevalent CVD |
| Suzuki T [84] | 26869641 | UK, Leicester | In hospital mortality |
| Guasch-Ferré M [59] | 29080862 | Spain | Composite cardio-vascular events (myocardial infarction, stroke, or mortality from cardio-vascular causes) in TMAO Quartiles |
| Dong Z [80] | 30159101 | China, Harbin | - Risk of CHD in people with T2DM vs controls without CHD and without diabetes - Risk of CHD in people without CHD and T2DM vs. controls |
| Nie J [44] | 30354996 | China, multi-center study | - First nonfatal or fatal ischemic stroke - MACE defined as composite end point of cardio-vascular mortality, MI, or stroke |
| Liang Z [79] | 30370581 | Harbin, China | Ischemic stroke in patients with arterial fibrillation |
| Zheng L [78] | 30508691 | China, Liaoning province | MACE defined as composite end point of cardio-vascular mortality, MI, or stroke |
| Cardona A [26] | 31798892 | Several centers across the US and Canada | CV causes after non-CV surgery, stroke, unexpected death presumed to be from ischemic CV disease occurring within 24 h after the onset of symptoms, and mortality from other vascular diseases (e.g, pulmonary emboli, abdominal aortic aneurysm rupture) |
| Heianza Y [14] | 32081286 | USA | non-fatal MI or fatal CHD |
| Schneider C [77] | 32282978 | Germany, Heidelberg | Stroke |
| Gencer B [66] | 32366163 | 31 countries | - MACE defined as composite end point of cardio-vascular mortality, MI, or stroke - cardio-vascular mortality - recurrent MI - stroke |
| Sun T [76] | 32641646 | China, Guangdong | Ischemic stroke |
| Guo F [75] | 32855821 | China, Changsha | - Coronary artery disease - Severity of arterial stenosis |
| Guo F [117] | 32859154 | China, Changsha | - Heart failure with preserved ejection fraction (HFpEF) - renal dysfunction |
| Dong Z [118] | 32921087 | China, Harbin | Chronic heart failure |
| Dong Z [119] | 33135738 | China, Harbin | Heart failure with preserved ejection fraction (HFpEF) |
| Papandreou C [60] | 33257943 | Spain | - arterial fibrillation - heart failure |
| Tang WHW [48] | 33626384 | Norfolk, UK | Incident CAD |
| Liu G [15] | 33829245 | USA, multicenter | Incident CAD included nonfatal MI, fatal CAD |
| Xu D [74] | 33977104 | China, Wenzhou and Xuanwu | Ischemic large artery atherosclerotic stroke |
| Ringel C [64] | 34583200 | Cohorts from Germany and Europe | - All outcomes in LIFE-CAD cohort: CAD, coronary artery disease - CAP, carotid artery plaque - PAD, peripheral artery disease - Number of affected main coronary arteries |
| Chen YY [73] | 35345908 | China, city unclear | Stroke vs. Controls |
| Liu D [42] | 36200542 | Changshu, China | ischemic stroke (ICD-10: codes I63) |
| Spasova N [71] | 38669041 | Sofia, Bulgaria | Carotid artery plaques, arterial stiffness [PWV, pulse wave velocity] |
| Huang S [72] | 37781984 | Singapore | acute myocardial infarction |

**Supplemental Table 9**. Cross-sectional studies that reported on the association between TMAO and severity of MACE.

| **1^st^ Author, year** | **PMID** | **Country** | **Outcome(s)** | **Sample size** | **High TMAO defined** | **Adjusted OR (95%CIs)** | **Co-variates** |
| --- | --- | --- | --- | --- | --- | --- | --- |
| Mafune A  2016 [120] | 26676906 | Tokyo, Japan | Number of infarcted coronary arteries on coronary angiography | n = 227 (130 no infarcted artery; 26 one artery; 20 two arteries; 50 three arteries) | Q1 1.0 (0.6-1.3); Q2 2.6 (2.0-2.8); Q3 4.4 (3.5-4.9); Q4 12.8 (8.6-17.2). Median (25-75^th^) µM | Q2 2.25 (0.83-6.15); Q3 3.35 (1.21-9.28); Q4 11.90 (3.88-36.7). | Age, sex, smoking, BMI, CKD stage, Diabetes, HbA1c, Insulin use, hypertension, dyslipidemia, congestive heart failure, use of beta blocker, cerebrovascular disease |
| Rexidamu M  2019 [89] | 31142624 | China, Shanghai | Severity of first ever acute ischemic stroke (defined as NIHSS score > 5 vs. mild stroke) | n = 255 (n = 138 mild stroke, 117 moderate/ severe stroke [n= 255 controls not used for data analyses!] | TMAO per unit increase in concentrations | 1.22 (1.08-1.32) | Age, sex, BMI, stroke, stroke etiology, vascular risk factors, pre-stroke treatment, lesion volumes and blood levels of TG, TC, HCY, Hs-CRP and fasting blood glucose |
| Wu c  2020 [121] | 31907287 | 4 stroke centers in China | Stroke severity in patients with acute stroke (NIHSS score <6 = mild; NIHSS score of ≥6 = moderate to severe) | n = 377 | Q1 < 3.3; q2 3.3-5.1; Q3 5.2-7.6; Q4 > 7.7 µmol/L | Q2 = 2.66(1.33–5.48), Q3 = 10.11 (5.56–23.41), Q4 = 24.78 (11.57–56.21) | Age, sex, hypertension, CAD, AF, smoking, PAD, syst BP, BMI, serum glucose, LDL, GFR, and time from stroke onset to MRI. |
|  |  |  | Infarct volume |  | Q1 < 2.9, Q2 29-5.1, Q3 5.2-8.1, Q4 > 8.1 µmol/L | Q2 3.11 (0.92–6.03), Q3 4.07 (2.81–5.54), Q4 4.36 (3.21–5.87) | Age, sex, hypertension, CVD, AF, smoking, PAD, blood pressure, BMI, serum glucose, LDL, GFR, MRI and time from stroke onset to MRI |
| Li Z  2023 [122] | 35020253 | Nanjing Medical University, China | Stroke severity among people with stroke and diabetes | n = 108 (n = 58 of the 108 with moderate to severe stroke) | > 3.24 vs. < 3.24 µM | 1.158 (1.006, 1.332) | Age, sex, hypertension, CAD, AF, smoking, systolic BP, stroke etiology, CRP, eGFR, platelet aggregation, LDL-C, fibrinogen, glucose, HbA1C, mean glucose, MAGE, BMI |
| Sheng Z  2019 [123] | 30594289 | China, Fuwai Hospital | SYNTAX score | n = 335 (unclear how was reached from 416 patients recruited ) | TMAO was a continuous variable; median (IQR) = 2.18 (1.34-3.90) µM | 1.16 (1.06-1.29) | Age, sex, TG, HDL, LDL, diabetes, hypertension, smoking, BMI, CRP, eGFR. |
|  |  |  | number of affected vessels (single vessel disease versus multi-vessel disease) |  | TMAO continuous | 1.15 (1.01-1.32) | Age, sex, TG, HDL, LDL, diabetes, hypertension, smoking, BMI, CRP, eGFR. |
| Tan y,  2019 [124] | 30599768 | Beijing, China | Plaque rupture (disrupted fibrous cap with a clear cavity formation) vs. plaque erosion (attached thrombus overlying an intact and visible plaque) | n = 146 | Continuous variable; median (IQR) = 2.28 (1.17–3.70) µM | Model 3 = 4.18 (2.51–6.97) | Age, sex, hypertension, diabetes mellitus, smoking, triglyceride, hsCRP and eGFR |
| Luciani M  2023 [33] | 36593094 | Switzerland, multicenter | The presence of MRI lesions (LNCCI, SNCI, MB) at baseline (yes vs. no) | n = 1722 with TMAO and MRI data | median (25-75^th^): T1 3.4 (2.8-4.0), T2 5.8 (5.2-6.6), T3 11.5 (9.1-16.1) µmol/L | for LNCCI presence (yes vs. no): T2 1.20 (0.88-1.64), T3 1.05 (0.74-1.48). SNCI presence (yes vs. no): T2, 0.84 (0.61-1.15), T3 1.61 (1.16-2.22). MB presence (yes vs. no): T2 1.17 (0.86-1.59), T3 1.15 (0.82-1.61) | Age, sex, BMI, smoking status, GFR, anticoagulants and antiplatelets, history of heart failure, diabetes, stroke or transient ischemic attack, coronary heart disease, and hypertension |
| Senthong V  2016 [125] | 27256833 | USA, Cleveland | SYNTAX Score (tertiles according to the SYNTAX trial-defined as: low (0 to 22), intermediate (23 to 32), and high (≥33) | n = 353 | unclear how TMAO was entered in the statistical model (ordinal logistic regression?) | 4.82 (2.43–9.57) | Age, sex, triglyceride, HDL cholesterol, LDL cholesterol, diabetes, hypertension, smoking, diffuse lesion, focal lesion, hsCRP, BMI, log eGFR, statins, ACEI or ARB, aspirin, and beta-blocker |
| Wang Z, 2011 [9] | 21475195 | USA, Cleveland | presence of CVD (defined as PAD or CAD) at blood collection | n = 1876 | TMAO quartiles (median (IQR) = 3.9 (2.6- 6.3)µM) | Q2 vs. Q1 = 1.56 (1.16-2.11), Q3 vs. Q1 = 1.50 (1.11-2.04); Q4 vs. Q1 = 3.06 (2.17-4.31) | Adjusted for age, sex, SBP, DBP, HDL, LDL, smoking, diabetes, medications (statin, angiotensin converting enzyme, aspirin, β-blocker), and estimated creatinine clearance |
| Huang PY,  2023 [126] | 37755998 | Hualian, Taiwan | Aortic stiffness measured by pressure applanation tonometry and defined as carotid–femoral pulse wave velocity > 10 m/s | n = 115 | per 1 µg/L | 1.010 (1.003-1.017) | Diabetes mellitus, hypertension, age, systolic blood pressure, glucose |
| Hobson S,  2023 [127] | 37548021 | Stockholm, Sweden | Coronary artery calcification defined as CAC >0 | n = 120 | TMAO highest tertile vs. Low + medium tertiles | 3.18 (1.12-9.05) | Age, sex, diabetes, hypertension, BMI, smoking, eGFR, LDL-C, left ventricular ejection fraction |
| He L,  2022 [128] | 36384389 | Peking, China | Severe vascular calcification (abdominal aortic calcification score > = 5.5) | n = 184 | per doubling of TMAO (for lnTMAO) | 1.263 (1.328–9.406) | Age, dialysis duration in months, diabetes, plasma PTH. |
| Hsu BG,  2022 [129] | 36006188 | Hualian, Taiwan | Peripheral arterial stiffness measured as Left and Right Brachial-Ankle PWV and defined as baPWVof >18m/s (left or right) to diagnose PAS | n = 157 | per 1 µg/L higher TMAO | 1.016 (1.002–1.029) | Diabetes, hypertension, age, systolic blood pressure, fasting glucose, eGFR, CRP |
| AF, arterial fibrillation; BMI, body mass index; CRP, C-reactive protein; eGFR, glomerular filtration rate; CAD, coronary artery disease; PTH, parat-hormone; Hcy, homocysteine; LDL, low density lipoprotein cholesterol; TG, triglycerides; IQR, interquartile range. | | | | | | | |

| **Supplemental Table 10.** Summary of common sources of systematic bias in the literature on TMAO and cardio-vascular diseases. | | |
| --- | --- | --- |
| **Source of bias** | **Evidence** | **Direction /Further explanation** |
| Publication bias | The meta-analyses on MACE and several related outcomes showed convincing evidence using Egger’s regression test and Funnel Plot that negative or null associations were less likely to be published. | Overestimation  due to unpublished negative results. We estimated roughly 30% of the studies may not have been published due to their negative results. |
| Confounding | Several confounders such as hypertension, renal function, age and sex are associated with TMAO and the clinical outcomes in the same time. We have clearly showed that the strength of the association estimates are roughly 30% lower when the adjusted HR (or OR in case control studies) are used in the meta-analyses. | Overestimation  Not adjusting for confounders or not adjusting for the appropriate confounders may overestimate the association. In subgroup analyses, we identified hypertension as a major confounder of the association under investigation here. |
| Selection bias | Almost all studies included people with established risk factors for the outcomes and having higher TMAO could be the results of having these risk factors. | Overestimation |
| High overall risk of bias | We found evidence that high risk of bias (especially due to generalizability or external validity) caused higher estimates of the associations between TMAO and diseases. | Overestimation  High risk of bias was responsible for overestimating the association and higher heterogeneity between the studies. |
| Overreporting/overlap of specific cohorts | Large cohorts contributed several times to the overall evidence. Studies with positive results on one outcome were likely to be published again on other related outcomes (stroke, MI, mortality, etc). Whereas, negative studies were unlikely to publish again. | Overestimation  due to overlap in the population and reporting several outcomes form the same cohort. |
| External validity of the exposure measurements | Different in-house LC-MS/MS methods were used without comparing between labs or using external quality control samples. Pre-analytical factors (e.g., fasting) and time of blood sampling in relation to the acute event are critical determinants of TMAO that are not standardized. TMAO concentrations were not comparable between the studies and did not show a dose-response relationship with log-HR of MACE or all-cause mortality. Also no dose-response relationship was found between higher TMAO in studies on renal patients versus studies on non-renal patients and higher pooled HR (95%CI) | Though the associations shown by the individual studies maybe internally valid, the generalizability in term of clinically relevant cut-off value or a threshold for TMAO that might be associated with the risk of diseases was not possible. |

| **Supplemental Table 11.** Comparison of results from the present meta-analysis with those of previous meta-analyses on the outcomes MACE and all-cause mortality. | | | | |
| --- | --- | --- | --- | --- |
| **Reference^2^** | **Study designs** | **Outcome** | **Number (n) of studies in meta- analysis^3^** | **Pooled effect size (95%CIs), adjusted for confounding and publication bias** |
| **Present study^4^** | CO | MACE | n = 34 | HR (95%CIs) = 1.07 (1.03-1.15); Knapp-Hartung adjustment 1.07 (0.96-1.20) |
|  | CC | MACE | n = 18 | OR (95%CIs) = 1.05 (0.94-1.18); after Knapp-Hartung adjustment 1.05 (0.79-1.40) |
|  | CO | All-cause mortality | n = 42 | HR (95%CIs) = 1.07 (1.003-1.15); after Knapp-Hartung adjustment = 1.07 (0.97-1.18) |
| Zhang H, 2024 [130] | CO, CC | Stroke | n = 5 | OR (95%CIs) = 1.83 (1.02, 3.29) |
|  | CO, CC | MACE | n = 5 | RR (95%CIs) ^1^ = 1.55 (1.17, 2.05) |
|  | CO, CC | All-cause mortality | n = 7 | HR (95%CIs) = 1.89 (1.15, 3.08) |
| Wang L, 2024 [131] | CO, CC | First stroke | n = 5 | OR (95%CIs) = 1.21 (1.09, 1.34) |
|  | CO, CC | Recurrent stroke | n = 4 (unclear, overlapping publications) | HR (95%CIs) = 1.17 (1.08, 1.27) |
| Chen P, 2022 [132] | CC, CO | Stroke | n = 5 | OR (95%CIs) = 1.64 (1.12, 2.41) |
| Farhangi MA, 2020 [133] | CC, nested CC, CS, CO | Stroke | n = 5 | OR (95%CIs) = 1.68 (0.86, 3.24) |
| Khan QA, 2024 [134] | CO | MACE and all-cause mortality combined | n = 31 (analyzed on the substudy level)^5^ | HR (95%CIs) = 1.41 (1.29, 1.54) |
|  | CO | All-cause mortality | n = 22 (analyzed on the substudy level) | HR (95%CIs) = 1.55 (1.37, 1.75) |
| Chen G, 2022 [135] | CO | All-cause mortality | n = 16 (analyzed on the substudy level?) | HR (95%CIs) = 1.56 (1.40, 1.75) |
|  | CO | CVD events and all-cause mortality combined | n = 17 (analyzed on the substudy level?) | HR (95%CIs) = 1.52 (1.38, 1.68) |
| Li X, 2022 [136] | CO | MACE | n = 3 (analyzed on the substudy level?) | RR (95%CIs) = 1.28 (1.17, 1.39) |
|  | CO | All-cause mortality | n = 10 (analyzed on the substudy level?) | RR (95%CIs) = 1.35 (1.28, 1.42) |
| Li W, 2020, China [137] | CO | MACE | n = 7 | HR (95%CIs) = 1.68 (1.44–1.96) |
|  | CO | All-cause mortality (adjusted for eGFR) | n = 5 | HR (95%CIs) = 1.23 (1.12-1.35) |
| Qi J, 2018 [138] | CO | CVD | n = 5 | HR (95%CIs) = 1.23 (1.07, 1.42) |
|  | CO | All-cause mortality | n = 10 | HR (95%CIs) = 1.55 (1.19, 2.02) |
| Farhangi MA, 2020 [139] | CO, CC, nested-CC, case-CO, CS | All-cause mortality | n = 15 (analyzed on the substudy level?) | HR (95%CIs) = 1.47 (1.29, 1.67) |
| Schiattarella GG, 2017 [140] | CO | All-cause mortality | n = 16 (analyzed on the substudy level) | HR (95%CIs) = 1.91 (1.40, 2.61) |
| ^1^ Risk Ratio (RR).  ^2^ None of the previous meta-analyses adjusted for publication bias. Adjustment for confounding in previous meta-analyses (using adjusted effect size) was often unclear or adjusted and unadjusted effect sizes were used interchangeably.  ^3^ From random effects model (if both are presented).  ^4^ We included independent populations whenever it was possible to judge the overlap. Overlapping publications were combined as one study. We run the meta-analyses on the study level, thus sub-studies were combined in one estimate before contributing to the meta-analysis. The study is the unit of the analysis in this case. The meta-analyses combined only adjusted effects size. We combined the studies on the same outcome only when they have the same statistical measure (HR or OR). Only data on directly reported effect size were combined (we did not compute effect size from number of participants in exposed and non-exposed groups).  ^5^ Running the meta-analysis on the subgroup level does not account for correlations within one study (e.g., because of sharing the same reference group). | | | | |

Reference List

1.	Senthong V, Wang Z, Li XS, Fan Y, Wu Y, Tang WH, Hazen SL (2016) Intestinal Microbiota-Generated Metabolite Trimethylamine-N-Oxide and 5-Year Mortality Risk in Stable Coronary Artery Disease: The Contributory Role of Intestinal Microbiota in a COURAGE-Like Patient Cohort. J Am Heart Assoc 5.

2.	Tang WH, Wang Z, Levison BS, Koeth RA, Britt EB, Fu X, Wu Y, Hazen SL (2013) Intestinal microbial metabolism of phosphatidylcholine and cardiovascular risk. N Engl J Med 368:1575-1584.

3.	Zhu W, Gregory JC, Org E, Buffa JA, Gupta N, Wang Z, Li L, Fu X, Wu Y, Mehrabian M et al. (2016) Gut Microbial Metabolite TMAO Enhances Platelet Hyperreactivity and Thrombosis Risk. Cell 165:111-124.

4.	Tang WH, Wang Z, Fan Y, Levison B, Hazen JE, Donahue LM, Wu Y, Hazen SL (2014) Prognostic value of elevated levels of intestinal microbe-generated metabolite trimethylamine-N-oxide in patients with heart failure: refining the gut hypothesis. J Am Coll Cardiol 64:1908-1914.

5.	Tang WH, Wang Z, Shrestha K, Borowski AG, Wu Y, Troughton RW, Klein AL, Hazen SL (2015) Intestinal microbiota-dependent phosphatidylcholine metabolites, diastolic dysfunction, and adverse clinical outcomes in chronic systolic heart failure. J Card Fail 21:91-96.

6.	Tang WH, Wang Z, Kennedy DJ, Wu Y, Buffa JA, Agatisa-Boyle B, Li XS, Levison BS, Hazen SL (2015) Gut microbiota-dependent trimethylamine N-oxide (TMAO) pathway contributes to both development of renal insufficiency and mortality risk in chronic kidney disease. Circ Res 116:448-455.

7.	Senthong V, Wang Z, Fan Y, Wu Y, Hazen SL, Tang WH (2016) Trimethylamine N-Oxide and Mortality Risk in Patients With Peripheral Artery Disease. J Am Heart Assoc 5.

8.	Tang WH, Wang Z, Li XS, Fan Y, Li DS, Wu Y, Hazen SL (2017) Increased Trimethylamine N-Oxide Portends High Mortality Risk Independent of Glycemic Control in Patients with Type 2 Diabetes Mellitus. Clin Chem 63:297-306.

9.	Wang Z, Klipfell E, Bennett BJ, Koeth R, Levison BS, Dugar B, Feldstein AE, Britt EB, Fu X, Chung YM et al. (2011) Gut flora metabolism of phosphatidylcholine promotes cardiovascular disease. Nature 472:57-63.

10.	Li XS, Obeid S, Klingenberg R, Gencer B, Mach F, Raber L, Windecker S, Rodondi N, Nanchen D, Muller O et al. (2017) Gut microbiota-dependent trimethylamine N-oxide in acute coronary syndromes: a prognostic marker for incident cardiovascular events beyond traditional risk factors. Eur Heart J 38:814-824.

11.	Kaysen GA, Johansen KL, Chertow GM, Dalrymple LS, Kornak J, Grimes B, Dwyer T, Chassy AW, Fiehn O (2015) Associations of Trimethylamine N-Oxide With Nutritional and Inflammatory Biomarkers and Cardiovascular Outcomes in Patients New to Dialysis. J Ren Nutr 25:351-356.

12.	Shafi T, Powe NR, Meyer TW, Hwang S, Hai X, Melamed ML, Banerjee T, Coresh J, Hostetter TH (2017) Trimethylamine N-Oxide and Cardiovascular Events in Hemodialysis Patients. J Am Soc Nephrol 28:321-331.

13.	Stubbs JR, Stedman MR, Liu S, Long J, Franchetti Y, West RE, III, Prokopienko AJ, Mahnken JD, Chertow GM, Nolin TD (2019) Trimethylamine N-Oxide and Cardiovascular Outcomes in Patients with ESKD Receiving Maintenance Hemodialysis. Clin J Am Soc Nephrol 14:261-267.

14.	Heianza Y, Ma W, DiDonato JA, Sun Q, Rimm EB, Hu FB, Rexrode KM, Manson JE, Qi L (2020) Long-Term Changes in Gut Microbial Metabolite Trimethylamine N-Oxide and Coronary Heart Disease Risk. J Am Coll Cardiol 75:763-772.

15.	Liu G, Li J, Li Y, Hu Y, Franke AA, Liang L, Hu FB, Chan AT, Mukamal KJ, Rimm EB et al. (2021) Gut microbiota-derived metabolites and risk of coronary artery disease: a prospective study among US men and women. Am J Clin Nutr 114:238-247.

16.	Miller PE, Haberlen SA, Brown TT, Margolick JB, DiDonato JA, Hazen SL, Witt MD, Kingsley LA, Palella FJ, Jr., Budoff M et al. (2016) Brief Report: Intestinal Microbiota-Produced Trimethylamine-N-Oxide and Its Association With Coronary Stenosis and HIV Serostatus. J Acquir Immune Defic Syndr 72:114-118.

17.	Meyer KA, Benton TZ, Bennett BJ, Jacobs DR, Jr., Lloyd-Jones DM, Gross MD, Carr JJ, Gordon-Larsen P, Zeisel SH (2016) Microbiota-Dependent Metabolite Trimethylamine N-Oxide and Coronary Artery Calcium in the Coronary Artery Risk Development in Young Adults Study (CARDIA). J Am Heart Assoc 5.

18.	Shea JW, Jacobs DR, Jr., Howard AG, Lulla A, Lloyd-Jones DM, Murthy VL, Shah RV, Trujillo-Gonzalez I, Gordon-Larsen P, Meyer KA (2024) Choline metabolites and incident cardiovascular disease in a prospective cohort of adults: Coronary Artery Risk Development in Young Adults (CARDIA) Study. Am J Clin Nutr 119:29-38.

19.	Lee Y, Nemet I, Wang Z, Lai HTM, de Oliveira Otto MC, Lemaitre RN, Fretts AM, Sotoodehnia N, Budoff M, DiDonato JA et al. (2021) Longitudinal Plasma Measures of Trimethylamine N-Oxide and Risk of Atherosclerotic Cardiovascular Disease Events in Community-Based Older Adults. J Am Heart Assoc 10:e020646.

20.	Sapa H, Gutierrez OM, Shlipak MG, Katz R, Ix JH, Sarnak MJ, Cushman M, Rhee EP, Kimmel PL, Vasan RS et al. (2022) Association of Uremic Solutes With Cardiovascular Death in Diabetic Kidney Disease. Am J Kidney Dis 80:502-512.

21.	Fretts AM, Hazen SL, Jensen P, Budoff M, Sitlani CM, Wang M, de Oliveira Otto MC, DiDonato JA, Lee Y, Psaty BM et al. (2022) Association of Trimethylamine N-Oxide and Metabolites With Mortality in Older Adults. JAMA Netw Open 5:e2213242.

22.	Stubbs JR, House JA, Ocque AJ, Zhang S, Johnson C, Kimber C, Schmidt K, Gupta A, Wetmore JB, Nolin TD et al. (2016) Serum Trimethylamine-N-Oxide is Elevated in CKD and Correlates with Coronary Atherosclerosis Burden. J Am Soc Nephrol 27:305-313.

23.	Wang M, Tang WHW, Li XS, de Oliveira Otto MC, Lee Y, Lemaitre RN, Fretts A, Nemet I, Sotoodehnia N, Sitlani CM et al. (2024) The Gut Microbial Metabolite Trimethylamine N -oxide, Incident CKD, and Kidney Function Decline. J Am Soc Nephrol 35:749-760.

24.	Lemaitre RN, Jensen PN, Wang Z, Fretts AM, Sitlani CM, Nemet I, Sotoodehnia N, de Oliveira Otto MC, Zhu W, Budoff M et al. (2023) Plasma Trimethylamine-N-Oxide and Incident Ischemic Stroke: The Cardiovascular Health Study and the Multi-Ethnic Study of Atherosclerosis. J Am Heart Assoc 12:e8711.

25.	Tang WHW, Lemaitre RN, Jensen PN, Wang M, Wang Z, Li XS, Nemet I, Lee Y, Lai HTM, de Oliveira Otto MC et al. (2024) Trimethylamine N-Oxide and Related Gut Microbe-Derived Metabolites and Incident Heart Failure Development in Community-Based Populations. Circ Heart Fail 17:e011569.

26.	Cardona A, O'Brien A, Bernier MC, Somogyi A, Wysocki VH, Smart S, He X, Ambrosio G, Hsueh WA, Raman SV (2019) Trimethylamine N-oxide and incident atherosclerotic events in high-risk individuals with diabetes: an ACCORD trial post hoc analysis. BMJ Open Diabetes Res Care 7:e000718.

27.	Mente A, Chalcraft K, Ak H, Davis AD, Lonn E, Miller R, Potter MA, Yusuf S, Anand SS, McQueen MJ (2015) The Relationship Between Trimethylamine-N-Oxide and Prevalent Cardiovascular Disease in a Multiethnic Population Living in Canada. Can J Cardiol 31:1189-1194.

28.	Kim RB, Morse BL, Djurdjev O, Tang M, Muirhead N, Barrett B, Holmes DT, Madore F, Clase CM, Rigatto C et al. (2016) Advanced chronic kidney disease populations have elevated trimethylamine N-oxide levels associated with increased cardiovascular events. Kidney Int 89:1144-1152.

29.	Reiner MF, Muller D, Gobbato S, Stalder O, Limacher A, Bonetti NR, Pasterk L, Mean M, Rodondi N, Aujesky D et al. (2019) Gut microbiota-dependent trimethylamine-N-oxide (TMAO) shows a U-shaped association with mortality but not with recurrent venous thromboembolism. Thromb Res 174:40-47.

30.	Ottiger M, Nickler M, Steuer C, Odermatt J, Huber A, Christ-Crain M, Henzen C, Hoess C, Thomann R, Zimmerli W et al. (2016) Trimethylamine-N-oxide (TMAO) predicts fatal outcomes in community-acquired pneumonia patients without evident coronary artery disease. Eur J Intern Med 36:67-73.

31.	Hochstrasser SR, Metzger K, Vincent AM, Becker C, Keller AKJ, Beck K, Perrig S, Tisljar K, Sutter R, Schuetz P et al. (2020) Trimethylamine-N-oxide (TMAO) predicts short- and long-term mortality and poor neurological outcome in out-of-hospital cardiac arrest patients. Clin Chem Lab Med 59:393-402.

32.	Amrein M, Li XS, Walter J, Wang Z, Zimmermann T, Strebel I, Honegger U, Leu K, Schafer I, Twerenbold R et al. (2022) Gut microbiota-dependent metabolite trimethylamine N-oxide (TMAO) and cardiovascular risk in patients with suspected functionally relevant coronary artery disease (fCAD). Clin Res Cardiol 111:692-704.

33.	Luciani M, Muller D, Vanetta C, Diteepeng T, von EA, Aeschbacher S, Rodondi N, Moschovitis G, Reichlin T, Sinnecker T et al. (2023) Trimethylamine-N-oxide is associated with cardiovascular mortality and vascular brain lesions in patients with atrial fibrillation. Heart 109:396-404.

34.	Davies A, Wenzl FA, Li XS, Winzap P, Obeid S, Klingenberg R, Mach F, Raber L, Muller O, Matter CM et al. (2023) Short and medium chain acylcarnitines as markers of outcome in diabetic and non-diabetic subjects with acute coronary syndromes. Int J Cardiol 389:131261.

35.	Xu J, Cheng A, Song B, Zhao M, Xue J, Wang A, Dai L, Jing J, Meng X, Li H et al. (2022) Trimethylamine N-Oxide and Stroke Recurrence Depends on Ischemic Stroke Subtypes. Stroke 53:1207-1215.

36.	Xu J, Zhao M, Wang A, Xue J, Cheng S, Cheng A, Gao J, Zhang Q, Zhan R, Meng X et al. (2021) Association Between Plasma Trimethyllysine and Prognosis of Patients With Ischemic Stroke. J Am Heart Assoc 10:e020979.

37.	Xu J, Mo J, Zhang X, Chen Z, Pan Y, Yan H, Meng X, Wang Y (2022) Nontraditional Risk Factors for Residual Recurrence Risk in Patients with Ischemic Stroke of Different Etiologies. Cerebrovasc Dis 51:630-638.

38.	Xue J, Xu J, Zhao M, Jin A, Cheng A, Jiang X, Li K, Lin J, Meng X, Li H et al. (2022) Residual Risk of Trimethylamine-N-Oxide and Choline for Stroke Recurrence in Patients With Intensive Secondary Therapy. J Am Heart Assoc 11:e027265.

39.	Waleed KB, Tse G, Lu YK, Peng CN, Tu H, Ding LG, Xia YL, Wu SL, Li XT, Zhou HQ et al. (2021) Trimethylamine N-oxide is associated with coronary atherosclerotic burden in non-ST-segment myocardial infarction patients: SZ-NSTEMI prospective cohort study. Rev Cardiovasc Med 22:231-238.

40.	Li J, Sheng Z, Tan Y, Zhou P, Liu C, Zhao H, Song L, Zhou J, Chen R, Chen Y et al. (2021) Association of plasma trimethylamine N-Oxide level with healed culprit plaques examined by optical coherence tomography in patients with ST-Segment elevation myocardial infarction. Nutr Metab Cardiovasc Dis 31:145-152.

41.	Zhao X, Zhao H, Chen R, Li J, Zhou J, Li N, Yan S, Liu C, Zhou P, Chen Y et al. (2024) A Combined Measure of the Triglyceride Glucose Index and Trimethylamine N-Oxide in Risk Stratification of ST-Segment Elevation Myocardial Infarction Patients with High-Risk Plaque Features Defined by Optical Coherence Tomography: A Substudy of the OCTAMI Registry Study. Vasc Health Risk Manag 20:141-155.

42.	Liu D, Gu S, Zhou Z, Ma Z, Zuo H (2023) Associations of plasma TMAO and its precursors with stroke risk in the general population: A nested case-control study. J Intern Med 293:110-120.

43.	Yu X, Wang Y, Yang R, Wang Z, Wang X, Wang S, Zhang W, Dong J, Chen W, Ji F et al. (2024) Trimethylamine N-oxide predicts cardiovascular events in coronary artery disease patients with diabetes mellitus: a prospective cohort study. Front Endocrinol (Lausanne) 15:1360861.

44.	Nie J, Xie L, Zhao BX, Li Y, Qiu B, Zhu F, Li GF, He M, Wang Y, Wang B et al. (2018) Serum Trimethylamine N-Oxide Concentration Is Positively Associated With First Stroke in Hypertensive Patients. Stroke 49:2021-2028.

45.	Flores-Guerrero JL, van Dijk PR, Connelly MA, Garcia E, Bilo HJG, Navis G, Bakker SJL, Dullaart RPF (2021) Circulating Trimethylamine N-Oxide Is Associated with Increased Risk of Cardiovascular Mortality in Type-2 Diabetes: Results from a Dutch Diabetes Cohort (ZODIAC-59). J Clin Med 10.

46.	Flores-Guerrero JL, Post A, van Dijk PR, Connelly MA, Garcia E, Navis G, Bakker SJL, Dullaart RPF (2021) Circulating trimethylamine-N-oxide is associated with all-cause mortality in subjects with nonalcoholic fatty liver disease. Liver Int 41:2371-2382.

47.	Gruppen EG, Garcia E, Connelly MA, Jeyarajah EJ, Otvos JD, Bakker SJL, Dullaart RPF (2017) TMAO is Associated with Mortality: Impact of Modestly Impaired Renal Function. Sci Rep 7:13781.

48.	Tang WHW, Li XS, Wu Y, Wang Z, Khaw KT, Wareham NJ, Nieuwdorp M, Boekholdt SM, Hazen SL (2021) Plasma trimethylamine N-oxide (TMAO) levels predict future risk of coronary artery disease in apparently healthy individuals in the EPIC-Norfolk prospective population study. Am Heart J 236:80-86.

49.	Svingen GFT, Zuo H, Ueland PM, Seifert R, Loland KH, Pedersen ER, Schuster PM, Karlsson T, Tell GS, Schartum-Hansen H et al. (2018) Increased plasma trimethylamine-N-oxide is associated with incident atrial fibrillation. Int J Cardiol 267:100-106.

50.	Winther SA, Ollgaard JC, Tofte N, Tarnow L, Wang Z, Ahluwalia TS, Jorsal A, Theilade S, Parving HH, Hansen TW et al. (2019) Utility of Plasma Concentration of Trimethylamine N-Oxide in Predicting Cardiovascular and Renal Complications in Individuals With Type 1 Diabetes. Diabetes Care 42:1512-1520.

51.	Winther SA, Ollgaard JC, Hansen TW, von Scholten BJ, Reinhard H, Ahluwalia TS, Wang Z, Gaede P, Parving HH, Hazen S et al. (2021) Plasma trimethylamine N-oxide and its metabolic precursors and risk of mortality, cardiovascular and renal disease in individuals with type 2-diabetes and albuminuria. PLoS ONE 16:e0244402.

52.	Bjornestad EO, Dhar I, Svingen GFT, Pedersen ER, Orn S, Svenningsson MM, Tell GS, Ueland PM, Sulo G, Laaksonen R et al. (2022) Circulating trimethylamine N-oxide levels do not predict 10-year survival in patients with or without coronary heart disease. J Intern Med 292:915-924.

53.	Hernandez L, Ward LJ, Arefin S, Ebert T, Laucyte-Cibulskiene A, Heimburger O, Barany P, Wennberg L, Stenvinkel P, Kublickiene K (2022) Blood-brain barrier and gut barrier dysfunction in chronic kidney disease with a focus on circulating biomarkers and tight junction proteins. Sci Rep 12:4414.

54.	Mueller DM, Allenspach M, Othman A, Saely CH, Muendlein A, Vonbank A, Drexel H, von EA (2015) Plasma levels of trimethylamine-N-oxide are confounded by impaired kidney function and poor metabolic control. Atherosclerosis 243:638-644.

55.	Schuett K, Kleber ME, Scharnagl H, Lorkowski S, Marz W, Niessner A, Marx N, Meinitzer A (2017) Trimethylamine-N-oxide and Heart Failure With Reduced Versus Preserved Ejection Fraction. J Am Coll Cardiol 70:3202-3204.

56.	Berger M, Kleber ME, Delgado GE, Marz W, Andreas M, Hellstern P, Marx N, Schuett KA (2020) Trimethylamine N-Oxide and Adenosine Diphosphate-Induced Platelet Reactivity Are Independent Risk Factors for Cardiovascular and All-Cause Mortality. Circ Res 126:660-662.

57.	Obeid R, Awwad H, Heine GH, Emrich IE, Fliser D, Zawada AM, Geisel J (2024) Plasma Concentrations of Trimethylamine-N-Oxide, Choline, and Betaine in Patients With Moderate to Advanced Chronic Kidney Disease and Their Relation to Cardiovascular and Renal Outcomes. J Ren Nutr .

58.	Haghikia A, Li XS, Liman TG, Bledau N, Schmidt D, Zimmermann F, Krankel N, Widera C, Sonnenschein K, Haghikia A et al. (2018) Gut Microbiota-Dependent Trimethylamine N-Oxide Predicts Risk of Cardiovascular Events in Patients With Stroke and Is Related to Proinflammatory Monocytes. Arterioscler Thromb Vasc Biol 38:2225-2235.

59.	Guasch-Ferre M, Hu FB, Ruiz-Canela M, Bullo M, Toledo E, Wang DD, Corella D, Gomez-Gracia E, Fiol M, Estruch R et al. (2017) Plasma Metabolites From Choline Pathway and Risk of Cardiovascular Disease in the PREDIMED (Prevention With Mediterranean Diet) Study. J Am Heart Assoc 6.

60.	Papandreou C, Bullo M, Hernandez-Alonso P, Ruiz-Canela M, Li J, Guasch-Ferre M, Toledo E, Clish C, Corella D, Estruch R et al. (2021) Choline Metabolism and Risk of Atrial Fibrillation and Heart Failure in the PREDIMED Study. Clin Chem 67:288-297.

61.	Dai L, Massy ZA, Stenvinkel P, Chesnaye NC, Larabi IA, Alvarez JC, Caskey FJ, Torino C, Porto G, Szymczak M et al. (2022) The association between TMAO, CMPF, and clinical outcomes in advanced chronic kidney disease: results from the European QUALity (EQUAL) Study. Am J Clin Nutr 116:1842-1851.

62.	Wargny M, Croyal M, Ragot S, Gand E, Jacobi D, Trochu JN, Prieur X, Le MC, Goronflot T, Cariou B et al. (2022) Nutritional biomarkers and heart failure requiring hospitalization in patients with type 2 diabetes: the SURDIAGENE cohort. Cardiovasc Diabetol 21:101.

63.	Croyal M, Saulnier PJ, Aguesse A, Gand E, Ragot S, Roussel R, Halimi JM, Ducrocq G, Cariou B, Montaigne D et al. (2020) Plasma Trimethylamine N-Oxide and Risk of Cardiovascular Events in Patients With Type 2 Diabetes. J Clin Endocrinol Metab 105.

64.	Ringel C, Dittrich J, Gaudl A, Schellong P, Beuchel CF, Baber R, Beutner F, Teren A, Engel C, Wirkner K et al. (2021) Association of plasma trimethylamine N-oxide levels with atherosclerotic cardiovascular disease and factors of the metabolic syndrome. Atherosclerosis 335:62-67.

65.	Suzuki T, Yazaki Y, Voors AA, Jones DJL, Chan DCS, Anker SD, Cleland JG, Dickstein K, Filippatos G, Hillege HL et al. (2019) Association with outcomes and response to treatment of trimethylamine N-oxide in heart failure: results from BIOSTAT-CHF. Eur J Heart Fail 21:877-886.

66.	Gencer B, Li XS, Gurmu Y, Bonaca MP, Morrow DA, Cohen M, Bhatt DL, Steg PG, Storey RF, Johanson P et al. (2020) Gut Microbiota-Dependent Trimethylamine N-oxide and Cardiovascular Outcomes in Patients With Prior Myocardial Infarction: A Nested Case Control Study From the PEGASUS-TIMI 54 Trial. J Am Heart Assoc 9:e015331.

67.	Bhuiya J, Notsu Y, Kobayashi H, Shibly AZ, Sheikh AM, Okazaki R, Yamaguchi K, Nagai A, Nabika T, Abe T et al. (2023) Neither Trimethylamine-N-Oxide nor Trimethyllysine Is Associated with Atherosclerosis: A Cross-Sectional Study in Older Japanese Adults. Nutrients 15.

68.	Senthong V, Kiatchoosakun S, Wongvipaporn C, Phetcharaburanin J, Tatsanavivat P, Sritara P, Phrommintikul A (2021) Gut microbiota-generated metabolite, trimethylamine-N-oxide, and subclinical myocardial damage: a multicenter study from Thailand. Sci Rep 11:14963.

69.	Senthong V, Kiatchoosakun S, Wongvipaporn C, Phetcharaburanin J, Sritara P, Phrommintikul A (2024) Trimethylamine-N-oxide and 5-year mortality: the role of gut microbiota-generated metabolite from the CORE-Thailand cohort. Sci Rep 14:21264.

70.	Lever M, George PM, Slow S, Bellamy D, Young JM, Ho M, McEntyre CJ, Elmslie JL, Atkinson W, Molyneux SL et al. (2014) Betaine and Trimethylamine-N-Oxide as Predictors of Cardiovascular Outcomes Show Different Patterns in Diabetes Mellitus: An Observational Study. PLoS ONE 9:e114969.

71.	Spasova N, Somleva D, Krastev B, Ilieva R, Borizanova A, Svinarov D, Kinova E, Goudev A (2024) Association of the trimethylamine N-oxide with cardiovascular risk and vascular alterations in middle-aged patients with risk factors for cardiovascular diseases. Biosci Rep 44.

72.	Huang S, Lim SY, Tan SH, Chan MY, Ni W, Li SFY (2023) Targeted Plasma Metabolomics Reveals Association of Acute Myocardial Infarction Risk with the Dynamic Balance between Trimethylamine-N-oxide, Betaine, and Choline. J Agric Food Chem 71:15097-15105.

73.	Chen YY, Ye ZS, Xia NG, Xu Y (2022) TMAO as a Novel Predictor of Major Adverse Vascular Events and Recurrence in Patients with Large Artery Atherosclerotic Ischemic Stroke. Clin Appl Thromb Hemost 28:10760296221090503.

74.	Xu D, Zhao W, Song J, Yin L, Wang K, Wei L, Xu Y, Li H, Min B, Tang N et al. (2021) The Relationship of Large-Artery Atherothrombotic Stroke with Plasma Trimethylamine N-Oxide Level and Blood Lipid-Related Indices: A Cross-Sectional Comparative Study. Biomed Res Int 2021:5549796.

75.	Guo F, Zhou J, Li Z, Yu Z, Ouyang D (2020) The Association between Trimethylamine N-Oxide and Its Predecessors Choline, L-Carnitine, and Betaine with Coronary Artery Disease and Artery Stenosis. Cardiol Res Pract 2020:5854919.

76.	Sun T, Zhang Y, Yin J, Peng X, Zhou L, Huang S, Wen Y, Cao B, Chen L, Li X et al. (2021) Association of Gut Microbiota-Dependent Metabolite Trimethylamine N-Oxide with First Ischemic Stroke. J Atheroscler Thromb 28:320-328.

77.	Schneider C, Okun JG, Schwarz KV, Hauke J, Zorn M, Nurnberg C, Ungerer M, Ringleb PA, Mundiyanapurath S (2020) Trimethylamine-N-oxide is elevated in the acute phase after ischaemic stroke and decreases within the first days. Eur J Neurol 27:1596-1603.

78.	Zheng L, Zheng J, Xie Y, Li Z, Guo X, Sun G, Sun Z, Xing F, Sun Y (2019) Serum gut microbe-dependent trimethylamine N-oxide improves the prediction of future cardiovascular disease in a community-based general population. Atherosclerosis 280:126-131.

79.	Liang Z, Dong Z, Guo M, Shen Z, Yin D, Hu S, Hai X (2019) Trimethylamine N-oxide as a risk marker for ischemic stroke in patients with atrial fibrillation. J Biochem Mol Toxicol 33:e22246.

80.	Dong Z, Liang Z, Guo M, Hu S, Shen Z, Hai X (2018) The Association between Plasma Levels of Trimethylamine N-Oxide and the Risk of Coronary Heart Disease in Chinese Patients with or without Type 2 Diabetes Mellitus. Dis Markers 2018:1578320.

81.	Troseid M, Ueland T, Hov JR, Svardal A, Gregersen I, Dahl CP, Aakhus S, Gude E, Bjorndal B, Halvorsen B et al. (2015) Microbiota-dependent metabolite trimethylamine-N-oxide is associated with disease severity and survival of patients with chronic heart failure. J Intern Med 277:717-726.

82.	Missailidis C, Hallqvist J, Qureshi AR, Barany P, Heimburger O, Lindholm B, Stenvinkel P, Bergman P (2016) Serum Trimethylamine-N-Oxide Is Strongly Related to Renal Function and Predicts Outcome in Chronic Kidney Disease. PLoS ONE 11:e0141738.

83.	Skagen K, Troseid M, Ueland T, Holm S, Abbas A, Gregersen I, Kummen M, Bjerkeli V, Reier-Nilsen F, Russell D et al. (2016) The Carnitine-butyrobetaine-trimethylamine-N-oxide pathway and its association with cardiovascular mortality in patients with carotid atherosclerosis. Atherosclerosis 247:64-69.

84.	Suzuki T, Heaney LM, Bhandari SS, Jones DJ, Ng LL (2016) Trimethylamine N-oxide and prognosis in acute heart failure. Heart 102:841-848.

85.	Robinson-Cohen C, Newitt R, Shen DD, Rettie AE, Kestenbaum BR, Himmelfarb J, Yeung CK (2016) Association of FMO3 Variants and Trimethylamine N-Oxide Concentration, Disease Progression, and Mortality in CKD Patients. PLoS ONE 11:e0161074.

86.	Suzuki T, Heaney LM, Jones DJ, Ng LL (2017) Trimethylamine N-oxide and Risk Stratification after Acute Myocardial Infarction. Clin Chem 63:420-428.

87.	Li XS, Obeid S, Klingenberg R, Gencer B, Mach F, Raber L, Windecker S, Rodondi N, Nanchen D, Muller O et al. (2017) Gut microbiota-dependent trimethylamine N-oxide in acute coronary syndromes: a prognostic marker for incident cardiovascular events beyond traditional risk factors. Eur Heart J 38:814-824.

88.	Wu C, Li C, Zhao W, Xie N, Yan F, Lian Y, Zhou L, Xu X, Liang Y, Wang L et al. (2018) Elevated trimethylamine N-oxide related to ischemic brain lesions after carotid artery stenting. Neurology 90:e1283-e1290.

89.	Rexidamu M, Li H, Jin H, Huang J (2019) Serum levels of Trimethylamine-N-oxide in patients with ischemic stroke. Biosci Rep 39.

90.	Zhai Q, Wang X, Chen C, Tang Y, Wang Y, Tian J, Zhao Y, Liu X (2019) Prognostic Value of Plasma Trimethylamine N-Oxide Levels in Patients with Acute Ischemic Stroke. Cell Mol Neurobiol 39:1201-1206.

91.	Nam HS, Ha J, Ji D, Kwon I, Lee HS, Han M, Kim YD, Heo JH, Lee SG (2019) Elevation of the Gut Microbiota Metabolite Trimethylamine N-Oxide Predicts Stroke Outcome. J Stroke 21:350-352.

92.	Roncal C, Martinez-Aguilar E, Orbe J, Ravassa S, Fernandez-Montero A, Saenz-Pipaon G, Ugarte A, Estella-Hermoso de MA, Rodriguez JA, Fernandez-Alonso S et al. (2019) Trimethylamine-N-Oxide (TMAO) Predicts Cardiovascular Mortality in Peripheral Artery Disease. Sci Rep 9:15580.

93.	Hou L, Zhang Y, Zheng D, Shi H, Zou C, Zhang H, Lu Z, Du H (2020) Increasing trimethylamine N-oxide levels as a predictor of early neurological deterioration in patients with acute ischemic stroke. Neurol Res 42:153-158.

94.	Zhou X, Jin M, Liu L, Yu Z, Lu X, Zhang H (2020) Trimethylamine N-oxide and cardiovascular outcomes in patients with chronic heart failure after myocardial infarction. ESC Heart Fail 7:188-193.

95.	Tan C, Wang H, Gao X, Xu R, Zeng X, Cui Z, Zhu J, Wu Q, Xia G, Zhou H et al. (2020) Dynamic Changes and Prognostic Value of Gut Microbiota-Dependent Trimethylamine-N-Oxide in Acute Ischemic Stroke. Front Neurol 11:29.

96.	Zhang P, Zou JZ, Chen J, Tan X, Xiang FF, Shen B, Hu JC, Wang JL, Wang YQ, Yu JB et al. (2020) Association of trimethylamine N-Oxide with cardiovascular and all-cause mortality in hemodialysis patients. Ren Fail 42:1004-1014.

97.	Israr MZ, Bernieh D, Salzano A, Cassambai S, Yazaki Y, Heaney LM, Jones DJL, Ng LL, Suzuki T (2021) Association of gut-related metabolites with outcome in acute heart failure. Am Heart J 234:71-80.

98.	Fu D, Shen J, Li W, Wang Y, Zhong Z, Ye H, Huang N, Fan L, Yang X, Yu X et al. (2021) Elevated Serum Trimethylamine N-Oxide Levels Are Associated with Mortality in Male Patients on Peritoneal Dialysis. Blood Purif 50:837-847.

99.	Zhang J, Wang L, Cai J, Lei A, Liu C, Lin R, Jia L, Fu Y (2021) Gut microbial metabolite TMAO portends prognosis in acute ischemic stroke. J Neuroimmunol 354:577526.

100.	Kinugasa Y, Nakamura K, Kamitani H, Hirai M, Yanagihara K, Kato M, Yamamoto K (2021) Trimethylamine N-oxide and outcomes in patients hospitalized with acute heart failure and preserved ejection fraction. ESC Heart Fail 8:2103-2110.

101.	Wei H, Zhao M, Huang M, Li C, Gao J, Yu T, Zhang Q, Shen X, Ji L, Ni L et al. (2022) FMO3-TMAO axis modulates the clinical outcome in chronic heart-failure patients with reduced ejection fraction: evidence from an Asian population. Front Med 16:295-305.

102.	Chang D, Xu X, Yang Z, Ma T, Nie J, Dong J (2022) Trimethylamine-N-oxide (TMAO) and clinical outcomes in patients with end-stage kidney disease receiving peritoneal dialysis. Perit Dial Int 42:622-630.

103.	Eyileten C, Jarosz-Popek J, Jakubik D, Gasecka A, Wolska M, Ufnal M, Postula M, Toma A, Lang IM, Siller-Matula JM (2021) Plasma Trimethylamine-N-Oxide Is an Independent Predictor of Long-Term Cardiovascular Mortality in Patients Undergoing Percutaneous Coronary Intervention for Acute Coronary Syndrome. Front Cardiovasc Med 8:728724.

104.	Li N, Zhou J, Wang Y, Chen R, Li J, Zhao X, Zhou P, Liu C, Song L, Liao Z et al. (2022) Association between trimethylamine N-oxide and prognosis of patients with acute myocardial infarction and heart failure. ESC Heart Fail 9:3846-3857.

105.	Matsuzawa Y, Nakahashi H, Konishi M, Sato R, Kawashima C, Kikuchi S, Akiyama E, Iwahashi N, Maejima N, Okada K et al. (2019) Microbiota-derived Trimethylamine N-oxide Predicts Cardiovascular Risk After STEMI. Sci Rep 9:11647.

106.	Li N, Wang Y, Zhou J, Chen R, Li J, Zhao X, Zhou P, Liu C, Chen Y, Song L et al. (2022) Association between the Changes in Trimethylamine N-Oxide-Related Metabolites and Prognosis of Patients with Acute Myocardial Infarction: A Prospective Study. J Cardiovasc Dev Dis 9.

107.	Sanchez-Gimenez R, Peiro OM, Bonet G, Carrasquer A, Fragkiadakis GA, Bullo M, Papandreou C, Bardaji A (2022) Plasma trimethylamine-N-oxide, its precursors and risk of cardiovascular events in patients with acute coronary syndrome: Mediating effects of renal function. Front Cardiovasc Med 9:1000815.

108.	Meng S, Ni T, Du Q, Liu M, Ge P, Geng J, Wang B (2024) Pre-procedural TMAO as a predictor for recurrence of atrial fibrillation after catheter ablation. BMC Cardiovasc Disord 24:750.

109.	Cheng E, Hung SC, Lin TY (2025) Association of trimethylamine N-oxide and metabolites with kidney function decline in patients with chronic kidney disease. Clin Nutr 44:239-247.

110.	Aldujeli A, Tsai TY, Haq A, Tatarunas V, Garg S, Hughes D, Ciapiene I, Unikas R, Sharif F, Lesauskaite V et al. (2024) The association between trimethylamine N-oxide levels and coronary microvascular dysfunction and prognosis in patients with ST-elevation myocardial infarction. Atherosclerosis 398:118597.

111.	Yu PS, Wu PH, Hung WW, Lin MY, Zhen YY, Hung WC, Chang JM, Tsai JR, Chiu YW, Hwang SJ et al. (2024) Association Between Trimethylamine N-oxide and Adverse Kidney Outcomes and Overall Mortality in Type 2 Diabetes Mellitus. J Clin Endocrinol Metab 109:2097-2105.

112.	Zhao S, Tian Y, Wang S, Yang F, Xu J, Qin Z, Liu X, Cao M, Zhao P, Zhang G et al. (2023) Prognostic value of gut microbiota-derived metabolites in patients with ST-segment elevation myocardial infarction. Am J Clin Nutr 117:499-508.

113.	Yuan X, Wei X, Liu J, Tang C, Peng L, Wang H, Huang Y (2023) Association of Circulating Trimethylamine-N Oxide With Malnutrition and the Risk of Coronary Artery Disease in Patients With Maintenance Hemodialysis. J Ren Nutr 33:465-471.

114.	Konieczny R, Zurawska-Plaksej E, Kaaz K, Czapor-Irzabek H, Bombala W, Mysiak A, Kuliczkowski W (2022) All-Cause Mortality and Trimethylamine N-Oxide Levels in Patients with Cardiovascular Disease. Cardiology 147:443-452.

115.	Zhai Q, Sun T, Sun C, Yan L, Wang X, Wang Y, Sun J, Zhao Y (2021) High plasma levels of trimethylamine N-oxide are associated with poor outcome in intracerebral hemorrhage patients. Neurol Sci 42:1009-1016.

116.	Mente A, Chalcraft K, Ak H, Davis AD, Lonn E, Miller R, Potter MA, Yusuf S, Anand SS, McQueen MJ (2015) The Relationship Between Trimethylamine-N-Oxide and Prevalent Cardiovascular Disease in a Multiethnic Population Living in Canada. Can J Cardiol 31:1189-1194.

117.	Guo F, Qiu X, Tan Z, Li Z, Ouyang D (2020) Plasma trimethylamine n-oxide is associated with renal function in patients with heart failure with preserved ejection fraction. BMC Cardiovasc Disord 20:394.

118.	Dong Z, Liang Z, Wang X, Liu W, Zhao L, Wang S, Hai X, Yu K (2020) The correlation between plasma trimethylamine N-oxide level and heart failure classification in northern Chinese patients. Ann Palliat Med 9:2862-2871.

119.	Dong Z, Zheng S, Shen Z, Luo Y, Hai X (2021) Trimethylamine N-Oxide is Associated with Heart Failure Risk in Patients with Preserved Ejection Fraction. Lab Med 52:346-351.

120.	Mafune A, Iwamoto T, Tsutsumi Y, Nakashima A, Yamamoto I, Yokoyama K, Yokoo T, Urashima M (2016) Associations among serum trimethylamine-N-oxide (TMAO) levels, kidney function and infarcted coronary artery number in patients undergoing cardiovascular surgery: a cross-sectional study. Clin Exp Nephrol 20:731-739.

121.	Wu C, Xue F, Lian Y, Zhang J, Wu D, Xie N, Chang W, Chen F, Wang L, Wei W et al. (2020) Relationship between elevated plasma trimethylamine N-oxide levels and increased stroke injury. Neurology 94:e667-e677.

122.	Li Z, Hui J, Li S, Cao T, Zhang J, Mao X, Wang F, Wang F, He P, You Y et al. (2023) Trimethylamine N-oxide predicts stroke severity in diabetic patients with acute ischaemic stroke and is related to glycemic variability. Eur J Neurol 30:3478-3486.

123.	Sheng Z, Tan Y, Liu C, Zhou P, Li J, Zhou J, Chen R, Chen Y, Song L, Zhao H et al. (2019) Relation of Circulating Trimethylamine N-Oxide With Coronary Atherosclerotic Burden in Patients With ST-segment Elevation Myocardial Infarction. Am J Cardiol 123:894-898.

124.	Tan Y, Sheng Z, Zhou P, Liu C, Zhao H, Song L, Li J, Zhou J, Chen Y, Wang L et al. (2019) Plasma Trimethylamine N-Oxide as a Novel Biomarker for Plaque Rupture in Patients With ST-Segment-Elevation Myocardial Infarction. Circ Cardiovasc Interv 12:e007281.

125.	Senthong V, Li XS, Hudec T, Coughlin J, Wu Y, Levison B, Wang Z, Hazen SL, Tang WH (2016) Plasma Trimethylamine N-Oxide, a Gut Microbe-Generated Phosphatidylcholine Metabolite, Is Associated With Atherosclerotic Burden. J Am Coll Cardiol 67:2620-2628.

126.	Huang PY, Hsu BG, Lai YH, Wang CH, Tsai JP (2023) Serum Trimethylamine N-Oxide Level Is Positively Associated with Aortic Stiffness Measured by Carotid-Femoral Pulse Wave Velocity in Patients Undergoing Maintenance Hemodialysis. Toxins (Basel) 15.

127.	Hobson S, Qureshi AR, Ripswedan J, Wennberg L, de LH, Ebert T, Soderberg M, Evenepoel P, Stenvinkel P, Kublickiene K (2023) Phenylacetylglutamine and trimethylamine N-oxide: Two uremic players, different actions. Eur J Clin Invest 53:e14074.

128.	He L, Yang W, Yang P, Zhang X, Zhang A (2022) Higher serum trimethylamine-N-oxide levels are associated with increased abdominal aortic calcification in hemodialysis patients. Ren Fail 44:2019-2027.

129.	Hsu BG, Wang CH, Lin YL, Lai YH, Tsai JP (2022) Serum Trimethylamine N-Oxide Level Is Associated with Peripheral Arterial Stiffness in Advanced Non-Dialysis Chronic Kidney Disease Patients. Toxins (Basel) 14.

130.	Zhang H, Yao G (2023) Significant correlation between the gut microbiota-derived metabolite trimethylamine-N-oxide and the risk of stroke: evidence based on 23 observational studies. Eur J Clin Nutr 77:731-740.

131.	Wang L, Nan Y, Zhu W, Wang S (2023) Effect of TMAO on the incidence and prognosis of cerebral infarction: a systematic review and meta-analysis. Front Neurol 14:1287928.

132.	Chen P, Guo Z (2022) Plasmatic trimethylamine N-oxide and its relation to stroke: A systematic review and dose-response meta-analysis. Medicine (Baltimore) 101:e29512.

133.	Farhangi MA, Vajdi M, Asghari-Jafarabadi M (2020) Gut microbiota-associated metabolite trimethylamine N-Oxide and the risk of stroke: a systematic review and dose-response meta-analysis. Nutr J 19:76.

134.	Khan, Q. A., Asad, M., Ali, A. H, Farrukh, A. M., Naseem, U., Semakieh, A., Carrion, Y. L., and Afzal, M. Gut microbiota metabolites and risk of major adverse cardiovascular events and death: A systematic review and meta-analysis. Medicine 103[22], e37825. 31-5-2024.

135.	Chen G, He L, Dou X, Liu T (2022) Association of Trimethylamine-N-Oxide Levels with Risk of Cardiovascular Disease and Mortality among Elderly Subjects: A Systematic Review and Meta-Analysis. Cardiorenal Med 12:39-54.

136.	Li X, Fan Z, Cui J, Li D, Lu J, Cui X, Xie L, Wu Y, Lin Q, Li Y (2022) Trimethylamine N-Oxide in Heart Failure: A Meta-Analysis of Prognostic Value. Front Cardiovasc Med 9:817396.

137.	Li W, Huang A, Zhu H, Liu X, Huang X, Huang Y, Cai X, Lu J, Huang Y (2020) Gut microbiota-derived trimethylamine N-oxide is associated with poor prognosis in patients with heart failure. Med J Aust 213:374-379.

138.	Qi J, You T, Li J, Pan T, Xiang L, Han Y, Zhu L (2018) Circulating trimethylamine N-oxide and the risk of cardiovascular diseases: a systematic review and meta-analysis of 11 prospective cohort studies. J Cell Mol Med 22:185-194.

139.	Farhangi MA (2020) Gut microbiota-dependent trimethylamine N-oxide and all-cause mortality: Findings from an updated systematic review and meta-analysis. Nutrition 78:110856.

140.	Schiattarella GG, Sannino A, Toscano E, Giugliano G, Gargiulo G, Franzone A, Trimarco B, Esposito G, Perrino C (2017) Gut microbe-generated metabolite trimethylamine-N-oxide as cardiovascular risk biomarker: a systematic review and dose-response meta-analysis. Eur Heart J 38:2948-2956.
